# Artificial intelligence in central nervous system autoimmune diseases: from clinical decision support to mechanistic discovery and translation

**DOI:** 10.1038/s44321-026-00527-0

**Published:** 2026-10-13

**Authors:** Zijian Cao, Pei Zheng, Zhihui Qi, Yang Yao, Donglin Xie, Yazi He, Shenghua Zong, Zongjiu Zhang, Fu-Dong Shi

**Affiliations:** 1 School of Biomedical Engineering, Tsinghua University, Beijing, China; 2 Institute for Hospital Management of Tsinghua University, Tsinghua University, Shenzhen, China; 3 Departments of Neurology, Beijing Tiantan Hospital, Capital Medical University, Beijing, China; 4 Departments of Neurology, Tianjin Medical University General Hospital, Tianjin, China; 5 National Institute of Health Data Science, Peking University, Beijing, China; 6 KingMed Diagnostic Laboratory, Guangzhou, China

**Keywords:** Computational Biology, Immunology, Neuroscience

## Abstract

Central nervous system (CNS) autoimmune diseases, including multiple sclerosis (MS), neuromyelitis optica spectrum disorder (NMOSD), autoimmune encephalitis (AE), and myelin oligodendrocyte glycoprotein antibody-associated disease (MOGAD), remain difficult to diagnose, stratify, and treat because clinical phenotypes overlap, biomarkers are imperfect, and the disease course requires longitudinal observation. Artificial intelligence (AI) is increasingly being evaluated to integrate clinical, imaging, immunological, and molecular data for decision-making in neuroimmunology. Clinical applications include automated neuroimaging analysis, differential diagnosis, disease classification, relapse prediction, prognostic stratification, and risk-informed care planning. Evidence is unevenly distributed: MS has the largest body of published work, particularly in neuroimaging, whereas studies in NMOSD, AE, and MOGAD are smaller, more heterogeneous, and often lack external validation. Computational approaches are also being applied to multi-omics, single-cell and spatial data, molecular target prioritisation, drug repositioning, trial enrichment, digital biomarkers, federated learning, and longitudinal modelling; however, these applications generally provide association, prioritisation, or hypothesis-generating evidence rather than validated therapeutic benefit. This Review maps reported applications, evidence patterns, and methodological and implementation needs rather than ranking translational maturity or implying established clinical benefit.

## Abstract (web summary)

In this review, FD. Shi, Z. Zhang and colleagues discuss the emerging applications of artificial intelligence in the diagnosis, stratification, and mechanistic investigation of central nervous system autoimmune diseases. They highlight current advances, methodological limitations, and future priorities for integrating multimodal clinical and molecular data to advance precision neuroimmunology.

## Graphical abstract

**Figure:**
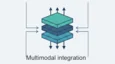

## Introduction

Central nervous system (CNS) autoimmune diseases, including multiple sclerosis (MS), neuromyelitis optica spectrum disorder (NMOSD), autoimmune encephalitis (AE), and myelin oligodendrocyte glycoprotein antibody-associated disease (MOGAD), are heterogeneous immune-mediated disorders marked by relapsing neurological injury, long-term disability, and diagnostic and therapeutic uncertainty. Their overlapping and often atypical presentations require integration of neuroimaging, cerebrospinal fluid and serum biomarkers, electrophysiology, clinical phenotype, treatment response, and longitudinal disease evolution. Consequently, misdiagnosis, delayed recognition, uncertain prognosis, and difficulty selecting or adjusting treatment remain common (Bagherieh et al, 2023; Dubey et al, 2018; Flanagan et al, 2023).

Artificial intelligence (AI) is increasingly being evaluated as a tool for this evidence-intensive process. Early applications focused on automated neuroimaging, particularly lesion segmentation and MRI-based monitoring in MS (Amin et al, 2024; Ronneberger et al, 2015; LeCun et al, 2015; Isensee et al, 2021). More recent studies integrate imaging with laboratory biomarkers, electronic health records, electrophysiology, and longitudinal outcomes to support differential diagnosis, disease classification, relapse prediction, prognostic stratification, and risk-informed monitoring or care planning (Thakur et al, 2022; Lodé et al, 2025; Gaj et al, 2021; Luo et al, 2024; Mizoguchi et al, 2022; David Cancino et al, 2025; Klistorner et al, 2022; Kodosaki et al, 2024; Huang et al, 2020; Chase et al, 2017; Nelson et al, 2016). Computational approaches are also being used in molecular and translational research, but these applications generally provide association, target prioritisation, or hypothesis-generating evidence rather than validated therapeutic benefit (Yang et al, 2025; Villar-Delfino et al, 2025; Elkjaer et al, 2024; Acquaviva et al, 2020; Hossain et al, 2022; Yang et al, 2025; Heming et al, 2022; Zhou et al, 2024; Yu et al, 2024). The evidence base remains uneven, with the largest body of work in MS and smaller, more heterogeneous studies in NMOSD, AE, and MOGAD.

The clinical uncertainties relevant to AI development differ across these disorders. In MS, inflammatory activity and relapse-independent progression are incompletely captured by current imaging and fluid biomarkers. In NMOSD and MOGAD, seronegative disease, phenotypic overlap, relapse risk, and recovery after attacks remain difficult to predict. AE is characterised by heterogeneity in antibody status, MRI and EEG findings, treatment response, and cognitive outcomes. These unresolved questions support the evaluation of multimodal and longitudinal models, although the available evidence remains uneven across diseases and clinical tasks.

This Review therefore maps reported applications, evidence patterns, and methodological needs rather than ranking translational maturity or implying established clinical benefit. Sections “Current clinical landscape and unmet decision-making needs in CNS autoimmune diseases”, “Data sources and AI methods” and “Clinical decision points for AI support in CNS autoimmune diseases” address the clinical landscape, data and methods, and patient-level decision points; “Computational molecular discovery and translational research in CNS autoimmunity” addresses molecular discovery and trial translation; “Disease-specific evidence and translational gaps” synthesises disease-specific evidence; and “Conclusions and future directions” discusses future research and implementation priorities.

## Review scope and evidence identification

This article is a structured narrative review informed by a documented literature search; it is not a formal systematic review, scoping review, or meta-analysis. PubMed, Embase, Web of Science, and IEEE Xplore were searched from January 1, 2010 through July 31, 2026 using combinations of terms for multiple sclerosis, neuromyelitis optica spectrum disorder, autoimmune encephalitis, and myelin oligodendrocyte glycoprotein antibody-associated disease, with terms related to artificial intelligence, machine learning, deep learning, radiomics, multimodal modelling, foundation models, and relevant clinical and translational applications. Reference lists of relevant reviews and included articles were also examined.

One author conducted the initial screening and selection of relevant publications and extracted key study information. The selected studies and extracted evidence were subsequently reviewed by the author group, and uncertainties regarding study relevance, methodological classification, or evidence interpretation were resolved through discussion and consensus. Original human studies evaluating defined diagnostic, prognostic, monitoring, or treatment-response tasks using patient-level data were prioritised for the disease-specific evidence tables. Reviews, consensus criteria, reporting guidelines, foundational methodological studies, and molecular-discovery research were included when necessary for clinical, methodological, or translational context. The synthesis was qualitative and should not be interpreted as an exhaustive catalogue of the literature.

## Current clinical landscape and unmet decision-making needs in CNS autoimmune diseases

Despite advances in antibody testing, imaging criteria, and disease classification, key clinical decisions remain unresolved, including recognition of atypical presentations, discrimination from mimics, and prediction of relapse, recovery, disability progression, and treatment response (Bagherieh et al, 2023; Dubey et al, 2018; Banwell et al, 2023; Flanagan et al, 2023; Amin et al, 2024; Seccia et al, 2021; Graus et al, 2016). Relevant signals are distributed across MRI, OCT, electrophysiology, serum and CSF biomarkers, treatment exposure, and longitudinal clinical outcomes. CNS autoimmune disease management is therefore increasingly a multimodal and longitudinal inference problem rather than a single-biomarker classification task.

In MS, the principal unresolved issue is the distinction between inflammatory disease activity and progression that occurs independently of clinical relapse. Conventional MRI and fluid biomarkers provide important information but do not fully capture compartmentalised inflammation, neuroaxonal injury, cognitive decline, or long-term disability trajectories. Automated lesion segmentation, lesion quantification, central vein sign and paramagnetic rim lesion assessment, and longitudinal disability prediction may reduce measurement burden and improve phenotyping. However, the clinical value of these tools depends on external validation, appropriate reference standards, and evidence that their use changes diagnostic or treatment decisions (Amin et al, 2024; Seccia et al, 2021; Montalban et al, 2025; Chen et al, 2024; Manning et al, 2025).

In NMOSD, AQP4-IgG remains central to disease classification, but seronegative presentations, atypical phenotypes, overlap with other demyelinating disorders, and coexistence with systemic autoimmune diseases such as Sjögren disease and systemic lupus erythematosus complicate diagnostic attribution (Moseley et al, 2026; Attfield et al, 2026). Clinically important prediction tasks include relapse risk, attack severity, recovery, and treatment response. Current studies are concentrated in AQP4-IgG-positive cohorts and often use typical MS comparators, leaving uncertainty about transportability to seronegative, overlap, and diagnostically uncertain populations (He et al, 2022; Wang et al, 2022).

MOGAD is biologically and clinically heterogeneous, with variation according to age, presenting syndrome, monophasic or relapsing course, and the extent of recovery after an attack. The 2023 international diagnostic criteria were proposed by Banwell et al (2023) and subsequently validated in a selected adult and paediatric cohort by Varley et al (2024), improving consistency in cohort definition. However, relapse prediction, recovery prediction, and decisions about maintenance therapy remain difficult (Xu et al, 2024; Yeh et al, 2026). Early pathological observations, including complement deposition and oligodendrocyte loss in severe disease, are hypothesis-generating and do not yet establish clinically validated predictors of outcome (Takai et al, 2026).

AE encompasses antibody-defined and seronegative syndromes with heterogeneous clinical presentations, MRI and EEG findings, treatment responses, and cognitive or functional outcomes. Important unmet needs include early recognition of patients requiring intensive care, prediction of refractory disease, assessment of treatment response, and estimation of long-term cognitive recovery. These tasks are complicated by variable antibody status, evolving diagnostic criteria, heterogeneous outcome definitions, and relatively small cohorts (Graus et al, 2016; Flanagan et al, 2023; Xiang et al, 2022a; Sun et al, 2025a).

**Fig. 1:**
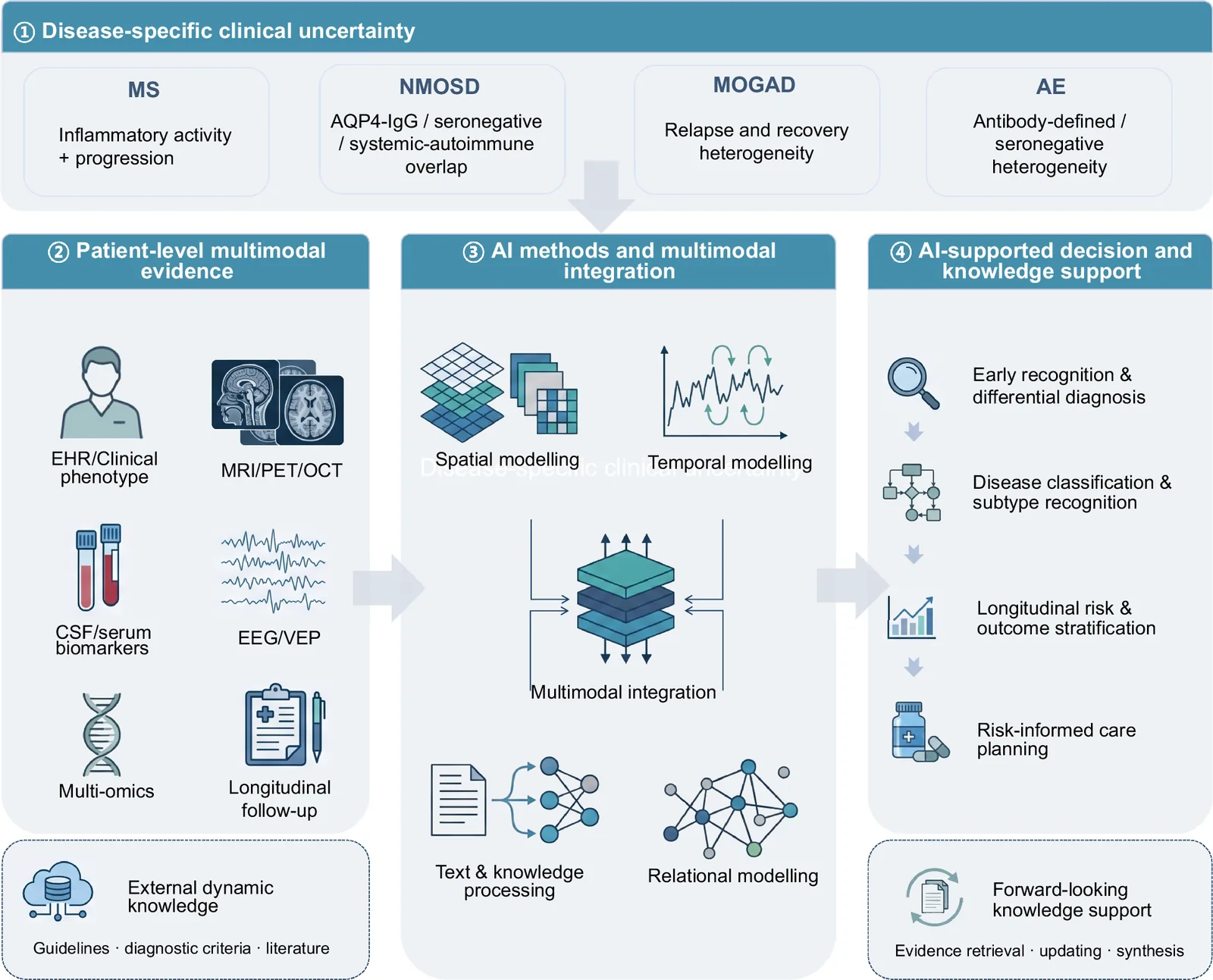
Disease-specific clinical needs and multimodal AI-enabled decision support in CNS autoimmune diseases. The framework links disease-specific clinical uncertainty with patient-level multimodal evidence, external dynamic knowledge, complementary AI methods, and downstream clinical support functions. Disease-specific uncertainties include inflammatory activity and progression in MS; AQP4-IgG status, seronegative disease, and systemic-autoimmune overlap in NMOSD; antibody-defined and seronegative heterogeneity in AE; and relapse and recovery heterogeneity in MOGAD. Patient-level evidence includes clinical phenotype and electronic health records, neuroimaging, cerebrospinal fluid and serum biomarkers, electrophysiology, multi-omics, and longitudinal follow-up, whereas external dynamic knowledge includes guidelines, diagnostic criteria, and the scientific literature. Spatial, temporal, relational, text- and knowledge-processing, and multimodal-integration approaches can support earlier recognition and differential diagnosis, disease classification and subtype recognition, longitudinal risk and outcome stratification, and risk-informed care planning. Forward-looking knowledge-augmented systems may additionally support evidence retrieval, updating, and synthesis, but are distinguished from established clinical decision support. AE autoimmune encephalitis, AQP4-IgG aquaporin-4 immunoglobulin G, CSF cerebrospinal fluid, EEG electroencephalography, EHR electronic health record, MRI magnetic resonance imaging, MOGAD myelin oligodendrocyte glycoprotein antibody-associated disease, MS multiple sclerosis, NMOSD neuromyelitis optica spectrum disorder, OCT optical coherence tomography, PET positron emission tomography, VEP visual evoked potential.

Across these disorders, the relevant decision points are therefore not limited to diagnosis. They include differential diagnosis, disease classification, longitudinal risk stratification, monitoring of treatment response, and risk-informed care planning (Fig. 1). These clinical needs define the data and methodological requirements discussed in “Data sources and AI methods” and “Clinical decision points for AI support in CNS autoimmune diseases”. Disease-specific empirical evidence is mapped in “Disease-specific evidence and translational gaps”, whereas molecular discovery and trial translation are addressed separately in “Computational molecular discovery and translational research in CNS autoimmunity”.

## Data sources and AI methods

### Methodological taxonomy and data modalities

In this Review, artificial intelligence (AI) is used as an umbrella term encompassing machine learning, deep learning, and generative or foundation-model approaches. Conventional statistical and regression modelling, causal-inference methods, and standard bioinformatic workflows are described separately by their methodological characteristics and are not classified as AI per se. The taxonomy applied throughout the Review is summarised in Box 1.

CNS autoimmune diseases generate heterogeneous data streams that differ in scale, temporal structure, missingness, and measurement reliability. Table 1 summarises the principal data layers, representative computational approaches, multimodal integration strategies, illustrative clinical roles, and major integration limitations. MRI currently dominates the empirical evidence base, whereas electrophysiology, EHR-derived text, digital biomarkers, multi-omics, and dynamic knowledge layers remain less extensively validated.

**Table 1:** Data layers, multimodal integration strategies, and representative computational applications in CNS autoimmune disease research.

Data layer/integration strategy	Representative sources or approach	Typical computational methods	Illustrative clinical role	Principal limitation or integration issue
Neuroimaging	MRI (T1/T2/FLAIR/DWI), PET, OCT	CNN, U-Net/nnU-Net, vision Transformer	Lesion segmentation and volumetry; disease differentiation; structural and longitudinal monitoring	Scanner and protocol heterogeneity; annotation variability; specialised acquisition for selected biomarkers
Neurophysiology and digital signals	EEG, VEP, wearable-derived biosignals	Temporal CNN, LSTM, Transformer	Encephalitis pattern recognition; longitudinal functional monitoring; digital phenotyping	Artefact sensitivity; variable acquisition; asynchronous sampling; limited disease-specific validation
Clinical text and structured EHR data	Clinical notes, discharge summaries, diagnoses, medications, longitudinal records	NLP, BERT-family models, information extraction, structured ML	Early-warning screening; phenotype extraction; longitudinal risk modelling	Missingness; site-specific documentation; temporal leakage; transportability across healthcare systems
Biomarkers and laboratory data	Serum/CSF antibodies, antibody titres, NfL, cytokines, routine laboratory variables	ML methods (e.g. random forest and XGBoost) and regularised regression	Diagnostic classification; disease-activity and risk stratification; treatment-response prediction	Assay heterogeneity; sampling-time effects; treatment-related confounding; uncertain incremental value
Omics and molecular data	Genomics, transcriptomics, proteomics, scRNA-seq, spatial and multi-omics data	GNN, VAE, representation learning, multi-omics integration	Molecular stratification; mechanistic hypothesis generation; target prioritisation	High dimensionality; batch effects; small cohorts; limited external and experimental validation
Dynamic knowledge layer	Diagnostic criteria, guidelines, literature, disease ontologies	Retrieval-augmented models, knowledge graphs, LLMs and agentic systems	Evidence retrieval and synthesis; criteria updating; source-grounded knowledge support	Source traceability; version control; hallucination; accountability; no prospective disease-specific validation
Early fusion	Concatenation of pre-aligned features before model fitting	Feature-level multimodal ML/DL	Multimodal diagnosis and risk prediction using jointly available structured inputs	Sensitive to scaling, missing modalities, asynchronous sampling, and temporal leakage
Joint/intermediate fusion	Modality-specific encoders with shared or cross-modal representations	Attention-based fusion, multimodal Transformer, joint representation learning	Learning interactions across imaging, clinical, biomarker, and longitudinal data	Data-intensive; susceptible to dominant-modality effects; uncertainty propagation and missing-modality handling remain challenging
Late/decision fusion	Combination of predictions from separately trained modality-specific models	Ensembles, stacking, weighted decision fusion	Modular multimodal decision support; integration when modality availability varies	May underuse cross-modal interactions; requires calibration across component models and explicit handling of unavailable modalities

*CNN* convolutional neural network, *CSF* cerebrospinal fluid, *DWI* diffusion-weighted imaging, *EEG* electroencephalography, *EHR* electronic health record, *GNN* graph neural network, *LLM* large language model, *LSTM* long short-term memory, *MRI* magnetic resonance imaging, *NfL* neurofilament light chain, *NLP* natural language processing, *OCT* optical coherence tomography, *PET* positron emission tomography, *scRNA-seq* single-cell RNA sequencing, *VAE* variational autoencoder, *VEP* visual evoked potential.

Fusion categories describe the stage at which information from different modalities is combined and do not represent evidence-readiness or clinical-maturity levels.

Multimodal data may be acquired at the same clinical encounter or asynchronously over longitudinal follow-up. Fusion strategies should therefore be selected according to the clinical index time, modality availability, and expected pattern of missingness. Models should be evaluated under realistic availability patterns, with attention to temporal leakage, treatment exposure, cross-centre transportability, uncertainty, and potential model drift (Havaei et al, 2016; Acosta et al, 2022; Fortin et al, 2017).

Box 1 Methodological taxonomy used in this Review

**Table:** 

Methodological category	Definition and use in this Review
Statistical and regression modelling	Conventional approaches such as logistic regression, Cox regression, regularised regression, and regression-based nomograms. These methods are referred to by their specific methodological names and are not classified as AI per se.
Machine learning (ML)	Data-driven algorithms that learn predictive patterns without relying solely on prespecified parametric relationships. Examples include random forests, gradient-boosting methods, support vector machines, and k-nearest-neighbour algorithms.
Deep learning (DL)	A subset of machine learning based on multilayer neural networks that learn hierarchical representations from high-dimensional data. Examples include convolutional neural networks, U-Net architectures, recurrent neural networks, Transformers, graph neural networks, and variational autoencoders.
Generative and foundation-model AI	Large pretrained models that can be adapted across tasks or generate new outputs. This category includes large language models, vision–language models, retrieval-augmented generation systems, and agentic AI. These approaches are treated as a distinct, predominantly emerging or forward-looking class in this Review.
Causal inference and bioinformatic approaches	Methods such as Mendelian randomisation, colocalisation, differential-expression analysis, enrichment analysis, pseudotime analysis, and ligand–receptor inference. These approaches can complement AI-based research but are described as causal-inference, bioinformatic, or AI-adjacent methods rather than AI per se.

AI is used as an umbrella term that includes machine learning and deep learning, whereas conventional statistical modelling, causal-inference methods, and standard bioinformatic workflows are distinguished from AI throughout this Review. Classification is based on methodological characteristics rather than on whether a study was described by its original authors as “AI”.

### Core architectures and learning paradigms

The dominant paradigm across CNS autoimmune disease AI research is supervised learning, in which labelled examples—expert-annotated MRI scans, confirmed diagnoses, or structured outcome data—train models to map inputs to clinical predictions (Isensee et al, 2021). Its primary limitation is dependence on large, high-quality annotated datasets, which are scarce for low-prevalence conditions such as AE and MOGAD. Unsupervised, semi-supervised, and transfer-learning approaches can reduce dependence on fully annotated datasets. Foundation models extend this principle through large-scale pre-training on unlabelled, weakly labelled, or multimodal data, followed by task-specific adaptation rather than de novo model development. Their potential relevance and current limitations in rare CNS autoimmune diseases are discussed in “Forward-looking foundation models and medical large language models”.

At the architecture level, convolutional neural networks (CNNs) and their segmentation variants—most prominently U-Net (Ronneberger et al, 2015) and the self-configuring nnU-Net (Isensee et al, 2021)—remain the standard for spatial feature extraction from MRI. Transformer-based models, originally developed for sequence modelling (Vaswani et al, 2017) and subsequently extended to vision tasks (Vision Transformer, ViT; Dosovitskiy et al, 2021), capture long-range dependencies that local convolutional operations miss, making them well-suited to whole-brain lesion distribution analysis. For temporal data—longitudinal disability trajectories or EEG signals—recurrent networks including LSTM (Hochreiter and Schmidhuber, 1997) have been broadly applied, though Transformer-based time-series models are increasingly preferred. Natural language models can support information extraction and representation learning from unstructured clinical records (Devlin et al, 2019), although disease-specific validation remains limited. Graph neural networks (GNNs; Scarselli et al, 2009) offer a principled framework for relational data such as brain functional connectomes and protein–protein interaction networks, supporting both connectome-based biomarker discovery and drug–target identification.

### Trustworthiness and privacy-preserving computation

Clinical trustworthiness is multidimensional and cannot be established through interpretability alone. Five elements should be evaluated. First, interpretability methods such as Grad-CAM and SHAP can support model inspection, but their stability, clinical plausibility, and sensitivity to model or input perturbations should be tested rather than assumed (Lundberg and Lee, 2017; Selvaraju et al, 2020). Second, calibration should be assessed using calibration plots, calibration intercept and slope, Brier scores, or expected calibration error, ideally within external cohorts and clinically relevant risk ranges. Third, uncertainty should be quantified through prediction intervals, conformal prediction, ensembles, or other methods that permit abstention when confidence is insufficient. Fourth, robustness should be examined across scanners, centres, acquisition protocols, missing-data patterns, and plausible input perturbations. Fifth, fairness requires reporting subgroup-specific discrimination, calibration, error rates, and uncertainty across demographic and clinically relevant populations. None of these properties substitutes for geographically external and prospective validation, which remains necessary to determine whether performance is maintained in clinical workflows.

Trustworthiness also requires appropriate data governance. The rarity of CNS autoimmune diseases necessitates multicentre data pooling, yet inter-institutional data sharing is constrained by privacy regulation, including China’s Personal Information Protection Law and the General Data Protection Regulation. Federated learning enables distributed model training without centralising raw patient data (McMahan et al, 2017; Rieke et al, 2020), but the evidence within CNS autoimmunity is disease-dependent. The BraTS initiative demonstrates technical feasibility in brain-tumour segmentation and should not be treated as direct neuroimmunology evidence (Pati et al, 2022). Direct research evidence is available in MS, including federated lesion-segmentation studies using multicentre clinical datasets and personalised federated learning for two-year disability-progression prediction (Bai et al, 2024; Pirmani et al, 2025; Hindawi et al, 2025). These studies demonstrate research feasibility rather than routine clinical implementation. No disease-specific federated-learning study in NMOSD, MOGAD, or AE was identified in our search; application to these rarer disorders therefore remains forward-looking.

### Methodological quality, sources of bias, and reporting standards

Systematic appraisals of AI research in CNS autoimmune diseases indicate that high reported performance is often accompanied by design features that increase the risk of bias (Nabizadeh et al, 2023; Vázquez-Marrufo et al, 2023; Bonacchi et al, 2022). Spectrum bias arises when models are developed using well-characterised cases and healthy controls, or a restricted set of mimics, rather than patients presenting with real-world diagnostic uncertainty. Data leakage occurs when information from the same patient, examination, clinical site, follow-up period, preprocessing procedure, or feature-selection process influences both model development and evaluation. This is especially important when multiple lesions, scans, treatment episodes, or visits from one patient are analysed as independent observations.

Overfitting is amplified by small, rare-disease cohorts, high-dimensional radiomic or omics features, extensive model selection, and the absence of nested or independent validation. Annotation variability creates additional uncertainty because lesion boundaries, imaging signs, clinical outcomes, and sometimes diagnostic labels depend on reader judgment. Random internal splitting does not establish transportability across scanners, acquisition protocols, centres, demographic groups, age ranges, referral pathways, or healthcare systems. Appraisal should also consider publication bias and the underreporting of negative findings and failed external validations, particularly in rare diseases and paediatric cohorts. Table 2 provides a qualitative cross-application mapping of the highest type of evidence identified in this Review; the stages do not represent the volume, consistency, or overall quality of evidence, clinical maturity, or demonstrated patient benefit.

**Table 2:** Evidence-stage mapping of major AI application domains in CNS autoimmune diseases.

Application domain	Representative evidence in this Review	Highest evidence stage represented	Interpretive status	Principal translational bottleneck
Automated MRI lesion segmentation and volumetric quantification	Large retrospective datasets, external benchmarks, multicentre prospective workflow studies, and selected implementation reports, predominantly in MS	Stage 4	Workflow-evaluated/implemented in selected settings	Scanner and protocol drift; small or atypical lesions; workflow interoperability; limited evidence that use changes clinical decisions or patient outcomes
Longitudinal imaging and disease-activity monitoring	Multicentre and prospective studies, mainly in MS; substantially less disease-specific evidence in NMOSD, MOGAD, and AE	Stage 4	Workflow-evaluated in selected MS settings; earlier-stage elsewhere	Harmonised serial acquisition; reliable change detection; treatment-related confounding; prospective clinical-impact evaluation
Diagnostic classification and differential diagnosis	Numerous retrospective studies and selected multicentre or geographically external validations, particularly for demyelinating disease differentiation	Stage 3	Validated research application in selected tasks	Spectrum bias; restricted mimic groups; site and scanner confounding; uncertain performance in consecutive diagnostically ambiguous patients
Specialised imaging biomarkers, including CVS, PRLs, OCT, and spinal-cord features	Multicentre CVS/PRL studies and international ophthalmic cohorts, although much of the evidence remains retrospective	Stage 3	Validated research in selected imaging tasks; not established for routine use	Specialised acquisition; annotation variability; device and protocol heterogeneity; limited prospective workflow validation
Relapse, progression, recovery, and functional-outcome prediction	Predominantly retrospective studies, with selected multicentre or externally validated prognostic cohorts	Stage 3	Validated prognostic research in selected settings	Calibration; temporal leakage; treatment exposure; outcome-definition variability; limited evidence of decision benefit
Digital phenotyping and remote monitoring	Wearable-, voice-, and patient-generated-data studies, including selected independent validation	Stage 3	Early validated research; predominantly exploratory	Missingness and adherence; device and language effects; digital-access bias; longitudinal drift; uncertain incremental value over routine assessment
Risk-informed care planning	Prognostic models identify patients at higher risk under observed clinical care, including selected externally validated risk models	Stage 3	Prognostic research only; not validated for treatment selection	Predicted risk does not estimate comparative treatment benefit; confounding by indication; limited prospective decision-impact evidence
Comparative treatment-effect estimation and individualised therapy selection	No established disease-specific model was identified for estimating comparative treatment benefit or selecting/sequencing therapies across the reviewed diseases	Stage 1	Conceptual/unmet application	Causal estimands; treatment heterogeneity; time-varying exposure and confounding; prospective interventional validation
AI-assisted molecular discovery and target prioritisation	Multi-omics integration, computational target nomination, and causal-prioritisation studies, with limited experimental validation	Stage 2	Early empirical discovery application	Distinguishing association from causality; experimental replication; druggability; preclinical and clinical efficacy validation
AI-assisted clinical-trial enrichment and candidate endpoint development	Computational prognostic stratification, candidate quantitative outcome measures, and methodological proposals; no established prospective trial-utility evidence	Stage 2	Early empirical/hypothesis-generating	Validation of enrichment variables; endpoint qualification; treatment-effect interpretation; selection bias; prospective trial utility
Foundation models and medical LLMs	Predominantly adjacent biomedical foundation-model evidence plus limited disease-specific LLM proof-of-concept evidence in AE; no prospective CNS autoimmune disease validation	Stage 2	Early empirical/forward-looking	Domain and modality shift; rare-disease under-representation; hallucination; calibration; disease-specific benchmarks; computational and regulatory requirements
Dynamic knowledge updating and agentic decision support	Conceptual framework and evidence from adjacent rare-disease systems; no prospective CNS autoimmune disease-specific validation	Stage 1	Forward-looking/conceptual	Source traceability; version control; hallucination; accountability; workflow governance; prospective safety and effectiveness testing

*AE* autoimmune encephalitis, *AI* artificial intelligence, *CVS* central vein sign, *LLM* large language model, *MOGAD* myelin oligodendrocyte glycoprotein antibody-associated disease, *MRI* magnetic resonance imaging, *MS* multiple sclerosis, *NMOSD* neuromyelitis optica spectrum disorder, *OCT* optical coherence tomography, *PRL* paramagnetic rim lesion.

Stages indicate the highest type of evidence represented in this Review for each application domain and do not reflect the volume, consistency, overall quality, clinical readiness, or demonstrated patient benefit of the evidence. Stage 1, conceptual: no direct disease-specific empirical evaluation; Stage 2, early empirical: disease-specific proof-of-concept or predominantly retrospective development/internal validation; Stage 3, validated research: independent, temporal, geographically external, multicentre, or prospective validation; Stage 4, workflow-evaluated or implemented: evaluation within a clinical workflow or documented implementation. A higher stage does not imply regulatory authorisation, routine adoption, reimbursement, superiority to standard care, or demonstrated improvement in patient outcomes.

Accordingly, AUC and Dice scores should be interpreted alongside confidence intervals, calibration, clinically relevant sensitivity and specificity, predictive values under realistic disease prevalence, comparator performance, and structured error analysis. Appraisal should establish whether data were partitioned at the patient level, whether preprocessing and feature selection were confined to the training data, whether annotation reliability was assessed, and whether validation was internal, temporal, geographically external, or prospective. Design-appropriate reporting frameworks are summarised in Table EV1 (Mongan et al, 2020; Sounderajah et al, 2025; Liu et al, 2020; Cruz Rivera et al, 2020; Vasey et al, 2022; Norgeot et al, 2020). Study design, validation, performance, comparator, and limitation information for representative disease-specific AI studies is summarised in Tables 3–6, with extended extraction in Tables EV2–5. Information unavailable in the original publications is reported as not reported rather than inferred.

**Table 3:** Representative AI studies in multiple sclerosis: design, validation, performance, and methodological limitations.

Application and key study	Cohort and unit of analysis	Design and validation	Performance and comparator	Key limitations and interpretation
MS lesion segmentation—Gabr et al, 2020	1,003 baseline MRI datasets from RRMS participants in the multicentre CombiRx trial	FCNN/U-Net; train/validation/test split plus leave-one-centre-out cross-validation within CombiRx	T2-lesion Dice 0.82 in the test set and 0.85 in leave-one-centre-out validation	Large multicentre dataset supports technical feasibility, but evaluation remained within a single trial protocol and does not establish transportability to routine heterogeneous MRI
Lesion segmentation with external benchmark—Wiltgen et al, 2024	491 development cases; external evaluation on MSSEG-1	3D U-Net ensemble (LST-AI); external benchmark evaluation	MSSEG-1 Dice 0.65; lesion detection >75% for lesions >10 mm³; compared with LST, SAMSEG, and nnU-Net	External benchmarking strengthens reproducibility evidence, but performance remains lesion-size dependent and prospective clinical impact was not evaluated
Prospective disease-activity monitoring—Barnett et al, 2023	397 paired MRI examinations; longitudinal patient-level assessment	Multicentre prospective workflow evaluation	Sensitivity 93.3% versus 58.3% for conventional reporting; volumetry comparable with core-laboratory assessment	Among the stronger translational studies, but effects on treatment decisions, workflow burden, and patient outcomes remain uncertain
Automated PRL detection —Chen et al, 2024	92 participants; 5,637 white-matter lesions, including 148 manually identified PRLs	Multicentre 3-T MRI validation against expert-consensus labels	Detected 115/148 PRLs (78%); lesion-level AUC 0.73 (95% CI 0.68–0.78); subject-level AUC 0.69 (0.57–0.81)	Moderate discrimination, specialised susceptibility imaging, false-positive/missed lesions, and expert-label dependence limit routine implementation
Automated CVS assessment— Manning et al, 2025	86 participants evaluated for MS across nine sites; automated processing successful in 79	Multicentre diagnostic evaluation against simplified and full manual CVS assessment	Automated AUC 0.78 (95% CI 0.67–0.88), versus 0.80 for simplified manual counting and 0.89 for full manual assessment	Motion and susceptibility-sensitive acquisition affected applicability; larger prospective cohorts representing the full spectrum of suspected MS are needed
Two-year disability and cognitive progression—Storelli et al, 2022	373 patients; baseline MRI linked to two-year clinical and cognitive outcomes	Deep-learning MRI prognostic model; independent 48-patient test set from another centre/scanner	External accuracy 83.3% for clinical worsening, 67.7% for cognitive worsening, and 85.7% for the joint EDSS/SDMT outcome; physician accuracy 70.0%	Independent testing is a strength, but the external cohort was small; calibration, broader transportability, and treatment-exposure handling remain limitations
Fingolimod treatment-response prediction—Ferrè et al, 2023	381 fingolimod-treated RRMS patients; independent test set, *n* = 77	Random-forest clinical/genetic models using Italian and French cohorts	Combined model AUC 0.71 and AUPRC 0.73; clinical-only AUC 0.69; genetic-only AUC 0.65	Modest discrimination and a small independent test set support proof of concept only; outcome prediction does not establish individual treatment effect or therapy selection
Voice-based digital biomarker—Delgado Hernández et al, 2026	300 participants; independent clinical validation cohort, *n* = 100	Acoustic-feature ML pipeline with independent validation	Validation AUC 0.85 (95% CI 0.76–0.93); sensitivity and specificity 0.80	Low-burden acquisition is attractive, but healthy-control comparison, language, recording environment, and device effects limit diagnostic generalisability

*AUC* area under the receiver–operating characteristic curve, *AUPRC* area under the precision–recall curve, *CI* confidence interval, *CVS* central vein sign, *EDSS* Expanded Disability Status Scale, *FCNN* fully convolutional neural network, *MRI* magnetic resonance imaging, *MS* multiple sclerosis, *PRL* paramagnetic rim lesion, *RRMS* relapsing-remitting multiple sclerosis, *SDMT* Symbol Digit Modalities Test.

Performance estimates and validation details are reported as described in the cited studies. NR indicates not reported. The final column provides a narrative, design-based appraisal and should not be interpreted as a formal risk-of-bias assessment.

**Table 4:** Representative AI studies in neuromyelitis optica spectrum disorder: design, validation, performance, and methodological limitations.

Application and key study	Cohort and unit of analysis	Design and validation	Performance and comparator	Key limitations and interpretation
Multicentre MRI differentiation—He et al, 2022	223 patients from four centres plus 74 external cases from three centres	Random-forest radiomics model; multicentre development with geographically external validation	AUC 0.944 in the internal test set and 0.902 externally; external sensitivity 79.7% and specificity 86.0%	Geographically external validation strengthens evidence, but retrospective selection, scanner/protocol heterogeneity, treatment effects, and absent calibration reporting limit clinical interpretation
Three-disease MRI classification—Huang et al, 2022	290 patients: 67 MS, 162 AQP4-IgG-positive NMOSD, and 61 MOGAD	MIL-CoaT Transformer using brain/spinal-cord MRI; retrospective single-centre development and held-out testing	NMOSD AUC 0.942 (95% CI 0.879–0.987); multiclass accuracy 81.4% versus 64.4% and 74.6% for two neuroradiologists	Strong internal discrimination, but performance varied by disease and geographically external validation in consecutive diagnostically uncertain patients is lacking
Seronegative NMOSD classification—Cacciaguerra et al, 2022	228 MRI examinations, including 85 AQP4-IgG-positive NMOSD, 95 MS, 11 seronegative NMOSD, and 37 seronegative NMOSD-like cases	3D CNN developed on AQP4-IgG-positive NMOSD versus MS and applied to seronegative/ambiguous presentations	Accuracy 0.95 for AQP4-IgG-positive NMOSD versus MS; all 11 seronegative NMOSD cases were classified as NMOSD	Clinically relevant extension to seronegative disease, but subgroup sizes were very small and diagnostic-label uncertainty limits conclusions
Multicentre relapse-risk prediction—Wang et al, 2022	1135 treatment episodes from 358 patients for development; 213 episodes from 92 patients across nine centres for validation	Retrospective multicentre survival modelling comparing Cox-PH, RSF, LogisticHazard, DeepHit, and DeepSurv	Validation C-index 0.718 for LogisticHazard versus 0.651 for Cox-PH	Multicentre validation is a strength, but episode-level modelling, retrospective treatment exposure, dosing heterogeneity, and absence of prospective validation limit use; the prognostic importance of therapy variables does not establish treatment effect
Serum FTIR spectroscopy plus ML—El Khoury et al, 2022	235 serum samples: 60 NMOSD, 60 RRMS, 80 healthy controls, and 35 peripheral-neuropathy controls	Random-forest classification of FTIR spectra; repeated cross-validation plus a small isolated validation set	NMOSD cross-validation AUC 98.7% ± 0.9%; held-out four-class classification was reported as perfect	Proof of concept only: small disease subgroups, no independent external cohort, and spectrometer/site dependence limit generalisability; very high internal performance should not be interpreted as clinical readiness
Ultra-widefield fundus multimodal AI—Gu et al, 2025	1618 UWF images from 1055 participants; subject-ID grouped	Multimodal model combining UWF images with clinical reports; single-centre train/validation/test split with cross-validation	Multimodal test AUC 0.9923; sensitivity 97.0%, specificity 96.9%; UWF-only AUC 0.9751	High internal performance is promising, but the single-centre Chinese cohort, restricted case spectrum, and lack of external validation constrain transportability.

*AUC* area under the receiver–operating characteristic curve, *AQP4-IgG* aquaporin-4 immunoglobulin G, *CI* confidence interval, *FTIR* Fourier-transform infrared spectroscopy, *ML* machine learning, *MOGAD* myelin oligodendrocyte glycoprotein antibody-associated disease, *MRI* magnetic resonance imaging, *MS* multiple sclerosis, *NMOSD* neuromyelitis optica spectrum disorder, *RRMS* relapsing-remitting multiple sclerosis, *UWF* ultra-widefield.

Performance estimates and validation details are reported as described in the cited studies. NR indicates not reported. The final column provides a narrative, design-based appraisal and should not be interpreted as a formal risk-of-bias assessment.

**Table 5:** Representative AI studies in autoimmune encephalitis: design, validation, performance, and methodological limitations.

Application and key study	Cohort and unit of analysis	Design and validation	Performance and comparator	Key limitation and interpretation
MRI differentiation of AE and HSVE—Xiang et al, 2022b	160 AE, 177 HSVE, and 184 healthy controls; 52 additional patients from another site for external validation	3D ResNet18 using multiparametric MRI; internal testing, external-centre validation, and radiologist comparison	Fusion-model external AUC 0.831 for AE and 0.882 for HSVE; AE identification accuracy 83% versus 72% mean radiologist accuracy	External validation and reader comparison strengthen evidence, but the confirmed-case/healthy-control design may introduce spectrum bias; performance in consecutive diagnostically uncertain encephalopathy remains unknown
MRI-negative anti-NMDAR encephalitis detection—Shen et al, 2024	Training: 109 anti-NMDAR encephalitis, 108 healthy controls, and 84 viral encephalitis; validation: 29, 28, and 26, respectively	SVM using quantitative cortical and subcortical MRI features; temporal validation without external-centre testing	Validation AUC 0.879 for anti-NMDAR encephalitis versus viral encephalitis and 0.963 versus healthy controls	The clinically relevant viral-encephalitis comparison showed lower performance than the healthy-control task; lack of external-centre validation and potential scanner/demographic confounding limit transportability
Infectious-versus-autoimmune encephalitis—Petrosian et al, 2025	233 patients: 83 autoimmune/paraneoplastic and 150 infectious encephalitis cases; patient-level analysis	Random-forest model with feature selection; held-out testing and five-fold cross-validation; no external validation	Test AUC 0.966, sensitivity 0.920, specificity 1.000; clinician-comparator AUCs 0.769–0.896	Strong internal performance and clinically relevant comparators, but the retrospective single-centre cohort, class imbalance, limited seronegative AE representation, and absence of external validation constrain generalisability
Clinical–DL–radiomics prognosis—Xiang et al, 2022a	Two-centre anti-NMDAR encephalitis cohort: 139 patients for development/internal testing plus 87 external patients	Multimodal clinical, DL, and hippocampal-radiomics model with independent external-centre testing	Fusion-model AUC 0.963 internally and 0.927 (95% CI 0.688–0.975) externally; external sensitivity 0.667 and specificity 0.947	External testing is a strength, but geographic concentration, manual segmentation, limited calibration reporting, and unmodelled treatment exposure restrict transportability
Long-term cognitive-outcome prediction—Zhang et al, 2025	341 adults from three tertiary hospitals; 264 development and 77 external-validation patients	Six ML algorithms; random forest selected for long-term cognitive-impairment prediction	AUC 0.90; validation accuracy 0.87, sensitivity 0.79, specificity 0.90	Multicentre external validation strengthens evidence, but retrospective cohort construction, telephone-based cognitive assessment, and a relatively small validation cohort limit clinical translation
LLM-assisted diagnosis—Meng et al, 2025	100 antibody-positive AE patients from one centre	DeepSeek-V3 evaluated using stepwise de-identified clinical, MRI, EEG, and CSF information; retrospective proof-of-concept	AE was the most likely diagnosis in 49% and appeared within the leading diagnosis/differential in 65% of cases	Early disease-specific LLM proof of concept only; confirmed antibody-positive cases without non-AE controls, seronegative AE, external validation, or prospective workflow evaluation do not establish diagnostic utility

*AE* autoimmune encephalitis, *AUC* area under the receiver–operating characteristic curve, *CI* confidence interval, *CSF* cerebrospinal fluid, *DL* deep learning, *EEG* electroencephalography, *HSVE* herpes simplex virus encephalitis, *LLM* large language model, *ML* machine learning, *MRI* magnetic resonance imaging, *NMDAR* N-methyl-D-aspartate receptor, *SVM* support vector machine.

Performance estimates and validation details are reported as described in the cited studies. NR indicates not reported. The final column provides a narrative, design-based appraisal and should not be interpreted as a formal risk-of-bias assessment.

**Table 6:** Representative AI studies in myelin oligodendrocyte glycoprotein antibody-associated disease: design, validation, performance, and methodological limitations.

Application and key study	Cohort and unit of analysis	Design and validation	Performance and comparator	Key limitations and interpretation
Three-disease MRI classification—Huang et al, 2022	290 patients: 67 MS, 162 AQP4-IgG-positive NMOSD, and 61 MOGAD; patient-level analysis	MIL-CoaT Transformer using brain and/or spinal-cord MRI; retrospective single-centre development and held-out testing	MOGAD AUC 0.803 (95% CI 0.629–0.949); overall multiclass accuracy 81.4%, versus 64.4% and 74.6% for two neuroradiologists	MOGAD discrimination was lower than for MS or NMOSD, highlighting phenotype overlap; single-centre development and absence of geographically external validation limit transportability
MOGAD-versus-NMOSD radiomics—Luo et al, 2024	212 brain MRI scans from patients with MS, AQP4-IgG-positive NMOSD, and MOGAD; patient-level cross-validation	Multi-lesion radiomics and spatial-distribution modelling using random forest and logistic regression	MOGAD-versus-NMOSD AUC 0.880 ± 0.064; three-disease accuracy 75.0%, versus 47.6–66.0% for three radiologists	Combining lesion-level and spatial information improved discrimination, but retrospective single-centre data, lack of external validation, and restriction to brain MRI limit clinical transportability
Paediatric MOGAD-versus-ADEM radiomics—Ding et al, 2024c	132 children with acute demyelinating syndromes; 66 MOGAD and 66 non-MOGAD, including an external test set of 31 patients	Multi-sequence MRI radiomics with SVM; lesion-level fusion and subject-level modelling; internal and external testing	Subject-level AUC 0.844 internally and 0.846 externally; external sensitivity 93.8% and specificity 66.7%; radiologist accuracy 58.1% externally	External testing strengthens evidence, but the retrospective paediatric cohort, small test sets, strict MRI quality control, and restricted comparator spectrum limit broader applicability
White-matter microstructure classification—Ding et al, 2024b	28 children with MOGAD and 31 age- and sex-matched healthy controls	Automated fibre quantification followed by supervised ML and regression-based classification	All evaluated classifiers achieved AUC > 0.85; logistic regression yielded the best AUC of 0.952	The small healthy-control case–control design is vulnerable to spectrum bias and overfitting; performance does not establish diagnostic utility against clinically relevant inflammatory mimics
Triple-negative optic-neuritis classification — Rechtman et al, 2025	103 patients with first optic neuritis, including MS, MOGAD, NMOSD, and 39 triple-negative cases	Retrospective SVM analysis of onset features to characterise triple-negative optic-neuritis subgroups	Most evolving-MS triple-negative cases clustered with MS, whereas idiopathic cases aligned more closely with MOGAD; quantitative discrimination metrics NR	The model addresses a clinically difficult seronegative subgroup, but small subgroup sizes, uncertain labels, lack of external validation, and absence of quantitative performance estimates preclude clinical use

*ADEM* acute disseminated encephalomyelitis, *AUC* area under the receiver–operating characteristic curve, *AQP4-IgG* aquaporin-4 immunoglobulin G, *CI* confidence interval, *ML* machine learning, *MOGAD* myelin oligodendrocyte glycoprotein antibody-associated disease, *MRI* magnetic resonance imaging, *MS* multiple sclerosis, *NMOSD* neuromyelitis optica spectrum disorder, *NR* not reported, *SVM* support vector machine.

Performance estimates and validation details are reported as described in the cited studies. NR indicates not reported. The final column provides a narrative, design-based appraisal and should not be interpreted as a formal risk-of-bias assessment.

## Clinical decision points for AI support in CNS autoimmune diseases

Clinically useful AI can be organised around four unresolved decision points: earlier recognition and clinical triage; differential diagnosis, disease classification, and subtype recognition; longitudinal risk stratification; and the distinction between risk-informed care planning and treatment-effect estimation. This section defines these clinical decision points and their evaluation requirements; disease-specific empirical evidence is summarised in “Disease-specific evidence and translational gaps”, while forward-looking agentic and knowledge-augmented systems are considered separately in “Forward-looking agentic and knowledge-augmented decision support”.

### Earlier recognition and clinical triage

Early-recognition models should identify patients who warrant expedited investigation or specialist review rather than replace the final clinical diagnosis. Because relevant evidence may be distributed across imaging, serum and CSF biomarkers, electrophysiology, and clinical records, useful systems must integrate available modalities while remaining robust to incomplete data (Bagherieh et al, 2023; Dubey et al, 2018). Evaluation should include clinically representative patients presenting with diagnostic uncertainty and a realistic spectrum of infectious, inflammatory, neoplastic, and non-inflammatory mimics, rather than only well-characterised cases and healthy controls (Nabizadeh et al, 2023; Collins et al, 2024). Models should be assessed at clinically relevant thresholds, with attention to false-negative errors, calibration, time to diagnosis, and incremental value over routine assessment. Outputs should remain reviewable and linked to the patient-level evidence supporting each alert.

### Differential diagnosis, disease classification, and subtype recognition

Classification models address three related tasks: distinguishing CNS autoimmune diseases from infectious, neoplastic, and non-inflammatory mimics; separating overlapping disorders such as MS, NMOSD, and MOGAD; and identifying clinically relevant subgroups within an established diagnosis (Luo et al, 2024; Sun et al, 2025a; Ganjgahi et al, 2025). The reference labels, comparator spectrum, and intended clinical setting should be specified separately for each task. Data-driven clusters or antibody-ambiguous states should not be interpreted as biological subtypes unless they demonstrate stability across cohorts, clinical plausibility, and reproducible associations with longitudinal outcomes. Any claim that a subtype modifies treatment benefit requires separate causal or interventional evaluation.

### Prognostic prediction and longitudinal risk stratification

Prognostic models estimate future relapse, disability, MRI activity, cognitive outcome, critical-care need, or recovery from information available at a defined clinical index point (Seccia et al, 2021; De Brouwer et al, 2021; Andorra et al, 2024; Guo et al, 2025). Each study should prespecify the prediction origin, outcome definition, and prediction horizon, and should align longitudinal imaging, biomarkers, treatment exposure, and clinical assessments accordingly. Information recorded after the predicted outcome must be excluded to prevent temporal leakage, while treatment exposure should be modelled because it alters the subsequent clinical trajectory. Performance should be evaluated over clinically relevant time horizons with calibration and temporal or external validation. Predicted risk may inform monitoring and follow-up planning, but does not establish the benefit of a particular treatment.

### Risk-informed care planning and treatment-effect estimation

Most available models estimate prognosis under observed care rather than the comparative effects of alternative treatments. Risk stratification may inform monitoring and care planning, but high predicted risk does not establish that a particular therapy will improve an individual patient’s outcome. Prognostic models should therefore not be interpreted as validated tools for treatment selection.

Treatment-effect estimation addresses the counterfactual outcomes expected under alternative interventions. This task generally requires causal-inference methods, which are treated in this Review as complementary or AI-adjacent approaches rather than as AI per se. Analyses of observational data require an explicit causal estimand and methods such as target-trial emulation or causal forests, with appropriate handling of confounding by indication, treatment timing, and time-varying exposure (Hernán and Robins, 2016; Wager and Athey, 2018). Prospective interventional evaluation remains necessary before model outputs can support individualised treatment selection.

## Computational molecular discovery and translational research in CNS autoimmunity

Clinical decision support and molecular discovery are linked through patient stratification. Molecular subtypes become clinically actionable only when they are reproducibly associated with outcomes and can be identified using scalable modalities such as serum biomarkers, MRI, OCT, or routinely collected clinical data. Conversely, clinically defined high-risk groups may guide molecular analyses towards mechanisms relevant to prognosis or treatment response. This translational loop remains incomplete: most reviewed studies report molecular associations or generate mechanistic hypotheses rather than demonstrate non-invasive prediction and prospective clinical utility.

### Evidence ladder for molecular discovery

To avoid equating computational nomination with therapeutic validation, we use a molecular evidence ladder. This molecular evidence ladder is distinct from the cross-application evidence-stage mapping in Table 2: it describes the type of translational inference supported by molecular research rather than overall study quality or clinical readiness. The ladder comprises six levels: statistical association, causal prioritisation, mechanistic hypothesis generation, experimental validation, druggability and compound prioritisation, and demonstrated therapeutic efficacy. Level 1—Statistical association. Transcriptomic classifiers, mass-cytometry profiling, and integrated metabolomic analyses identify molecular features associated with disease stage, severity, or patient subgroup. Examples include stage-specific blood-transcriptome signatures, memory B-cell expansion, and reduced hippurate in MS (Acquaviva et al, 2020; Heming et al, 2022; Zhou et al, 2024). NPM1 identified through ML-based multi-omics analysis in AE should similarly be regarded primarily as an associated predictor, with its mechanistic interpretation remaining hypothesis-generating (Guo and Zou, 2026).

Level 2—Causal prioritisation. Mendelian randomisation and Bayesian colocalisation provide statistical evidence that can strengthen target prioritisation but do not establish a biological mechanism or therapeutic efficacy. EVI5, OGA, TNFRSF14, AGER, CD58, and TNF are supported at this level in MS (Yang et al, 2025). FCRL3 also receives causal-prioritisation support and in-silico tractability annotation, but has not thereby reached experimental or clinical validation (Yu et al, 2024).

Level 3—Mechanistic hypothesis generation. Single-cell, spatial, and ligand–receptor analyses nominate cell-specific pathways and interaction networks. KLF/SP transcriptional programmes, the transferrin–LRP2 axis, FGF signalling, and CXCL12–CXCR4 communication are best interpreted as mechanistic hypotheses requiring perturbational confirmation (Elkjaer et al, 2024; Yang et al, 2025).

Level 4—Experimental validation. This level requires direct perturbation in disease-relevant cells, tissues, organoids, or animal models, with demonstration that modifying the candidate changes the proposed biological phenotype. Such validation was not established for most targets discussed above.

Level 5—Druggability and compound prioritisation. Virtual screening, structure-based modelling, and computational drug-repositioning pipelines may identify potentially tractable targets or candidate compounds. Such evidence does not establish target engagement, safety, efficacy, or benefit in a new indication (Elmir et al, 2025; Villar-Delfino et al, 2025).

Level 6—Demonstrated therapeutic efficacy. This level requires evidence that an intervention targeting the nominated mechanism improves a disease-relevant outcome in appropriate experimental systems and, ultimately, in prospective clinical evaluation. No candidate discussed in this section has demonstrated therapeutic efficacy in CNS autoimmune disease solely on the basis of the computational evidence reviewed here.

### From molecular stratification to clinical translation

Closing the molecular-to-clinical loop requires three linked steps: reproducible identification of a molecular subtype, prediction of that subtype using an accessible clinical or imaging surrogate, and prospective demonstration that the resulting stratification improves prognosis assessment or treatment-effect estimation. None of the reviewed CNS autoimmune disease studies has yet completed this full sequence. Molecular and clinical modelling should therefore be viewed as complementary but currently incompletely connected research streams.

### Computational support for clinical trial design and translation

Trial-enrichment models, surrogate endpoints, and federated infrastructure may facilitate evaluation of candidate mechanisms and therapies, but they do not themselves validate an upstream molecular target or establish treatment efficacy.

Translation from target nomination to therapy depends on tractable clinical trials, and CNS autoimmune diseases face well-known design constraints: low prevalence in NMOSD, AE, and MOGAD; phenotypic heterogeneity in MS; and slowly accruing relapse endpoints across all four conditions. AI may help address these constraints through candidate enrichment strategies, quantitative outcome modelling, and multicentre computational infrastructure.

Predictive enrichment uses prespecified prognostic or predictive characteristics to identify trial populations with higher expected event rates or greater potential for treatment-effect heterogeneity, with the aim of improving statistical efficiency. In MS, transcriptomic disease-stage classifiers and treatment-response models illustrate how computational phenotypes could be evaluated as candidate enrichment variables in future trials (Acquaviva et al, 2020; Falet et al, 2022). However, prognostic or treatment-response prediction alone does not establish individual treatment benefit or validate an enrichment strategy. In NMOSD, a multicentre deep-survival model improved relapse-risk prediction over Cox modelling in AQP4-IgG-positive disease, illustrating the potential value of multivariable prognostic stratification for trial planning (Wang et al, 2022). Treatment-related predictors within such models should not, however, be interpreted as evidence of treatment-effect heterogeneity. In AE, prognostic models for anti-NMDAR encephalitis and other clinically defined cohorts illustrate the feasibility of computational risk stratification, but their outputs have not been validated as surrogate endpoints or as tools for adaptive treatment allocation (Xiang et al, 2022a; Li et al, 2026).

AI-derived imaging and digital phenotypes may provide scalable, lower-burden candidate outcome measures for clinical trials. In MS, quantitative MRI composites such as COMRISv2 correlate with cognitive and physical disability and may support further evaluation as trial endpoints, but correlation with clinical outcomes does not by itself establish surrogacy (Kelly et al, 2022). Wearable- and voice-derived measures could similarly support remote longitudinal outcome collection, although their reproducibility and clinical relevance require prospective validation (Hossain et al, 2022; Xia et al, 2025; Delgado Hernández et al, 2026). Multicentre collaboration and federated infrastructure may support adequately powered studies in rare CNS autoimmune diseases, although the disease-specific utility of federated approaches remains to be established prospectively.

## Disease-specific evidence and translational gaps

This section maps disease-specific empirical evidence and priority gaps across MS, NMOSD, AE, and MOGAD. General methodological requirements and sources of bias are discussed in “Data sources and AI methods” and “Clinical decision points for AI support in CNS autoimmune diseases”.

### Multiple sclerosis

#### Evidence profile and dominant tasks

Published AI evidence in MS is concentrated in MRI lesion analysis, disease-activity detection, volumetric monitoring, and disability-risk prediction (Nabizadeh et al, 2023; Bonacchi et al, 2022; Coronado et al, 2021; Bouman et al, 2023). Selected segmentation and disease-monitoring tools have undergone external, multicentre, or prospective workflow evaluation, although evidence that their use improves treatment decisions or patient outcomes remains limited (Wiltgen et al, 2024; Barnett et al, 2023). The revised McDonald criteria recognise the central vein sign and paramagnetic rim lesions as supportive MRI biomarkers in defined diagnostic settings, creating clinically relevant targets for automated assessment (Montalban et al, 2025). Initial multicentre studies demonstrate technical feasibility, but performance, specialised acquisition requirements, and reader-dependent reference labels currently limit routine implementation (Chen et al, 2024; Manning et al, 2025). Evidence beyond MRI, including wearable- and voice-based monitoring, remains early empirical work (Xia et al, 2025; Delgado Hernández et al, 2026). Representative studies are summarised in Table 3, with detailed study characteristics and extraction provided in Table EV2.

#### Priority gaps

Three MS-specific gaps remain. First, available treatment-related models are insufficient for comparative treatment-effect estimation or individual sequencing of disease-modifying therapies (Greselin et al, 2025; Ferrè et al, 2023; Hecker et al, 2024). Second, longitudinal models rarely integrate relapse-independent progression, serial MRI activity, treatment switching, and dynamic disability over multiple years (De Brouwer et al, 2021). Third, selected imaging tools have undergone multicentre or prospective workflow evaluation, but evidence that their use changes clinical decisions or patient outcomes remains limited; non-MRI digital monitoring also requires broader validation (Wiltgen et al, 2024; Barnett et al, 2023; Xia et al, 2025; Delgado Hernández et al, 2026).

### Neuromyelitis optica spectrum disorder

#### Evidence profile and dominant tasks

NMOSD evidence is concentrated in MRI-based differentiation from MS and related demyelinating disorders, with smaller bodies of work on antibody-negative or diagnostically uncertain presentations, spinal-cord lesions, relapse prediction, and non-MRI adjuncts (Kim et al, 2020; Hagiwara et al, 2021). A meta-analysis of MRI-based AI studies reported pooled accuracy, sensitivity, and specificity of ~82%, 83%, and 80%, respectively, for NMOSD-versus-MS discrimination (Etemadifar et al, 2024). Selected studies have used geographically external validation or multicentre prognostic cohorts, including a 10-centre relapse-prediction study (Wang et al, 2022). However, many cohorts emphasise established AQP4-IgG-positive disease and typical MS comparators, with limited evidence for seronegative, overlap, and diagnostically uncertain populations (Cacciaguerra et al, 2022; Shariff et al, 2025). Coexisting Sjögren disease or systemic lupus erythematosus may further complicate phenotyping in AQP4-IgG-positive NMOSD (Moseley et al, 2026; Attfield et al, 2026). Integration of systemic-autoimmune history, rheumatological serology, imaging, and longitudinal electronic health records is therefore an underexplored multimodal application whose clinical utility remains unvalidated. Spectroscopy and ophthalmic-imaging applications remain early empirical work. Representative studies are summarised in Table 4, with detailed study characteristics and extraction provided in Table EV3.

#### Priority gaps

Four NMOSD-specific gaps remain. Individual treatment-effect estimation is undeveloped, with no validated model identified for selecting or sequencing relapse-prevention therapies. Prognostic studies remain concentrated on relapse occurrence rather than attack severity, incomplete recovery, visual outcome, spinal-cord injury, or cumulative disability (Wang et al, 2022). Longitudinal OCT and brain or spinal cord imaging are also underused for monitoring subclinical injury and recovery. Future cohorts should deliberately represent seronegative, antibody-overlap, and systemic-autoimmune-associated NMOSD and report assay methods, ancestry, coexisting autoimmunity, and diagnostic uncertainty (He et al, 2022; Cacciaguerra et al, 2022).

### Autoimmune encephalitis

#### Evidence profile and dominant tasks

AE studies address diagnostic classification, critical-care risk, and longer-term cognitive or functional outcomes. Imaging models have been evaluated for differentiation from infectious encephalitis, early anti-NMDAR encephalitis recognition, and autoantibody-status prediction (Xiang et al, 2022b; Shen et al, 2024; Stake et al, 2024). Clinical models have focused on antibody positivity, ICU admission, severe disease, and prognosis (Xiang et al, 2022a; Wu et al, 2022; Xie et al, 2025; Zhang et al, 2025). Evidence remains geographically concentrated and heterogeneous in antibody subtype, serostatus, treatment timing, and outcome definition. Multi-omics studies remain hypothesis-generating, while an early single-centre LLM proof-of-concept lacks prospective or external validation (Guo and Zou, 2026; Meng et al, 2025). Representative studies are summarised in Table 5, with detailed study characteristics and extraction provided in Table EV4.

#### Priority gaps

Three AE-specific priorities remain. Multicentre cohorts should improve geographical representation and stratify antibody subtype and serostatus rather than pooling clinically distinct syndromes; seronegative AE requires transparent diagnostic adjudication (Graus et al, 2016). EEG-based and multimodal models remain underdeveloped, and their incremental value when structural imaging is unrevealing has not been established. Finally, no validated model estimates the comparative benefit of second-line immunotherapy after an inadequate first-line response, making disease-specific treatment-effect estimation a priority.

### Myelin oligodendrocyte glycoprotein antibody-associated disease

#### Evidence profile and dominant tasks

The 2023 international diagnostic criteria for MOGAD were proposed by Banwell et al (2023) and subsequently validated in a selected adult and paediatric cohort by Varley et al (2024). Current studies are concentrated in relapse-risk prediction, differentiation from overlapping demyelinating disorders, paediatric imaging, and optic-neuritis assessment. The MOG-AR score was developed and internally validated in a registry-based multicentre Chinese cohort (Xu et al, 2024). However, external validation in a 284-patient UK cohort showed only modest discrimination for three-year relapse risk, with an AUROC of 0.67 (95% CI 0.61–0.73) for the continuous score and 0.58 (95% CI 0.52–0.63) for the corresponding risk grade, together with overestimation of relapse probabilities (Yeh et al, 2026). MRI and OCT studies have addressed cross-disease differentiation and structural injury (Luo et al, 2024; Ding et al, 2024c; Pakeerathan et al, 2026). Takai et al described a destructive histopathological pattern in severe disease, characterised by prominent C9neo deposition and oligodendrocyte loss; however, the relationship of this pattern to clinical recovery remains unvalidated and hypothesis-generating (Takai et al, 2026). Although many patients with MOGAD recover substantially after an acute attack, a clinically important subset develops incomplete or severe residual disability. Early prediction of recovery trajectory from a prespecified disease-onset or attack-level index time therefore represents an important unmet need. Future multimodal models integrating onset phenotype, MRI or OCT features, MOG-IgG status or titre, treatment exposure, and longitudinal outcomes could help identify patients at higher risk of poor recovery and support risk-informed follow-up and rehabilitation planning. Such models should not, however, be interpreted as validated tools for individual treatment selection. Recovery-oriented modelling remains underdeveloped relative to relapse prediction. Representative AI studies are summarised in Table 6, with clinically relevant AI-adjacent evidence and detailed study extraction provided in Table EV5.

#### Priority gaps

Four MOGAD-specific gaps remain. First, adult-specific models are limited, and mixed-age cohorts may obscure age-dependent phenotype, relapse, and recovery patterns. Second, comparative treatment-effect estimation for maintenance therapy is absent; existing relapse-risk models have shown limited cross-cohort transportability and should not be interpreted as validated tools for treatment selection. Third, prognostic studies remain focused primarily on monophasic versus relapsing disease, with limited attention to early prediction of recovery magnitude, severe residual disability, visual and functional outcomes, and rehabilitation needs after an index attack. Future studies should define the prediction origin and outcome horizon in advance and evaluate whether clinical, imaging, serological, and treatment-related features improve prediction beyond established clinical variables. Finally, serial MOG-IgG status or titre has rarely been incorporated into longitudinal models, and its incremental value beyond clinical and imaging features remains uncertain. Multicentre studies should address these questions using prespecified longitudinal outcomes, age-stratified analyses, and independent external validation (Xu et al, 2024; Yeh et al, 2026; Cheng et al, 2025).

### Cross-disease mapping of evidence patterns and priority gaps

Across MS, NMOSD, AE, and MOGAD, the literature differs in scale, dominant application, validation setting, and unresolved clinical need. Imaging-based monitoring is most extensively represented in MS, whereas studies in NMOSD, AE, and MOGAD are generally smaller and more heterogeneous. Table 7 provides a descriptive mapping rather than a formal ranking of evidence quality or clinical readiness.

**Table 7:** Cross-disease mapping of evidence patterns, validation settings, and priority gaps.

Disease	Evidence distribution	Dominant application	Reported validation setting	Notable evidence feature	Priority unmet need
MS	Evidence is concentrated in MRI analysis and longitudinal disease monitoring, with additional studies addressing prognostic and specialised imaging applications.	Lesion segmentation and volumetry; longitudinal disease-activity monitoring; emerging automated CVS/PRL assessment	Includes external benchmarks, multicentre studies, and selected prospective workflow evaluations	Large imaging cohorts, external benchmarks, and selected prospective workflow evaluations of automated longitudinal MRI assessment	Comparative treatment-effect estimation; multi-year progression modelling; evidence that AI-assisted workflows improve clinical decisions or patient outcomes
NMOSD	Evidence is concentrated in differential diagnosis and relapse-risk prediction.	Differentiation from MS and MOGAD; seronegative-disease support; relapse-risk modelling	Selected geographically external and multicentre validations, but limited representation of seronegative, overlap, and diagnostically uncertain populations	MRI-dominant evidence with selected serum-spectroscopy and ophthalmic/fundus applications	Representative seronegative and systemic-autoimmune-overlap cohorts; recovery prediction; comparative treatment-effect estimation; longitudinal OCT and spinal-cord monitoring
AE	Geographically concentrated evidence with heterogeneous antibody subtypes, serostatus, and outcome definitions	Diagnostic classification and prognostic modelling, including cognitive and functional outcomes	Predominantly retrospective studies, with selected external-centre or multicentre validation	Emerging multimodal imaging and disease-specific LLM studies; post-hoc attribution is frequently reported but rarely independently validated	EEG-based multimodal modelling; antibody-subtype and seronegative cohorts; prospective validation; comparative treatment-effect estimation for second-line immunotherapy
MOGAD	Evidence is concentrated in cross-disease and paediatric imaging applications, with limited disease-specific prognostic AI studies.	Cross-disease MRI classification; paediatric radiomics and microstructural imaging	Selected external paediatric imaging validation; limited prospective disease-specific AI evaluation	Cross-disease imaging and paediatric modelling predominate; clinically relevant relapse-score and OCT evidence remains largely AI-adjacent	Adult-specific models; early recovery and residual-disability prediction; comparative treatment-effect estimation; longitudinal integration of MOG-IgG status or titre

*AE* autoimmune encephalitis, *AI* artificial intelligence, *CVS* central vein sign, *EEG* electroencephalography, *LLM* large language model, *MOGAD* myelin oligodendrocyte glycoprotein antibody-associated disease, *MRI* magnetic resonance imaging, *MS* multiple sclerosis, *NMOSD* neuromyelitis optica spectrum disorder, *OCT* optical coherence tomography, *PRL* paramagnetic rim lesion.

This table provides a qualitative cross-disease mapping of evidence patterns identified in this Review and should not be interpreted as a quantitative ranking of research maturity, methodological quality, or clinical readiness. Validation settings indicate the strongest type of disease-specific evidence identified, while individual studies may remain subject to substantial risk of bias.

#### Evidence-base biases

AE studies are geographically concentrated, MOGAD imaging cohorts include substantial paediatric representation, MS evidence frequently derives from European or North American imaging cohorts, and NMOSD studies often emphasise established AQP4-IgG-positive disease and typical MS comparators. Across diseases, specialist-centre recruitment may enrich severe or diagnostically resolved cases and limit applicability to unselected populations.

#### Three cross-disease patterns emerge

**Fig. 2:**
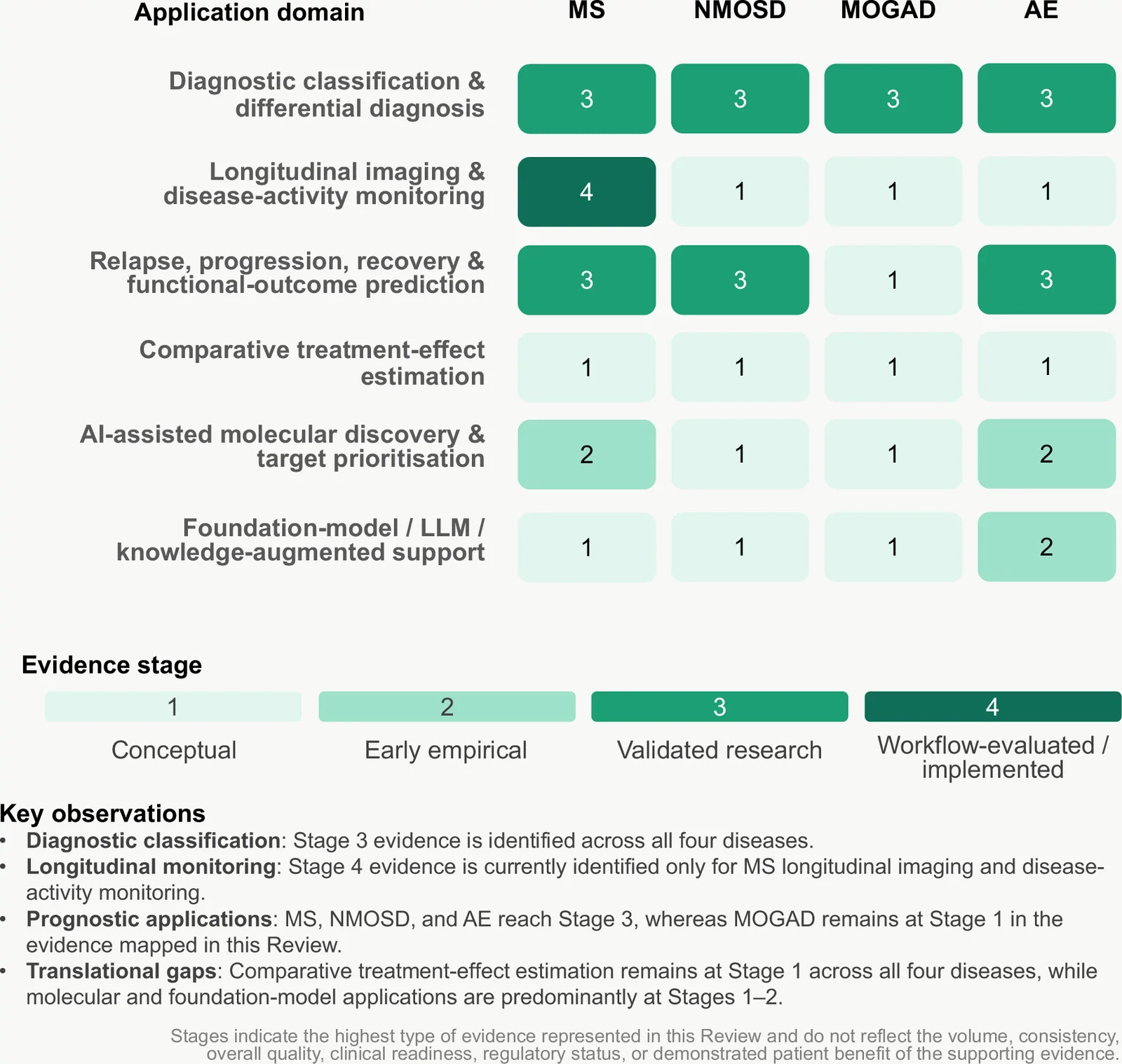
Qualitative evidence-stage mapping of AI applications across CNS autoimmune diseases. Qualitative map of the strongest evidence stage identified for AI applications across six clinical task domains and four diseases (multiple sclerosis (MS), neuromyelitis optica spectrum disorder (NMOSD), myelin oligodendrocyte glycoprotein antibody-associated disease (MOGAD), and autoimmune encephalitis (AE)). Evidence stages are: Stage 1, conceptual; Stage 2, early empirical; Stage 3, validated research; and Stage 4, workflow-evaluated or implemented. Each cell reflects the highest stage identified for the disease–application combination in this Review and does not represent the volume, consistency, overall quality, regulatory status, or clinical benefit of the supporting evidence. The figure should therefore be interpreted as an orienting evidence map rather than a formal maturity grade.

Diagnostic and prognostic applications outnumber comparative treatment-effect studies, with no prospectively validated model identified for individual treatment selection (Fig. 2).  Validation patterns remain disease-specific, ranging from prospective MS imaging workflows and international MOGAD cohorts to underrepresentation of seronegative NMOSD and geographically concentrated AE evidence. EEG in AE and longitudinal OCT in optic-neuritis-associated disorders also remain less developed than conventional MRI applications.

## Conclusions and future directions

Building on the evidence map above, this section outlines priorities for research infrastructure, forward-looking model classes, equitable evaluation, benchmarking, clinical-impact assessment, and implementation. General limitations of study design, validation, and reporting are discussed in “Methodological quality, sources of bias, and reporting standards” and are not repeated here.

### Multicentre and federated infrastructure for rare CNS autoimmune diseases

Multicentre consortia should use common data elements, harmonised annotations, versioned diagnostic criteria, scanner and assay metadata, treatment exposure, and longitudinal outcomes. Federated learning may support collaboration when raw-data pooling is restricted, but should be evaluated against centralised or site-specific baselines and for between-centre heterogeneity, privacy, and operational burden. Existing MS studies establish research-level feasibility, whereas disease-specific evaluation in NMOSD, AE, and MOGAD remains a priority (Bai et al, 2024; Pirmani et al, 2025; Hindawi et al, 2025).

### Forward-looking agentic and knowledge-augmented decision support

Agentic, retrieval-augmented, and ontology-guided systems could link patient-level findings with versioned diagnostic criteria, guidelines, literature, and disease ontologies. In the adjacent field of rare-disease diagnosis, DeepRare integrates heterogeneous clinical inputs and up-to-date medical knowledge sources to generate ranked diagnostic hypotheses with evidence-linked, traceable reasoning (Zhao et al, 2026). However, this study does not constitute disease-specific clinical validation in CNS autoimmune diseases. Their near-term role should therefore be source-grounded evidence retrieval and hypothesis prioritisation rather than autonomous diagnosis or treatment selection. Outputs should remain traceable to supporting sources, version-controlled, reviewable by clinicians, and subject to preserved clinical accountability.

### Forward-looking foundation models and medical large language models

Foundation models learn reusable representations through large-scale pre-training before downstream adaptation, potentially reducing—but not eliminating—the need for large disease-specific labelled datasets (Schäfer et al, 2024; Sun et al, 2025b). Multimodal vision–language models could integrate imaging, clinical text, biomarkers, treatment exposure, and longitudinal outcomes in rare disorders (Kim et al, 2024; Zhang et al, 2024). However, most current evidence derives from outside neuroimmunology, with only limited disease-specific proof-of-concept evidence in CNS autoimmune diseases. For example, a single-centre retrospective study evaluated an LLM for antibody-positive autoimmune encephalitis, but lacked external or prospective validation (Meng et al, 2025). Domain shift, rare-disease under-representation, hallucination, poor calibration, and computational burden may limit reliability. Disease-specific benchmarks and external evaluation are required, and no prospective clinical validation of foundation models or medical LLMs in CNS autoimmune diseases was identified.

### Equitable evaluation and demographic representativeness

Evaluation should reflect variation in ancestry, sex, age, geography, and healthcare setting across CNS autoimmune diseases. Cohorts should report demographic composition and prespecified subgroup performance, error rates, and uncertainty analyses, consistent with the trustworthiness framework described in “Trustworthiness and privacy-preserving computation”. Prospective evaluation in diverse settings is required before broad implementation. When representative recruitment is infeasible, studies should limit claims to the evaluated population and use explicit uncertainty or abstention for poorly represented groups.

### Task-specific benchmarks and reproducible evaluation

Cross-study comparison requires task-specific benchmarks with harmonised cohort definitions, reference standards, analysis units, and outcome horizons. Benchmark initiatives should use locked multicentre test sets, patient- and site-level separation, prespecified metrics, and versioned reference labels. Priorities include longitudinal MS lesion analysis, discrimination among overlapping demyelinating disorders, and AE outcome prediction. Where governance and licensing permit, studies should share code, preprocessing specifications, model documentation, and executable evaluation procedures. Hidden-label evaluation platforms may provide an alternative when data or model release is restricted.

### From model performance to clinical-impact evaluation

Task-level performance is necessary but insufficient to establish clinical utility. Evaluation should assess calibration and decision-curve utility where applicable, together with workflow and patient outcomes such as diagnostic delay, clinician workload, treatment timing, relapse, disability, cognition, and quality of life. Staged evaluation may begin with silent prospective deployment to assess real-world performance and failure modes. Tools intended to influence clinical decisions should subsequently undergo interventional evaluation, such as stepped-wedge cluster trials or randomised comparisons with standard care.

### From model to practice: regulatory and implementation barriers

**Fig. 3:**
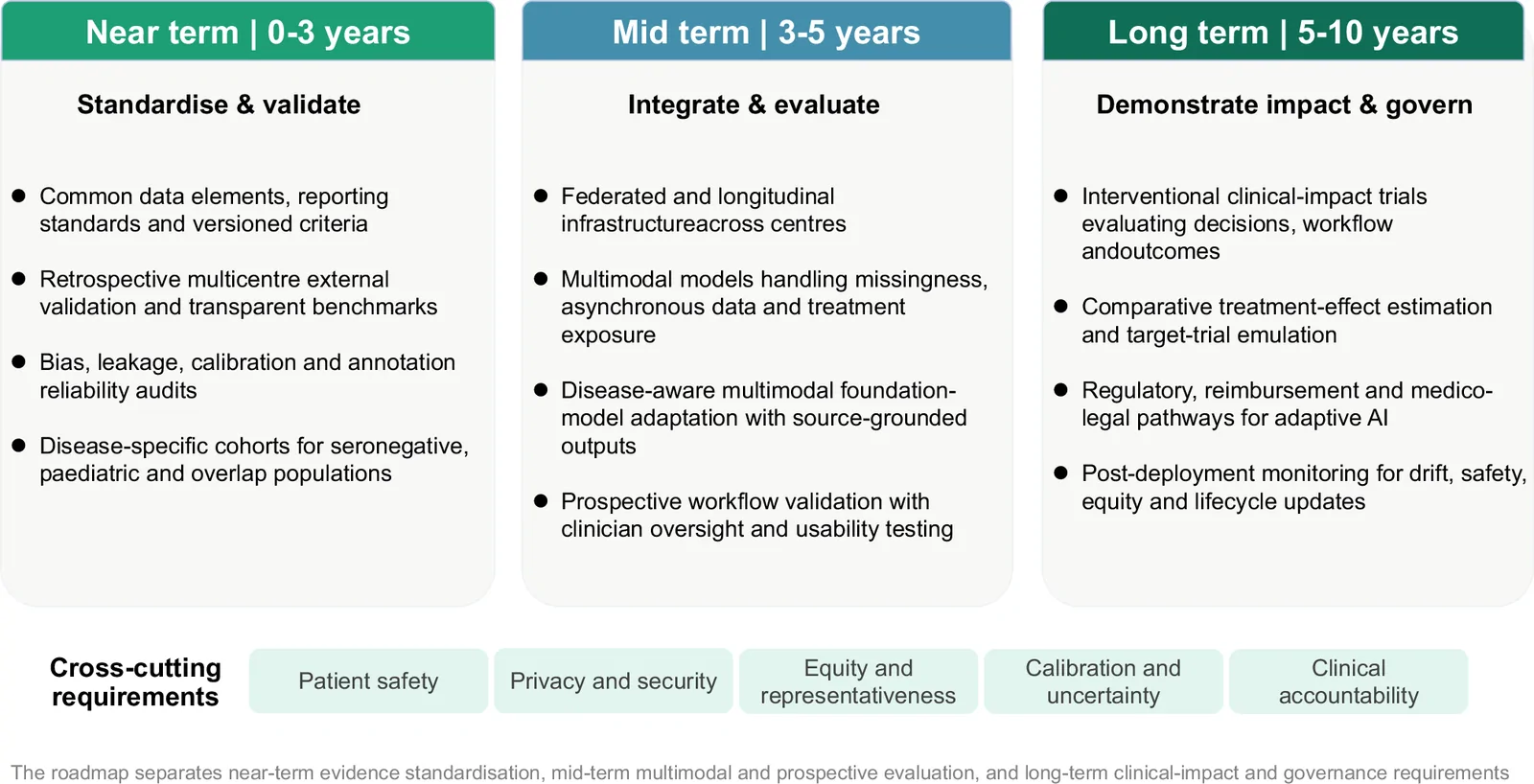
Roadmap for the next 5–10 years of AI research and translation in CNS autoimmune diseases. The roadmap organises future priorities into three time horizons: near-term (0–3 years), mid-term (3–5 years), and long-term (5–10 years). Priorities are grouped into six coordinated workstreams: multicentre data infrastructure, trustworthy evaluation, multimodal integration, foundation models, prospective clinical validation, and regulatory and implementation pathways. Near-term priorities focus on standardised datasets, reporting quality, retrospective multicentre validation, and harmonisation of multimodal data. Mid-term priorities emphasise federated and longitudinal infrastructure, prospective workflow validation, disease-aware multimodal foundation models, and uncertainty-aware benchmarking. Long-term priorities include interventional trials with clinical endpoints, real-world deployment, reimbursement and regulatory integration, and post-market performance surveillance.

Regulatory requirements should be proportionate to intended use and clinical risk. Relevant frameworks include FDA oversight of AI-enabled device software, predetermined change control plans, and medical-device cybersecurity; the EU Medical Device Regulation and AI Act; and risk-based regulation of AI medical software in China (FDA, 2025, 2026; European Parliament and Council, 2017, 2024; NMPA, 2021; CMDE, 2022). Implementation requires local validation across scanner protocols, antibody assays, referral populations, and treatment practices, together with interoperability, clinician training, override and audit procedures, defined liability, and evidence supporting reimbursement or economic value. Post-deployment surveillance should monitor performance, subgroup safety, data and practice drift, cybersecurity, overrides, and adverse consequences. Multicentre governance should prespecify investigation, rollback, recalibration, and regulated updating. Figure 3 summarises the staged research, validation, and implementation roadmap for AI in CNS autoimmune diseases.

Overall, current evidence supports assistive research and selected workflow-evaluated applications, mainly in MS imaging, but not autonomous diagnosis or treatment selection. Near-term value is more plausibly expected from multimodal evidence integration, standardised image and biomarker assessment, risk stratification, molecular hypothesis prioritisation, and the evaluation of candidate trial-enrichment strategies and outcome measures. Translation into routine care will require harmonised multicentre evidence, equitable and clinically meaningful evaluation, and lifecycle governance. Until these conditions are met, claims of direct clinical benefit should remain proportionate to the level of validation achieved.

### Pending issues

- How can multicentre datasets better represent real-world diagnostic uncertainty, including seronegative, overlap, paediatric, and under-represented populations?
- Which validation designs are sufficient to establish transportability across scanners, assays, institutions, populations, and healthcare systems?
- Can longitudinal multimodal models integrate asynchronous and missing data while improving the prediction of relapse, recovery, disability, or cognitive outcomes beyond standard clinical assessment?
- How can prognostic models be extended to estimate comparative treatment effects without being dominated by confounding by indication?
- What prospective and interventional evidence is required to demonstrate improvements in clinical decisions, workflow efficiency, or patient outcomes rather than predictive performance alone?
- How can foundation models, large language models, and agentic systems achieve adequate source traceability, calibration, uncertainty control, privacy protection, and regulatory oversight for clinical use?
- Which computationally prioritised molecular mechanisms and therapeutic targets can withstand causal and experimental validation and ultimately translate into effective therapies?

### For more information

- National Multiple Sclerosis Society—information and resources on multiple sclerosis for patients, clinicians, and researchers. https://www.nationalmssociety.org/.
- The MOG Project—patient, educational, and research resources for MOG antibody-associated disease. https://mogproject.org/.
- Autoimmune Encephalitis Alliance—information, support, and research resources for autoimmune encephalitis. https://aealliance.org/.
- Orphanet—an expert-curated portal for rare-disease nomenclature, classification, and disease information. https://www.orpha.net/.
- ClinicalTrials.gov—a registry and results database for clinical studies worldwide. https://clinicaltrials.gov/.
- EQUATOR Network—reporting guidelines and methodological resources for health research. https://resources.equator-network.org/.
- China National Registry of Neuro-Inflammatory Diseases (CNRID). https://www.ncrcnd.org.cn/cnrid-en/.

## Supplementary information

Table EV1

Table EV2

Table EV3

Table EV4

Table EV5

Peer Review File

## **Glossary**

**Agentic AI**: An AI system designed to perform a sequence of linked tasks, such as retrieving information, evaluating evidence, and generating recommendations, with some degree of autonomous task planning.
**Artificial intelligence (AI)**: A broad term for computer systems designed to perform tasks that normally require human reasoning, pattern recognition, or decision-making.
**Calibration**: The agreement between predicted probabilities and observed outcomes; for example, whether approximately 20% of patients assigned a 20% risk actually experience the event.
**Central vein sign (CVS)**: An MRI feature in which a small vein runs through a white-matter lesion; when assessed in an appropriate clinical context, it can support the diagnosis of multiple sclerosis.
**Clinical workflow evaluation**: Testing an AI system within an actual clinical process to determine not only predictive performance but also effects on usability, clinician decisions, workload, safety, or patient outcomes.
**Data leakage**: Accidental use during model development of information that would not legitimately be available when the model is applied to a new patient, leading to overly optimistic performance estimates.
**Deep learning (DL)**: A form of machine learning that uses multilayer neural networks to learn complex patterns, particularly from images, text, and other high-dimensional data.
**External validation**: Testing a model using patients or data that are independent of those used to develop the model, to assess whether performance generalises to new settings.
**Federated learning**: A method for training a shared model across multiple institutions without transferring the underlying raw patient data to a central location.
**Foundation model**: A large model pretrained on broad datasets that can subsequently be adapted to multiple specific tasks with additional training or prompting.
**Large language model (LLM)**: A foundation model trained primarily on large collections of text to interpret, generate, summarise, and organise language-based information.
**Machine learning (ML)**: A branch of AI in which computer models learn patterns from data rather than relying only on predefined rules.
**Multicentre validation**: Evaluation involving data collected at more than one clinical institution, which can help determine whether a model remains reliable across different patient populations and clinical practices.
**Multimodal AI**: AI that combines different types of information, such as medical images, laboratory results, clinical records, electrophysiology, and molecular data.
**Overfitting**: A situation in which a model learns the peculiarities of its development data too closely and consequently performs less well on new patients.
**Paramagnetic rim lesion (PRL)**: A chronic multiple-sclerosis lesion with an iron-associated rim visible on susceptibility-sensitive MRI, reflecting persistent inflammatory activity at the lesion edge.
**Post-hoc attribution**: A method applied after model training, such as SHAP or Grad-CAM, to indicate which input features contributed to a prediction; it does not by itself prove that the model’s reasoning is clinically valid.
**Radiomics**: Computational extraction of large numbers of quantitative features from medical images, including patterns that may not be apparent by visual inspection alone.
**Retrieval-augmented generation (RAG)**: An approach in which a language model retrieves information from external sources, such as guidelines or literature, before generating an answer.
**Risk prediction**: Estimation of the probability that a future event, such as relapse, disability progression, or poor recovery, will occur under observed clinical care.
**Seronegative disease**: A clinically compatible autoimmune disorder in which the currently tested disease-associated antibody is not detected.
**Spectrum bias**: Distortion of apparent model performance when evaluation includes unusually clear-cut cases, such as confirmed disease versus healthy controls, rather than the full range of patients encountered in clinical practice.
**Treatment-effect estimation**: Estimation of how a patient’s outcome might differ under alternative treatments, rather than simply predicting whether that patient is at high or low risk.
**Uncertainty estimation**: Methods used to quantify how confident a model is in an individual prediction and to identify situations in which the model should abstain or defer to human review.

## References

[CR1] Acosta JN, Falcone GJ, Rajpurkar P, Topol EJ (2022) Multimodal biomedical AI. Nat Med 28:1773–1784 10.1038/s41591-022-01981-2 36109635

[CR2] Acquaviva M, Menon R, Di Dario M, Dalla Costa G, Romeo M, Sangalli F, Colombo B, Moiola L, Martinelli V, Comi G et al (2020) Inferring multiple sclerosis stages from the blood transcriptome via machine learning. Cell Rep Med 1:100053 10.1016/j.xcrm.2020.100053 PMC7659538 33205062

[CR4] Amin M, Martínez-Heras E, Ontaneda D, Prados Carrasco F (2024) Artificial intelligence and multiple sclerosis. Curr Neurol Neurosci Rep 24:233–243 10.1007/s11910-024-01354-x PMC11258192 38940994

[CR5] Andorra M, Freire A, Zubizarreta I, Kerlero de Rosbo N, Bos SD, Rinas M, Høgestøl EA, de Rodez Benavent SA, Berge T, Brune-Ingebretsen S et al (2024) Predicting disease severity in multiple sclerosis using multimodal data and machine learning. J Neurol 271:1133–1149 10.1007/s00415-023-12132-z PMC10896787 38133801

[CR7] Attfield KE, Armen AP, Kuttikkatte SB, Farooq R, Francis AG, Dendrou CA, Leite MI, Waters P, International NMOSD Genetics Consortium, Weinshenker BG et al (2026) Identification of genetic risk loci associated with aquaporin 4-positive neuromyelitis optica spectrum disorder: a genome-wide association study. Lancet Neurol 25:482–491 10.1016/S1474-4422(26)00084-0 42009010

[CR8] Bagherieh S, Afshari-Safavi A, Vaheb S, Kiani M, Ghaffary EM, Barzegar M, Shaygannejad V, Zabeti A, Mirmosayyeb O (2023) Worldwide prevalence of neuromyelitis optica spectrum disorder (NMOSD) and neuromyelitis optica (NMO): a systematic review and meta-analysis. Neurol Sci 44:1905–1915 10.1007/s10072-023-06617-y 36745300

[CR9] Bai L, Wang D, Wang H, Barnett M, Cabezas M, Cai W, Calamante F, Kyle K, Liu D, Ly L et al (2024) Improving multiple sclerosis lesion segmentation across clinical sites: a federated learning approach with noise-resilient training. Artif Intell Med 152:102872 10.1016/j.artmed.2024.102872 38701636

[CR10] Banwell B, Bennett JL, Marignier R, Kim HJ, Brilot F, Flanagan EP, Ramanathan S, Waters P, Tenembaum S, Graves JS et al (2023) Diagnosis of myelin oligodendrocyte glycoprotein antibody-associated disease: International MOGAD Panel Proposed Criteria. Lancet Neurol 22:268–282 10.1016/S1474-4422(22)00431-8 36706773

[CR11] Barnett M, Wang D, Beadnall H, Bischof A, Brunacci D, Butzkueven H, Brown JWL, Cabezas M, Das T, Dugal T et al (2023) A real-world clinical validation for AI-based MRI monitoring in multiple sclerosis. NPJ Digit Med 6:196 10.1038/s41746-023-00940-6 PMC10587188 37857813

[CR13] Bonacchi R, Filippi M, Rocca MA (2022) Role of artificial intelligence in MS clinical practice. Neuroimage Clin 35:103065 10.1016/j.nicl.2022.103065 PMC9163993 35661470

[CR14] Bouman PM, Noteboom S, Nobrega Santos FA, Beck ES, Bliault G, Castellaro M, Calabrese M, Chard DT, Eichinger P, Filippi M et al (2023) Multicenter evaluation of AI-generated DIR and PSIR for cortical and juxtacortical multiple sclerosis lesion detection. Radiology 307:e221425 10.1148/radiol.221425 PMC10102645 36749211

[CR16] Cacciaguerra L, Storelli L, Radaelli M, Mesaros S, Moiola L, Drulovic J, Filippi M, Rocca MA (2022) Application of deep-learning to the seronegative side of the NMO spectrum. J Neurol 269:1546–1556 10.1007/s00415-021-10727-y 34328544

[CR17] Center for Medical Device Evaluation, National Medical Products Administration (2022) Guideline for registration review of artificial intelligence medical devices. CMDE Announcement No. 8 of 2022.

[CR18] Chase HS, Mitrani LR, Lu GG, Fulgieri DJ (2017) Early recognition of multiple sclerosis using natural language processing of the electronic health record. BMC Med Inf Decis Mak 17:24 10.1186/s12911-017-0418-4 PMC5329909 28241760

[CR19] Chen L, Ren Z, Clark KA, Lou C, Liu F, Cao Q, Manning AR, Martin ML, Luskin E, O’Donnell CM et al (2024) Multicenter validation of automated detection of paramagnetic rim lesions on brain MRI in multiple sclerosis. J Neuroimaging 34:750–757 10.1111/jon.13242 PMC12370289 39410780

[CR21] Cheng J, Wang Z, Wang J, Pang X, Wang J, Zhang M, Guo J, Meng H (2025) The nomogram model predicts relapse risk in myelin oligodendrocyte glycoprotein antibody-associated disease: a single-center study. Front Immunol 16:1527057 10.3389/fimmu.2025.1527057 PMC11911489 40098969

[CR22] Collins GS, Moons KGM, Dhiman P, Riley RD, Beam AL, Van Calster B, Ghassemi M, Liu X, Reitsma JB, van Smeden M et al (2024) TRIPOD + AI statement: updated guidance for reporting clinical prediction models that use regression or machine learning methods. BMJ 385:e078378 10.1136/bmj-2023-078378 PMC11019967 38626948

[CR23] Coronado I, Gabr RE, Narayana PA (2021) Deep learning segmentation of gadolinium-enhancing lesions in multiple sclerosis. Mult Scler 27:519–527 10.1177/1352458520921364 PMC7680286 32442043

[CR24] Cruz Rivera S, Liu X, Chan AW, Denniston AK, Calvert MJ, SPIRIT-AI and CONSORT-AI Working Group (2020) Guidelines for clinical trial protocols for interventions involving artificial intelligence: the SPIRIT-AI extension. Lancet Digit Health 2:e549–e560 10.1016/S2589-7500(20)30219-3 PMC8212701 33328049

[CR25] David Cancino CA, Trenado C, Kaplan PW, Gómez Ávila FA, Fernández González-Aragón MDC, Moreno Avellán ÁJ, Soto Rincón CA, Quiñones Pesqueira GA, San-Juan D (2025) Quantitative electroencephalography biomarkers in patients with anti-N-methyl-D-aspartate receptor encephalitis: a case-control study. J Clin Neurophysiol 42:314–322 10.1097/WNP.0000000000001124 39724935

[CR26] De Brouwer E, Becker T, Moreau Y, Havrdova EK, Trojano M, Eichau S, Ozakbas S, Onofrj M, Grammond P, Kuhle J et al (2021) Longitudinal machine learning modeling of MS patient trajectories improves predictions of disability progression. Comput Methods Prog Biomed 208:106180 10.1016/j.cmpb.2021.106180 34146771

[CR27] Delgado Hernández J, Betancort Montesinos M, Romero Arias T, Hernández Pérez MÁ (2026) Voice analysis as a digital biomarker: a machine learning approach for automated multiple sclerosis classification. Mult Scler Relat Disord 109:107109 10.1016/j.msard.2026.107109 41780469

[CR28] Devlin J, Chang MW, Lee K, Toutanova K (2019) BERT: pre-training of deep bidirectional transformers for language understanding. In: Proceedings of the 2019 Conference of the North American Chapter of the Association for Computational Linguistics: Human Language Technologies, 1. Association for Computational Linguistics, Minneapolis, MN, pp 4171–4186

[CR30] Ding S, Shi Z, Huang K, Fan X, Li X, Zheng H, Wang L, Yan Z, Cai J (2024) Aberrant white matter microstructure detected by automatic fiber quantification in pediatric myelin oligodendrocyte glycoprotein antibody-associated disease. Mult Scler Relat Disord 84:105483 10.1016/j.msard.2024.105483 38354445

[CR31] Ding S, Zheng H, Wang L, Fan X, Yang X, Huang Z, Zhang X, Yan Z, Li X, Cai J (2024c) Classification of myelin oligodendrocyte glycoprotein antibody-related disease and its mimicking acute demyelinating syndromes in children using MRI-based radiomics: from lesion to subject. Acad Radiol 31:2085–2096 10.1016/j.acra.2023.11.011 38007367

[CR32] Dosovitskiy A, Beyer L, Kolesnikov A, Weissenborn D, Zhai X, Unterthiner T, Dehghani M, Minderer M, Heigold G, Gelly S et al (2021) An image is worth 16×16 words: transformers for image recognition at scale. In: 9th International Conference on Learning Representations (ICLR 2021). ICLR

[CR33] Dubey D, Pittock SJ, Kelly CR, McKeon A, Lopez-Chiriboga AS, Lennon VA, Gadoth A, Smith CY, Bryant SC, Klein CJ et al (2018) Autoimmune encephalitis epidemiology and a comparison to infectious encephalitis. Ann Neurol 83:166–177 10.1002/ana.25131 PMC6011827 29293273

[CR34] El Khoury Y, Gebelin M, de Sèze J, Patte-Mensah C, Marcou G, Varnek A, Mensah-Nyagan AG, Hellwig P, Collongues N (2022) Rapid discrimination of neuromyelitis optica spectrum disorder and multiple sclerosis using machine learning on infrared spectra of sera. Int J Mol Sci 23:2791 10.3390/ijms23052791 PMC8911153 35269934

[CR35] Elkjaer ML, Hartebrodt A, Oubounyt M, Weber A, Vitved L, Reynolds R, Thomassen M, Rottger R, Baumbach J, Illes Z (2024) Single-cell multi-omics map of cell type-specific mechanistic drivers of multiple sclerosis lesions. Neurol Neuroimmunol Neuroinflamm 11:e200213 10.1212/NXI.0000000000200213 PMC11073880 38564686

[CR36] Elmir H, Ghazli A, Boubchir L, Daoudi A (2025) Machine learning classification of TLR7 ligands for drug discovery in auto-immune diseases. In: 2025 International Conference on Artificial Intelligence and Innovative Applications (AIIA). IEEE, pp 1–8

[CR37] Etemadifar M, Norouzi M, Alaei SA, Karimi R, Salari M (2024) The diagnostic performance of AI-based algorithms to discriminate between NMOSD and MS using MRI features: a systematic review and meta-analysis. Mult Scler Relat Disord 87:105682 10.1016/j.msard.2024.105682 38781885

[CR38] European Parliament and Council of the European Union (2017) Regulation (EU) 2017/745 on medical devices. Official Journal of the European Union L117:1–175.

[CR39] European Parliament and Council of the European Union (2024) Regulation (EU) 2024/1689 laying down harmonised rules on artificial intelligence. Official Journal of the European Union L2024/1689.

[CR40] Falet JPR, Durso-Finley J, Nichyporuk B, Schroeter J, Bovis F, Sormani MP, Precup D, Arbel T, Arnold DL (2022) Estimating individual treatment effect on disability progression in multiple sclerosis using deep learning. Nat Commun 13:5645 10.1038/s41467-022-33269-x PMC9512913 36163349

[CR42] Ferrè L, Clarelli F, Pignolet B, Mascia E, Frasca M, Santoro S, Sorosina M, Bucciarelli F, Moiola L, Martinelli V et al (2023) Combining clinical and genetic data to predict response to fingolimod treatment in relapsing remitting multiple sclerosis patients: a precision medicine approach. J Pers Med 13:122 10.3390/jpm13010122 PMC9861774 36675783

[CR43] Flanagan EP, Geschwind MD, Lopez-Chiriboga AS, Blackburn KM, Turaga S, Binks S, Zitser J, Gelfand JM, Day GS, Dunham SR et al (2023) Autoimmune encephalitis misdiagnosis in adults. JAMA Neurol 80:30–39 10.1001/jamaneurol.2022.4251 PMC9706400 36441519

[CR44] Food and Drug Administration (2025) Marketing submission recommendations for a predetermined change control plan for artificial intelligence-enabled device software functions. US Food and Drug Administration, Silver Spring

[CR45] Food and Drug Administration (2026) Cybersecurity in medical devices: quality management system considerations and content of premarket submissions. US Food and Drug Administration, Silver Spring

[CR46] Fortin JP, Parker D, Tunç B, Watanabe T, Elliott MA, Ruparel K, Roalf DR, Satterthwaite TD, Gur RC, Gur RE et al (2017) Harmonization of multi-site diffusion tensor imaging data. Neuroimage 161:149–170 10.1016/j.neuroimage.2017.08.047 PMC5736019 28826946

[CR47] Gabr RE, Coronado I, Robinson M, Sujit SJ, Datta S, Sun X, Allen WJ, Lublin FD, Wolinsky JS, Narayana PA (2020) Brain and lesion segmentation in multiple sclerosis using fully convolutional neural networks: a large-scale study. Mult Scler 26:1217–1226 10.1177/1352458519856843 PMC6908772 31190607

[CR48] Gaj S, Ontaneda D, Nakamura K (2021) Automatic segmentation of gadolinium-enhancing lesions in multiple sclerosis using deep learning from clinical MRI. PLoS One 16:e0255939 10.1371/journal.pone.0255939 PMC8409666 34469432

[CR49] Ganjgahi H, Häring DA, Aarden P, Graham G, Sun Y, Gardiner S, Su W, Berge C, Bischof A, Fisher E et al (2025) AI-driven reclassification of multiple sclerosis progression. Nat Med 31:3414–3424 10.1038/s41591-025-03901-6 PMC12532606 40835969

[CR51] Graus F, Titulaer MJ, Balu R, Benseler S, Bien CG, Cellucci T, Cortese I, Dale RC, Gelfand JM, Geschwind M et al (2016) A clinical approach to diagnosis of autoimmune encephalitis. Lancet Neurol 15:391–404 10.1016/S1474-4422(15)00401-9 PMC5066574 26906964

[CR52] Greselin M, Lu PJ, Mroczek M, Cerdá-Fuertes N, Demirtzoglou A, Papadopoulou A, Kuhle J, Leppert D, Arnould S, Aoun M et al (2025) AI-assisted identification of disability patterns within identical EDSS grades. Mult Scler 31:677–688 10.1177/13524585251327300 PMC12092942 40248861

[CR53] Gu S, Bao T, Wang T, Yuan Q, Yu W, Lin J, Zhu H, Cui S, Sun Y, Jia X et al (2025) Multimodal AI diagnostic system for neuromyelitis optica based on ultrawide-field fundus photography. Front Med 12:1555380 10.3389/fmed.2025.1555380 PMC12092233 40400634

[CR54] Guo J, Dong R, Zhang R, Yang F, Wang Y, Miao W (2025) Interpretable machine learning model for predicting the prognosis of antibody positive autoimmune encephalitis patients. J Affect Disord 369:352–363 10.1016/j.jad.2024.10.010 39374738

[CR55] Guo J, Zou Y (2026) Machine learning and multi-omics integration identifies immunological predictors and mechanistic insights in autoimmune encephalitis. Inflamm Res 75:18 10.1007/s00011-025-02180-8 41533076

[CR57] Hagiwara A, Otsuka Y, Andica C, Kato S, Yokoyama K, Hori M, Fujita S, Kamagata K, Hattori N, Aoki S (2021) Differentiation between multiple sclerosis and neuromyelitis optica spectrum disorders by multiparametric quantitative MRI using convolutional neural network. J Clin Neurosci 87:55–58 10.1016/j.jocn.2021.02.018 33863534

[CR58] Havaei M, Guizard N, Chapados N, Bengio Y (2016) HeMIS: hetero-modal image segmentation. Med Image Comput Comput-Assist Interven—MICCAI 2016, LNCS 9901:469–477

[CR59] He T, Mao Y, Zhang Z, Zhuo Z, Duan Y, Wu L, Li Y, Zhang N, Han X, Zhu Y et al (2022) A multicenter study of brain T2WI lesions radiomics machine learning models distinguishing multiple sclerosis and neuromyelitis optica spectrum disorder. Chin J Radiol 56:1332–1338

[CR60] Hecker M, Frahm N, Zettl UK (2024) Update and application of a deep learning model for the prediction of interactions between drugs used by patients with multiple sclerosis. Pharmaceutics 16:3 10.3390/pharmaceutics16010003 PMC10819178 38276481

[CR61] Heming M, Börsch AL, Wiendl H, Meyer zu Hörste G (2022) High-dimensional investigation of the cerebrospinal fluid to explore and monitor CNS immune responses. Genome Med 14:94 10.1186/s13073-022-01097-9 PMC9385102 35978442

[CR62] Hernán MA, Robins JM (2016) Using big data to emulate a target trial when a randomized trial is not available. Am J Epidemiol 183:758–764 10.1093/aje/kwv254 PMC4832051 26994063

[CR63] Hindawi S, Szubstarski B, Boernert E, Tackenberg B, Wuerfel J (2025) Federated learning for lesion segmentation in multiple sclerosis: a real-world multi-center feasibility study. Front Neurol 16:1620469 10.3389/fneur.2025.1620469 PMC12457141 41001196

[CR64] Hochreiter S, Schmidhuber J (1997) Long short-term memory. Neural Comput 9:1735–1780 10.1162/neco.1997.9.8.1735 9377276

[CR65] Hossain MZ, Daskalaki E, Brüstle A, Desborough J, Lueck CJ, Suominen H (2022) The role of machine learning in developing non-magnetic resonance imaging based biomarkers for multiple sclerosis: a systematic review. BMC Med Inf Decis Mak 22:242 10.1186/s12911-022-01985-5 PMC9476596 36109726

[CR66] Huang C, Chen W, Liu B, Yu R, Chen X, Tang F, Liu J, Lu W (2022) Transformer-based deep-learning algorithm for discriminating demyelinating diseases of the central nervous system with neuroimaging. Front Immunol 13:897959 10.3389/fimmu.2022.897959 PMC9238435 35774780

[CR67] Huang J, Khademi M, Fugger L, Lindhe Ö, Novakova L, Axelsson M, Malmeström C, Constantinescu C, Lycke J, Piehl F et al (2020) Inflammation-related plasma and CSF biomarkers for multiple sclerosis. Proc Natl Acad Sci USA 117:12952–12960 10.1073/pnas.1912839117 PMC7293699 32457139

[CR70] Isensee F, Jaeger PF, Kohl SAA, Petersen J, Maier-Hein KH (2021) nnU-Net: a self-configuring method for deep learning-based biomedical image segmentation. Nat Methods 18:203–211 10.1038/s41592-020-01008-z 33288961

[CR71] Kelly E, Varosanec M, Kosa P, Prchkovska V, Moreno-Dominguez D, Bielekova B (2022) Machine learning-optimized Combinatorial MRI scale (COMRISv2) correlates highly with cognitive and physical disability scales in multiple sclerosis patients. Front Radiol 2:1026442 10.3389/fradi.2022.1026442 PMC10365117 37492667

[CR72] Kim C, Gadgil SU, DeGrave AJ, Omiye JA, Cai ZR, Daneshjou R, Lee SI (2024) Transparent medical image AI via an image–text foundation model grounded in medical literature. Nat Med 30:1154–1165 10.1038/s41591-024-02887-x 38627560

[CR73] Kim H, Lee Y, Kim YH, Lim YM, Lee JS, Woo J, Jang SK, Oh YJ, Kim HW, Lee EJ et al (2020) Deep learning-based method to differentiate neuromyelitis optica spectrum disorder from multiple sclerosis. Front Neurol 11:599042 10.3389/fneur.2020.599042 PMC7734316 33329357

[CR74] Klistorner S, Eghtedari M, Graham SL, Klistorner A (2022) Analysis of multifocal visual evoked potentials using artificial intelligence algorithms. Transl Vis Sci Technol 11:10 10.1167/tvst.11.1.10 PMC8762715 35006263

[CR75] Kodosaki E, Watkins WJ, Loveless S, Kreft KL, Richards A, Anderson V, Hurler L, Robertson NP, Zelek WM, Tallantyre EC (2024) Combination protein biomarkers predict multiple sclerosis diagnosis and outcomes. J Neuroinflammation 21:52 10.1186/s12974-024-03036-4 PMC10874571 38368354

[CR76] LeCun Y, Bengio Y, Hinton G (2015) Deep learning. Nature 521:436–444 10.1038/nature14539 26017442

[CR77] Li X, Kong X, Li A, Gong X, Guo K, Liu X, Liu Y, Zhang T, Deng X, Li J et al (2026) Machine learning model predicts prognosis in SARS-CoV-2-infected autoimmune encephalitis patients. Mult Scler Relat Disord 109:107028 10.1016/j.msard.2026.107028 41762950

[CR79] Liu X, Cruz Rivera S, Moher D, Calvert MJ, Denniston AK, SPIRIT-AI and CONSORT-AI Working Group (2020) Reporting guidelines for clinical trial reports for interventions involving artificial intelligence: the CONSORT-AI extension. Lancet Digit Health 2:e537–e548 10.1016/S2589-7500(20)30218-1 PMC8183333 33328048

[CR80] Lodé B, Hussein BR, Meurée C, Walsh R, Gaubert M, Lassalle N, Courbon G, Martin A, Le Bars J, Durand-Dubief F et al (2025) Evaluation of a deep learning segmentation tool to help detect spinal cord lesions from combined T2 and STIR acquisitions in people with multiple sclerosis. Eur Radiol 35:5954–5964 10.1007/s00330-025-11541-0 PMC12417267 40185925

[CR81] Lundberg SM, Lee SI (2017) A unified approach to interpreting model predictions. Adv Neural Inf Process Syst 30:4765–4774

[CR82] Luo X, Li H, Xia W, Quan C, ZhangBao J, Tan H, Wang N, Bao Y, Geng D, Li Y et al (2024) Joint radiomics and spatial distribution model for MRI-based discrimination of multiple sclerosis, neuromyelitis optica spectrum disorder, and myelin-oligodendrocyte-glycoprotein-IgG-associated disorder. Eur Radiol 34:4364–4375 10.1007/s00330-023-10529-y 38127076

[CR83] Manning AR, Letchuman V, Martin ML, Gombos E, Robert-Fitzgerald T, Cao Q, Raza P, O’Donnell CM, Renner B, Daboul L et al (2025) Multicenter automated central vein sign detection performs as well as manual assessment for the diagnosis of multiple sclerosis. AJNR Am J Neuroradiol 46:620–626 10.3174/ajnr.A8510 PMC11979820 39332906

[CR84] McMahan B, Moore E, Ramage D, Hampson S, Agüera y Arcas B (2017) Communication-efficient learning of deep networks from decentralized data. Proc Mach Learn Res 54:1273–1282

[CR85] Meng H, Tang Y, Qi Y, Zhou Q, He L, Chen S (2025) The potential of DeepSeek for AI-aided diagnosis of antibody-positive autoimmune encephalitis: a single-center, retrospective, observational study. Front Artif Intell 8:1638904 10.3389/frai.2025.1638904 PMC12536004 41122105

[CR86] Mizoguchi T, Hara M, Hirose S, Nakajima H (2022) Novel qEEG biomarker to distinguish anti-NMDAR encephalitis from other types of autoimmune encephalitis. Front Immunol 13:845272 10.3389/fimmu.2022.845272 PMC8885512 35242143

[CR87] Mongan J, Moy L, Kahn CE Jr (2020) Checklist for artificial intelligence in medical imaging (CLAIM): a guide for authors and reviewers. Radiol Artif Intell 2:e200029 10.1148/ryai.2020200029 PMC8017414 33937821

[CR88] Montalban X, Lebrun-Frenay C, Oh J, Arrambide G, Moccia M, Amato MP, Amezcua L, Banwell B, Bar-Or A, Barkhof F et al (2025) Diagnosis of multiple sclerosis: 2024 revisions of the McDonald criteria. Lancet Neurol 24:850–865 10.1016/S1474-4422(25)00270-4 40975101

[CR89] Moseley CE, Sagan S, Boquett JA, Spencer CM, Hollenbach JA, Sobel RA, Rothbard JB, Steinman L, Sabatino JJ Jr, Zamvil SS (2026) Sjögren syndrome candidate autoantigen AQP5 triggers AQP4 CNS autoimmunity through self-antigen mimicry. Neurol Neuroimmunol Neuroinflamm 13:e200517 10.1212/NXI.0000000000200517 PMC12643524 41270250

[CR91] Nabizadeh F, Ramezannezhad E, Kargar A, Sharafi AM, Ghaderi A (2023) Diagnostic performance of artificial intelligence in multiple sclerosis: a systematic review and meta-analysis. Neurol Sci 44:499–517 10.1007/s10072-022-06460-7 36303065

[CR93] National Medical Products Administration (2021) Principles for the classification defining of artificial intelligence-based medical software products. NMPA Announcement No. 47 of 2021.

[CR95] Nelson RE, Butler J, LaFleur J, Knippenberg K, Kamauu AWC, DuVall SL (2016) Determining multiple sclerosis phenotype from electronic medical records. J Manag Care Spec Pharm 22:1377–1382 10.18553/jmcp.2016.22.12.1377 PMC10398245 27882837

[CR96] Norgeot B, Quer G, Beaulieu-Jones BK, Torkamani A, Dias R, Gianfrancesco M, Arnaout R, Kohane IS, Saria S, Topol E et al (2020) Minimum information about clinical artificial intelligence modeling: the MI-CLAIM checklist. Nat Med 26:1320–1324 10.1038/s41591-020-1041-y PMC7538196 32908275

[CR97] Pakeerathan T, Davis J, Henderson AD, Sotirchos ES, Said Y, Havla J, Ringelstein M, Aktas O, Weise M, Gernert JA et al (2026) OCT-based differentiation of first acute optic neuritis: an international study of 111 patients with NMOSD and MOGAD. Neurol Neuroimmunol Neuroinflamm 13:e200531 10.1212/NXI.0000000000200531 PMC12794538 41499724

[CR98] Pati S, Baid U, Edwards B, Sheller M, Wang SH, Reina GA, Foley P, Gruzdev A, Karkada D, Davatzikos C et al (2022) Federated learning enables big data for rare cancer boundary detection. Nat Commun 13:7346 10.1038/s41467-022-33407-5 PMC9722782 36470898

[CR101] Petrosian D, Giedraitienė N, Taluntienė V, Apynytė D, Bikelis H, Makarevičius G, Jokubaitis M, Vaišvilas M (2025) Differential diagnosis of infectious versus autoimmune encephalitis using artificial intelligence-based modeling. J Clin Med 14:8222 10.3390/jcm14228222 PMC12653740 41303254

[CR102] Pirmani A, De Brouwer E, Arany Á, Oldenhof M, Passemiers A, Faes A, Kalincik T, Ozakbas S, Gouider R, Willekens B et al (2025) Personalized federated learning for predicting disability progression in multiple sclerosis using real-world routine clinical data. NPJ Digit Med 8:478 10.1038/s41746-025-01788-8 PMC12289948 40707601

[CR103] Rechtman A, Friedman-Korn T, Zamir S, Zveik O, Levin N, Vaknin-Dembinsky A (2025) A predictive model for triple negative optic neuritis patients: identifying the subset of patients with favorable outcomes. Mult Scler Relat Disord 103:106717 10.1016/j.msard.2025.106717 40934846

[CR104] Rieke N, Hancox J, Li W, Milletarì F, Roth HR, Albarqouni S, Bakas S, Galtier MN, Landman BA, Maier-Hein K et al (2020) The future of digital health with federated learning. NPJ Digit Med 3:119 10.1038/s41746-020-00323-1 PMC7490367 33015372

[CR107] Ronneberger O, Fischer P, Brox T (2015) U-Net: convolutional networks for biomedical image segmentation. In: Navab N, Hornegger J, Wells WM, Frangi AF (eds) Medical image computing and computer-assisted intervention—MICCAI 2015. Springer, Cham, pp 234–241

[CR108] Scarselli F, Gori M, Tsoi AC, Hagenbuchner M, Monfardini G (2009) The graph neural network model. IEEE Trans Neural Netw 20:61–80 10.1109/TNN.2008.2005605 19068426

[CR109] Schäfer R, Nicke T, Höfener H, Lange A, Merhof D, Feuerhake F, Schulz V, Lotz J, Kiessling F (2024) Overcoming data scarcity in biomedical imaging with a foundational multi-task model. Nat Comput Sci 4:495–509 10.1038/s43588-024-00662-z PMC11288886 39030386

[CR110] Seccia R, Romano S, Salvetti M, Crisanti A, Palagi L, Grassi F (2021) Machine learning use for prognostic purposes in multiple sclerosis. Life 11:122 10.3390/life11020122 PMC7914671 33562572

[CR111] Selvaraju RR, Cogswell M, Das A, Vedantam R, Parikh D, Batra D (2020) Grad-CAM: visual explanations from deep networks via gradient-based localization. Int J Comput Vis 128:336–359

[CR112] Shariff Y, Khalid M, Muhammad T, Waafira A (2025) Machine learning and neuromyelitis optica: pioneering precision in disease diagnosis. Ann Med Surg (Lond) 87:6882–6883 10.1097/MS9.0000000000003796 PMC12577950 41181458

[CR113] Shen S, Wei R, Gao Y, Yang X, Zhang G, Yan B, Xiao Z, Li J (2024) Cortical atrophy in early-stage patients with anti-NMDA receptor encephalitis: a machine-learning MRI study with various feature extraction. Cereb Cortex 34:bhad499 10.1093/cercor/bhad499 38185983

[CR114] Sounderajah V, Guni A, Liu X, Collins GS, Karthikesalingam A, Markar SR, Golub RM, Denniston AK, Shetty S, Moher D et al (2025) The STARD-AI reporting guideline for diagnostic accuracy studies using artificial intelligence. Nat Med 31:3283–3289 10.1038/s41591-025-03953-8 40954311

[CR115] Stake J, Spiekers C, Akkurt BH, Heindel W, Brix T, Mannil M, Musigmann M (2024) Prediction of seropositivity in suspected autoimmune encephalitis by use of radiomics: a radiological proof-of-concept study. Diagnostics 14:1070 10.3390/diagnostics14111070 PMC11171889 38893597

[CR116] Storelli L, Azzimonti M, Gueye M, Vizzino C, Preziosa P, Tedeschi G, De Stefano N, Pantano P, Filippi M, Rocca MA (2022) A deep learning approach to predicting disease progression in multiple sclerosis using magnetic resonance imaging. Invest Radiol 57:423–432 10.1097/RLI.0000000000000854 35093968

[CR117] Sun Y, Sun R, Lv J, Kong Q, Dai C, Wang B, Han X, Chen M, Liu R, Jiang Y et al (2025a) Diagnosis and subtyping of autoimmune encephalitis using an attention-based multi-instance learning model: a Multi-center 18F-FDG PET Study. CNS Neurosci Ther 31:e70513 10.1111/cns.70513 PMC12320127 40755278

[CR118] Sun Y, Wang L, Li G, Lin W, Wang L (2025b) A foundation model for enhancing magnetic resonance images and downstream segmentation, registration and diagnostic tasks. Nat Biomed Eng 9:521–538 10.1038/s41551-024-01283-7 PMC12360180 39638876

[CR119] Takai Y, Hametner S, Riedl C, Misu T, Takahashi T, Suzuki H, Chihara N, Watanabe M, Miyahara H, Yoshida M et al (2026) Characteristic patterns of complement deposition in NMOSD, MOGAD, and MS. Acta Neuropathol 151:13 10.1007/s00401-026-02985-9 PMC12886220 41661329

[CR120] Thakur SP, Schindler MK, Bilello M, Bakas S (2022) Clinically deployed computational assessment of multiple sclerosis lesions. Front Med 9:797586 10.3389/fmed.2022.797586 PMC8968446 35372431

[CR121] Varley JA, Champsas D, Prossor T, Pontillo G, Abdel-Mannan O, Khaleeli Z, Petzold A, Toosy AT, Trip SA, Wilson H et al (2024) Validation of the 2023 international diagnostic criteria for MOGAD in a selected cohort of adults and children. Neurology 103:e209321 10.1212/WNL.0000000000209321 PMC11244737 38870448

[CR122] Vasey B, Nagendran M, Campbell B, Clifton DA, Collins GS, Denaxas S, Denniston AK, Faes L, Geerts B, Ibrahim M et al (2022) Reporting guideline for the early-stage clinical evaluation of decision support systems driven by artificial intelligence: DECIDE-AI. Nat Med 28:924–933 10.1038/s41591-022-01772-9 35585198

[CR123] Vaswani A, Shazeer N, Parmar N, Uszkoreit J, Jones L, Gomez AN, Kaiser Ł, Polosukhin I (2017) Attention is all you need. Adv Neural Inf Process Syst 30:5998–6008

[CR124] Vázquez-Marrufo M, Sarrias-Arrabal E, García-Torres M, Martín-Clemente R, Izquierdo G (2023) A systematic review of the application of machine-learning algorithms in multiple sclerosis. Neurologia 38:577–590 10.1016/j.nrleng.2020.10.013 35843587

[CR125] Villar-Delfino PH, Pereira Christo P, Volpe CMO (2025) Drug repurposing and artificial intelligence in multiple sclerosis: emerging strategies for precision therapy. Sclerosis 3:28

[CR126] Wager S, Athey S (2018) Estimation and inference of heterogeneous treatment effects using random forests. J Am Stat Assoc 113:1228–1242

[CR128] Wang L, Du L, Li Q, Li F, Wang B, Zhao Y, Meng Q, Li W, Pan J, Xia J et al (2022) Deep learning-based relapse prediction of neuromyelitis optica spectrum disorder with anti-aquaporin-4 antibody. Front Neurol 13:947974 10.3389/fneur.2022.947974 PMC9389264 35989911

[CR133] Wiltgen T, McGinnis J, Schlaeger S, Kofler F, Voon CC, Berthele A, Bischl D, Grundl L, Will N, Metz M et al (2024) LST-AI: a deep learning ensemble for accurate MS lesion segmentation. Neuroimage Clin 42:103611 10.1016/j.nicl.2024.103611 PMC11088188 38703470

[CR134] Wu C, Fang Y, Zhou Y, Wu H, Huang S, Zhu S (2022) Risk prediction models for early ICU admission in patients with autoimmune encephalitis: integrating scale-based assessments of disease severity. Front Immunol 13:916111 10.3389/fimmu.2022.916111 PMC9226454 35757708

[CR135] Xia Z, Chikersal P, Venkatesh S, Walker E, Dey AK, Goel M (2025) Longitudinal digital phenotyping of multiple sclerosis severity using passively sensed behaviors and ecological momentary assessments: real-world evaluation. J Med Internet Res 27:e70871 10.2196/70871 PMC12174892 40459924

[CR136] Xiang Y, Dong X, Zeng C, Liu J, Liu H, Hu X, Feng J, Du S, Wang J, Han Y et al (2022a) Clinical variables, deep learning and radiomics features help predict the prognosis of adult anti-N-methyl-D-aspartate receptor encephalitis early: a Two-center Study in Southwest China. Front Immunol 13:913703 10.3389/fimmu.2022.913703 PMC9199424 35720336

[CR137] Xiang Y, Zeng C, Liu B, Tan W, Wu J, Hu X, Han Y, Luo Q, Gong J, Liu J et al (2022b) Deep learning-enabled identification of autoimmune encephalitis on 3D multi-sequence MRI. J Magn Reson Imaging 55:1082–1092 10.1002/jmri.27909 34478565

[CR138] Xie Z, Zhang J, Liu L, Hu E, Wang J (2025) Prediction model for severe autoimmune encephalitis: a tool for risk assessment and individualized treatment guidance. Front Neurol 16:1575835 10.3389/fneur.2025.1575835 PMC11958171 40170898

[CR139] Xu Y, Meng H, Fan M, Yin L, Sun J, Yao Y, Wei Y, Cong H, Wang H, Song T et al (2024) A simple score (MOG-AR) to identify individuals at high risk of relapse after MOGAD attack. Neurol Neuroimmunol Neuroinflamm 11:e200309 10.1212/NXI.0000000000200309 PMC11385954 39250723

[CR140] Yang W, Liu C, Li Z, Cui M (2025) Multi-omic biomarkers associated with multiple sclerosis: from Mendelian randomization to drug prediction. Sci Rep 15:9421 10.1038/s41598-025-94303-8 PMC11923301 40108295

[CR141] Yang X, Li F, Zhe J, Zhang Z, Hu Z, Zhang C, Wan F, Xie Q, Li X (2025) Single-cell RNA sequencing of cerebrospinal fluid immune cells in relapsing-remitting multiple sclerosis: insights into cellular composition and immune dynamics. J Neurol 272:778 10.1007/s00415-025-13505-2 41261231

[CR142] Yeh WZ, Francis A, Butzkueven H, Geraldes R, Leite MI, Palace J (2026) Prediction of relapse in myelin oligodendrocyte glycoprotein antibody-associated disease: external validation of the MOG-AR score. J Neurol 273:224 10.1007/s00415-026-13754-9 PMC13009136 41870604

[CR143] Yu K, Jiang R, Li Z, Ren X, Jiang H, Zhao Z (2024) Integrated analyses of single-cell transcriptome and Mendelian randomization reveal the protective role of FCRL3 in multiple sclerosis. Front Immunol 15:1428962 10.3389/fimmu.2024.1428962 PMC11284051 39076991

[CR144] Zhang K, Zhou R, Adhikarla E, Yan Z, Liu Y, Yu J, Liu Z, Chen X, Davison BD, Ren H et al (2024) A generalist vision–language foundation model for diverse biomedical tasks. Nat Med 30:3129–3141 10.1038/s41591-024-03185-2 PMC12581140 39112796

[CR146] Zhang Y, Shi X, Fan Z, Tu E, Wu D, Leng X, Wan T, Wang X, Wang X, Lu W et al (2025) Machine learning for the early prediction of long-term cognitive outcome in autoimmune encephalitis. J Psychosom Res 190:112051 10.1016/j.jpsychores.2025.112051 39978283

[CR147] Zhao W, Wu C, Fan Y, Qiu P, Zhang X, Sun Y, Zhou X, Zhang S, Peng Y, Wang Y et al (2026) An agentic system for rare disease diagnosis with traceable reasoning. Nature 651:775–784 10.1038/s41586-025-10097-9 PMC12999473 41708847

[CR148] Zhou Q, Xie Z, He L, Sun G, Meng H, Luo Z, Feng Y, Chu X, Li L, Zhang J et al (2024) Multi-omics profiling reveals peripheral blood biomarkers of multiple sclerosis: implications for diagnosis and stratification. Front Pharm 15:1458046 10.3389/fphar.2024.1458046 PMC11384994 39257402

