# Supplementary material for: Artificial intelligence in central nervous system autoimmune diseases: from clinical decision support to mechanistic discovery and translation
Source: EMBO Mol Med. 2026 Oct 13;18(11):4179–99. doi: 10.1038/s44321-026-00527-0 (PMC13748597; doi:10.1038/s44321-026-00527-0)
Supplement: Supplementary file 1 [file 44321_2026_527_MOESM6_ESM.pdf]

EMBO Molecular Medicine

Artificial Intelligence in Central Nervous System Autoimmune Diseases

Zijian Cao, Pei Zheng, Zhihui Qi, Yang Yao, Donglin Xie, Ya Zi HE, Shenghua Zong, Zongjiu Zhang, and Fu-Dong Shi *Corresponding author(s): Fu-Dong Shi (fshi@tmu.edu.cn) , Zongjiu Zhang (zhangzongjiu@tsinghua.edu.cn)*

Review Timeline:

Submission Date:	9th Jul 26
Editorial Decision:	4th Aug 26
Revision Received:	24th Aug 26
Accepted:	9th Sep 26

Editor: Zeljko Durdevic

Transaction Report:

(Note: With the exception of the correction of typographical or spelling errors that could be a source of ambiguity, letters and reports are not edited. Depending on transfer agreements, referee reports obtained elsewhere may or may not be included in this compilation. Referee reports are anonymous unless the Referee chooses to sign their reports.)

4th Aug 2026

Dear Prof. Shi,

Thank you for the submission of your manuscript to EMBO Molecular Medicine. We have now received feedback from the three reviewers who agreed to evaluate your manuscript. As you will see from the reports below, the referees are positive about its interest and timeliness, however, they also raise serious criticisms that should be fully addressed in a major revision of the current manuscript.

I would also like to ask you to amend the following:
1) Upon editorial assessment we may send the article to our graphic artist who will re-draw the figures for style and clarity. Therefore, please submit your revised figures as individual high-resolution files in an editable format (e.g. PDF with the option 'editable labels' checked).
2) Move Tables after figure legends.
3) Please add "Glossary". The glossary is meant to explain some of the terms used for laymen. Could you please identify terms that may need an "explanation"?
4) Pending issues: At the end of each article is a box highlighting issues that still need further studies and where research efforts should converge. Could you identify some pending issues?
5) Merge funding with "Acknowledgments" section that should include information of all sources of funding.
6) Please remove Data Availability section and Ethics Approval.
7) In the reference list please remove DOIs for published articles. Please check "Author Guidelines" for more information. https://www.embopress.org/page/journal/17574684/authorguide#referencesformat
8) As part of the EMBO Publications transparent editorial process initiative EMBO Molecular Medicine will publish online a Review Process File (RPF) to accompany accepted manuscripts. This file will be published in conjunction with your paper and will include the anonymous referee reports, your point-by-point response and all pertinent correspondence relating to the manuscript. Let us know whether you agree with the publication of the RPF.
9) Please provide a point-by-point letter INCLUDING my comments as well as the reviewer's reports and your detailed responses (as Word file).

You can submit your revised files by logging onto our online manuscript tracking system or simply follow this link:

I hope that the referees' comments do not prove too problematic to address and I look forward to reading your next version.

Yours sincerely,

Zeljko Durdevic

Zeljko Durdevic
Senior Editor
EMBO Molecular Medicine

*** IMPORTANT INFORMATION ***

When submitting your revised manuscript, please include:

1) a .doc formatted version of the manuscript text (including Figure legends and tables)
2) Separate figure files
3) a letter INCLUDING the reviewer's reports and your detailed responses to their comments.

Also, and to save some time should your paper be accepted, please read below for additional information regarding some

features of our research articles:

1) Glossary: EMBO Molecular Medicine articles will be accompanied by a glossary explaining some of the terms used for laymen. I identified the following:

____, ____, ____

Could you please help us in identifying terms that may need an "explanation" other terms that we can add to the glossary.

2) For more information: This is a short list of related web links for further consultation by the readers. Could you identify some relevant ones? Examples are patient associations, OMIM related links, databases, authors websites, etc.

3) Pending issues: At the end of each article we will have a box highlighting issues that still need further studies and where research efforts should converge (we call this the Pending issues box). From my reading I would say:

----

but I can see there may be many more. Could you work on this as well?

4) Disclosure and competing interest statement: Please include a statement declaring any competing commercial interests in relation to your submitted work.

5) Please note that we now mandate that all corresponding authors list an ORCID digital identifier. This takes <90 seconds to complete. We encourage all authors to supply an ORCID identifier, which will be linked to their name for unambiguous name identification.

Currently, our records indicate that the ORCID for your account is 0000-0002-9675-4637.

Please click the link below to modify this ORCID:
Link Not Available
-

Thank you,

Zeljko Durdevic

Zeljko Durdevic
Editor
EMBO Molecular Medicine

***** Reviewer's comments *****

Referee #1 (Remarks for Author):

The manuscript by Cao et al., reviews using artificial intelligence (AI) in central nervous system (CNS) autoimmune disorders such as MS, NMO, MOGAD, and autoimmune enecephalitis. In particular, the authors note the potential uses of AI ranging from differential diagnoses of these different conditions to predicting therapy response. The manuscript is well done by experts in the field and should be of interest to a broad audience. Expanding on some of the background of these distinct diseases and some additional references should improve some of the teaching points in the manuscript.

Major Concerns
1. None

Minor Concerns
1. I recommend mentioning an additional NMO feature that could improve AI informed differential diagnosis or therapy algorithms, which is the very high rates of Sjögren's Disease and Lupus with NMO, but not MOGAD or MS. This is an underexplored area of neuroimmunology that AI could assist in untangling. Some recent citations about potential reasons for the overlap of these conditions that would be good to include are Moseley et al, Neurology: Neuroimmunology & Neuroinflammation 2026 (PMID 41270250) and Attfiled et al, Lancet Neurology 2026 (PMID 42009010).

2. The authors nicely mention how AI might predict treatment resistance and worsened outcomes. I suggest the authors note that MOGAD is a prime example of this, as a large fraction have dramatic recovery, but a subset have extremely poor recovery. Some early studies are beginning to point to why this may be, such as complement deposition in the severe cases (Takai et al, Acta Neuropathologica 2026, PMID 41661329). AI tools may help us to predict which of these patients will have worse outcomes at onset and guide therapy.

3. In section 6.1.2 on MS lesion analyses, the authors may wish to cite the 2024 MS Diagnostic Criteria to highlight that MRI findings that were recently only used in research (PRLs and CVS) are now part of formal diagnostic criteria.

4. In section 6.1.4, the citation for the 2023 MOGAD Diagnostic Criteria should be Banwell et al, Lancet Neurology 2023.

Referee #2 (Remarks for Author):

This is a comprehensive and timely review covering the rapidly evolving role of artificial intelligence in CNS autoimmune diseases. The manuscript is well structured, generally well written, and spans the field from clinical decision support to mechanistic discovery and translational applications. The integration of imaging AI, multi-omics, and emerging large language models provides a broad perspective that will be valuable to readers from both neuroimmunology and computational medicine.

I nevertheless have several suggestions that would strengthen the manuscript.

Strengthen the critical appraisal of the evidence.
The review appropriately highlights methodological limitations, but many cited AI studies are presented primarily through performance metrics (e.g., AUC, Dice). A more balanced discussion of common biases-including spectrum bias, data leakage, overfitting, annotation variability, and limited external validation-would help readers better judge the maturity of the field.
Differentiate established clinical applications from future perspectives.
The manuscript occasionally presents mature applications (e.g., MS lesion segmentation) alongside speculative developments (e.g., agentic LLMs or AI-guided therapy selection) without sufficiently distinguishing current clinical reality from future possibilities. A table categorizing applications according to technology readiness or evidence level would be valuable.
Expand discussion of foundation models.
The section on large language models is interesting but relatively brief. Given the rapid emergence of multimodal foundation models capable of jointly processing imaging and text, the authors could discuss how these models differ from conventional supervised machine learning, their potential advantages, and their current limitations in neuroimmunology.
Address regulatory and implementation challenges in greater depth.
Clinical deployment depends not only on model performance but also on regulatory approval, workflow integration, reimbursement, medico-legal responsibility, cybersecurity, and continuous post-deployment monitoring. These practical barriers deserve additional discussion.
Improve discussion of multimodal data fusion.
Multimodal integration is a recurring theme, but the review could better explain practical challenges such as missing modalities, temporal alignment, heterogeneous data quality, harmonization across institutions, and uncertainty estimation.
Include a stronger discussion of explainability and trustworthiness.
While SHAP and Grad-CAM are introduced, it would be useful to emphasize that explainability methods alone do not establish model reliability. Calibration, uncertainty estimation, robustness testing, fairness analyses, and prospective validation should receive similar emphasis.
Provide a clearer roadmap for future research.
The review summarizes many developments but would benefit from a concluding figure outlining key priorities over the next 5-10 years, including multicenter prospective validation, federated learning, standardized datasets, multimodal foundation models, and clinical implementation studies.
Minor editorial comments.
Ensure consistent terminology throughout the manuscript when referring to AI, machine learning, and deep learning.
Some tables are very dense and could be simplified to improve readability.
Consider reducing repetition regarding external validation and reporting guidelines across multiple sections.

Overall, this is a well-written and comprehensive review on an important topic. The suggested revisions are intended to further improve clarity, critical balance, and clinical relevance before publication.

Referee #3 (Remarks for Author):

This review summarizes current and emerging applications of AI in CNS autoimmune diseases, highlighting recent clinical advances and the challenges to implementing precision neuroimmunology.
Below my comments:
- The Introduction would benefit from a concise disease-specific framework linking biological heterogeneity, available biomarkers, and unresolved clinical decision points to the AI applications discussed in the subsequent sections.
- The manuscript combines features of a narrative and scoping review. The authors should clarify its intended methodology and, if applicable, briefly report the search strategy, eligibility criteria, study selection, and data-extraction process. Otherwise, claims implying comprehensive evidence coverage should be moderated.
- Given the limited evidence base in NMOSD, MOGAD, and AE, the manuscript should be framed primarily as a mapping of emerging applications and unmet methodological needs rather than as a definitive assessment of translational maturity.
- The definition of AI is overly broad and includes conventional statistical methods, causal-inference approaches, and standard bioinformatic pipelines. A clearer taxonomy is needed to distinguish these methods and define which components are genuinely AI-based.
- In Table 1, "multimodal fusion" is too broadly defined and provides limited methodological or clinical information. The authors should specify the main fusion strategies, the types and timing of data being integrated, and representative disease-specific applications.
- The discussion of multimodal and longitudinal modelling should address practical challenges, including missing modalities, asynchronous measurements, temporal leakage, treatment exposure, model drift, and cross-center transportability.
- The limitations of SHAP and Grad-CAM are appropriately acknowledged in Section 3.3; however, this caution should be applied consistently throughout the disease-specific sections and tables, where explainability methods are occasionally used as indicators of methodological strength or individualized clinical guidance.
- Although Section 3.4 appropriately outlines general methodological limitations and reporting standards, these criteria are not consistently applied to the individual studies summarized in the disease-specific sections and tables. The review would therefore benefit from a more critical appraisal of geographical, demographic, age-related, and healthcare-system biases, as well as negative findings, failed validations, and publication bias, in order to avoid an overly optimistic representation of current AI performance, particularly in rare diseases and pediatric cohorts.
- The distinction between prognostic risk prediction and treatment-effect estimation is appropriately acknowledged in Section 4.4, but it should be applied consistently throughout the manuscript, particularly in Table 1 and in claims concerning "precision treatment recommendation" or treatment guidance.
- The sections on agentic systems and foundation models are appropriately forward-looking but should remain clearly separated from clinically validated applications. For federated learning, the authors should distinguish demonstrated feasibility in other medical domains from direct disease-specific evidence in CNS autoimmunity.
- The molecular-discovery sections and clinical decision-support address distinct questions and currently feel only loosely connected: their relationship should be clarified, or the two domains should be more clearly separated. Moreover, the molecular-discovery section should more clearly distinguish statistical association, causal prioritization, mechanistic hypothesis generation, experimental validation, druggability, and demonstrated therapeutic efficacy.
- Tables 3-6 should be standardized to report sample size, unit of analysis, study design, validation strategy, performance metrics with confidence intervals, comparators, and main methodological limitations.
- The translational-maturity criteria used in Figure 2 would benefit from further clarification. As each domain appears to be classified according to the highest stage reached by a cited study, the figure may not fully reflect the volume, consistency, or quality of the supporting evidence. The authors could therefore link each classification to specific studies and clearer criteria, or present the figure more explicitly as a qualitative overview.- Claims such as "first", "highest", "state-of-the-art", "near-perfect", and "clinical deployment" should be carefully verified and replaced with more cautious wording when direct evidence is lacking.
- The manuscript is often redundant, and several relatively simple concepts could be presented more concisely. Substantial repetition is particularly evident across Sections 2, 4, 6, and 7, which should be condensed and shortened to improve clarity, focus, and ease of consultation.

Response to Referee #1

General assessment

> *The manuscript by Cao et al., reviews using artificial intelligence (AI) in central nervous system (CNS) autoimmune disorders such as MS, NMO, MOGAD, and autoimmune encephalitis. In particular, the authors note the potential uses of AI ranging from differential diagnoses of these different conditions to predicting therapy response. The manuscript is well done by experts in the field and should be of interest to a broad audience. Expanding on some of the background of these distinct diseases and some additional references should improve some of the teaching points in the manuscript. Major Concerns: None.*

Response: We sincerely thank the Reviewer for the encouraging and generous assessment of our Review. We fully agree that additional disease-specific clinical context and updated references can strengthen the teaching value of the manuscript. We have therefore incorporated all four suggested disease-specific revisions and have also used these changes to sharpen the links between clinical heterogeneity and the AI applications discussed in the Review.

Referee #1 – Minor Concern 1

> *I recommend mentioning an additional NMO feature that could improve AI informed differential diagnosis or therapy algorithms, which is the very high rates of Sjögren's Disease and Lupus with NMO, but not MOGAD or MS. This is an underexplored area of neuroimmunology that AI could assist in untangling. Some recent citations about potential reasons for the overlap of these conditions that would be good to include are Moseley et al, Neurology: Neuroimmunology & Neuroinflammation 2026 (PMID 41270250) and Attfiled et al, Lancet Neurology 2026 (PMID 42009010).*

Response: In the revised manuscript, we have incorporated this issue into both the general clinical landscape and the NMOSD-specific discussion. We now note that existing NMOSD models are largely developed in established AQP4-IgG-positive cohorts using typical MS comparators, which limits their transportability to seronegative, overlap, and diagnostically uncertain populations. We further identify the integration of systemic-autoimmune history, rheumatological serology, imaging, and longitudinal electronic health record data as a potential multimodal approach for future investigation. The priority-gaps section and Table 7 now call for representative systemic-autoimmune-overlap cohorts and more complete reporting of coexisting autoimmunity and diagnostic uncertainty. We have also incorporated the suggested recent studies by Moseley et al. and Attfield et al. Because the clinical utility of such models has not yet been established, we present this as a research priority rather than as a validated diagnostic or treatment-selection capability.

Changes in the manuscript: Section 2 now identifies coexisting Sjögren disease and systemic lupus erythematosus as contributors to diagnostic-attribution uncertainty in AQP4-IgG-positive NMOSD and highlights the limited transportability of models developed predominantly in AQP4-IgG-positive cohorts with typical MS comparators. Section 6.2.1 now describes the integration of systemic-autoimmune history, rheumatological serology, imaging, and longitudinal electronic health record data as an underexplored multimodal AI application whose clinical utility remains unvalidated. Section 6.2.2 and Table 7 identify representative systemic-autoimmune-overlap cohorts, documentation of coexisting autoimmunity, and diagnostic uncertainty as priorities for future studies. The suggested references by Moseley et al. and Attfield et al. have also been added.

Location: Section 2; Sections 6.2.1 and 6.2.2; Table 7; References.

Referee #1 – Minor Concern 2

> *The authors nicely mention how AI might predict treatment resistance and worsened outcomes. I suggest the authors note that MOGAD is a prime example of this, as a large fraction have dramatic recovery, but a subset have extremely poor recovery. Some early studies are beginning to point to why this may be, such as complement deposition in the severe cases (Takai et al, Acta Neuropathologica 2026, PMID 41661329). AI tools may help us to predict which of these patients will have worse outcomes at onset and guide therapy.*

Response: We have revised the MOGAD section to make this clinical heterogeneity explicit and to distinguish recovery-oriented prediction from relapse-risk prediction. We also incorporated the findings of Takai et al., who described a destructive histopathological pattern in severe disease characterised by prominent C9neo deposition and oligodendrocyte loss. Because the association between this pathological pattern and clinical recovery has not been

validated, we present it as hypothesis-generating mechanistic evidence rather than as an established prognostic biomarker. The revised manuscript now identifies early, multimodal prediction of poor recovery as a potential future application of AI, using onset phenotype, MRI or OCT features, MOG-IgG status or titre, treatment exposure, and longitudinal outcomes. We further clarify that such models could support risk-informed follow-up and rehabilitation planning, but should not currently be interpreted as validated tools for individual treatment selection. The priority-gaps section has also been expanded to emphasise recovery magnitude, severe residual disability, visual and functional outcomes, and rehabilitation needs as targets for future multicentre studies.

Changes in the manuscript: Section 6.4.1 now explicitly describes the heterogeneity of recovery after MOGAD attacks, including the clinically important subgroup with incomplete or severe residual disability. It also incorporates the Takai et al. study describing prominent C9neo deposition and oligodendrocyte loss in severe disease and clarifies that this finding remains hypothesis-generating because its relationship to clinical recovery is unvalidated. The section now identifies early multimodal prediction of recovery trajectory as an unmet need and discusses the potential role of AI in risk-informed follow-up and rehabilitation planning. Section 6.4.2 has been revised to prioritise early prediction of recovery magnitude, severe residual disability, visual and functional outcomes, and rehabilitation needs, while clarifying that current relapse-risk models do not establish individual treatment benefit or validated treatment selection. The Takai et al. (2026) reference has been added and cited in the revised MOGAD discussion.

Location: Sections 6.4.1 and 6.4.2; References.

Referee #1 – Minor Concern 3

> *In section 6.1.2 on MS lesion analyses, the authors may wish to cite the 2024 MS Diagnostic Criteria to highlight that MRI findings that were recently only used in research (PRLs and CVS) are now part of formal diagnostic criteria.*

Response: We sincerely thank the Reviewer for this valuable suggestion. We agree that the 2024 revisions of the McDonald diagnostic criteria increase the clinical relevance of the central vein sign (CVS) and paramagnetic rim lesions (PRLs), which were previously used mainly as research-oriented MRI markers. We have revised the MS discussion to state that CVS and PRLs are recognised as supportive MRI biomarkers in defined diagnostic settings and therefore represent clinically relevant targets for automated imaging assessment. We also added and discussed representative multicentre studies evaluating automated PRL detection and CVS assessment. These studies illustrate the technical feasibility of AI-assisted assessment, while also highlighting the current limitations, including specialised susceptibility-sensitive MRI acquisition, motion-related processing failures, reader- or expert-label dependence, imperfect discrimination, and the lack of evidence that automated assessment improves clinical decisions or patient outcomes. We have therefore distinguished the increased diagnostic relevance of these MRI biomarkers from the separate question of routine AI-enabled implementation.

Changes in the manuscript: Section 2 now identifies automated CVS and PRL assessment as clinically relevant MRI-based applications in MS and notes that their clinical value still depends on external validation and evidence of impact on diagnostic or treatment decisions. Section 6.1.1 has been revised to state that the revised McDonald criteria recognise CVS and PRLs as supportive MRI biomarkers in defined diagnostic settings. The section also discusses the potential role of automated assessment and clarifies the limitations related to specialised MRI acquisition, motion, reader- or expert-label dependence, and limited evidence for routine implementation. Table 3 now includes representative multicentre studies of automated PRL detection and CVS assessment, together with their validation characteristics and methodological limitations. CVS and PRLs have also been defined in the Glossary, and the relevant references have been added.

Location: Section 2; Section 6.1.1; Table 2; Table 3; Table 7; Glossary; References.

Referee #1 – Minor Concern 4

> *In section 6.1.4, the citation for the 2023 MOGAD Diagnostic Criteria should be Banwell et al, Lancet Neurology 2023.*

Response: We verified that the relevant statement is located in the MOGAD section (Section 6.4.1) and replaced the citation to the later validation study with the original international diagnostic criteria proposed by Banwell et al.

in The Lancet Neurology in 2023. We also checked the manuscript for other references to the 2023 MOGAD diagnostic criteria and corrected them where necessary.

Changes in the manuscript: The diagnostic-criteria citation has been corrected to Banwell et al., 2023.

Location: Section 6.4.1.

Response to Referee #2

General assessment

> *This is a comprehensive and timely review covering the rapidly evolving role of artificial intelligence in CNS autoimmune diseases. The manuscript is well structured, generally well written, and spans the field from clinical decision support to mechanistic discovery and translational applications. The integration of imaging AI, multi-omics, and emerging large language models provides a broad perspective that will be valuable to readers from both neuroimmunology and computational medicine.*

Referee #2 – Comment 1: Strengthen the critical appraisal of the evidence

> *The review appropriately highlights methodological limitations, but many cited AI studies are presented primarily through performance metrics (e.g., AUC, Dice). A more balanced discussion of common biases-including spectrum bias, data leakage, overfitting, annotation variability, and limited external validation-would help readers better judge the maturity of the field.*

Response: We agree that AUC and Dice scores alone may give an overly optimistic impression of model performance. We have therefore expanded Section 3.4 to discuss spectrum bias, data leakage, overfitting, annotation variability, and limited external validation, and to explain why these factors affect the interpretation and transportability of reported results. We also revised Tables 3–6 and Supplementary Tables S2–S5 so that performance estimates are presented together with study design, validation strategy, reference standards, and study-specific limitations. The revised manuscript further notes the potential impact of publication bias and the underreporting of negative or failed external-validation results.

Changes in the manuscript: Section 3.4 now provides a structured discussion of spectrum bias, data leakage, overfitting, annotation variability, and insufficient external validation, and clarifies that AUC and Dice should be interpreted alongside calibration, confidence intervals, comparator performance, and clinically relevant error measures. Tables 3–6 and Supplementary Tables S2–S5 were revised to report study-specific design limitations and validation details, rather than performance metrics alone. Publication bias and the underreporting of negative or failed external validations are also noted.

Location: Section 3.4; Tables 3–6; Supplementary Tables S2–S5.

Referee #2 – Comment 2: Differentiate established clinical applications from future perspectives

> *The manuscript occasionally presents mature applications (e.g., MS lesion segmentation) alongside speculative developments (e.g., agentic LLMs or AI-guided therapy selection) without sufficiently distinguishing current clinical reality from future possibilities. A table categorizing applications according to technology readiness or evidence level would be valuable.*

Response: We agree that established imaging applications and forward-looking AI concepts should be distinguished more clearly. We therefore added a four-stage evidence framework in Table 2, ranging from conceptual evidence to workflow-evaluated or implemented applications, and aligned Figure 2 with the same definitions. The revised manuscript identifies selected MS imaging tasks as having stronger validation or workflow evidence, while agentic systems, foundation models, and individualised treatment-effect estimation remain conceptual, early empirical, or unmet applications. We also clarified throughout the text that this framework is a qualitative evidence map rather than a formal ranking of maturity or clinical readiness.

Changes in the manuscript: Table 2 now categorises major AI application domains using a four-stage evidence framework: conceptual, early empirical, validated research, and workflow-evaluated or implemented. Figure 2 was revised to use the same stage definitions and is presented as a qualitative evidence-stage map rather than a maturity ranking. Sections 4.4 and 7 now distinguish prognostic risk prediction from treatment-effect estimation and clearly label agentic systems, foundation models, and individualised treatment selection as forward-looking or currently unvalidated applications.

Location: Table 2; Figure 2; Sections 4.4 and 7;Pending issues.

Referee #2 – Comment 3: Expand discussion of foundation models

> *The section on large language models is interesting but relatively brief. Given the rapid emergence of multimodal foundation models capable of jointly processing imaging and text, the authors could discuss how these models differ from conventional supervised machine learning, their potential advantages, and their current limitations in neuroimmunology.*

Response: We expanded Section 7.3 to distinguish foundation models from conventional task-specific supervised learning. The revised text explains their pretrain-and-adapt framework, potential advantages for rare diseases and multimodal data integration, and current limitations, including domain shift, limited disease-specific representation, hallucination, calibration, benchmark availability, computational burden, and governance requirements. We also clarify that these systems remain forward-looking in CNS autoimmune diseases, with no prospective clinical validation identified to date.

Changes in the manuscript: Section 3.2 now briefly contrasts conventional supervised learning with the pretrain-and-adapt paradigm of foundation models. Section 7.3 was expanded to discuss multimodal vision-language models, their potential use for integrating imaging, clinical text, biomarkers, treatment exposure, and longitudinal outcomes, and their current limitations. Table 2 and Figure 2 classify these applications as early empirical or forward-looking rather than clinically validated. No prospective clinical validation of foundation models or medical LLMs in CNS autoimmune diseases was identified.

Location: Section 3.2; Section 7.3; Table 2; Figure 2.

Referee #2 – Comment 4: Address regulatory and implementation challenges in greater depth

> *Clinical deployment depends not only on model performance but also on regulatory approval, workflow integration, reimbursement, medico-legal responsibility, cybersecurity, and continuous post-deployment monitoring. These practical barriers deserve additional discussion.*

Response: We expanded the implementation discussion to cover risk-based regulatory requirements, local and prospective validation, workflow integration, interoperability, clinician oversight, liability, reimbursement, cybersecurity, and post-deployment monitoring. We also added staged clinical-impact evaluation and described procedures for detecting model drift, recalibration, rollback, and controlled updating. The revised text distinguishes technical performance from clinical utility and implementation readiness, and notes relevant regulatory frameworks in the United States, European Union, and China.

Changes in the manuscript: Sections 7.6 and 7.7 now discuss staged clinical-impact evaluation and the regulatory and implementation requirements for CNS autoimmune disease AI. The revised text addresses local validation, workflow integration, interoperability, clinician oversight, liability, reimbursement, cybersecurity, post-deployment surveillance, model drift, recalibration, rollback, and lifecycle governance. Figure 3 was added to summarise the staged pathway from model development to clinical implementation.

Location: Sections 7.6 and 7.7; Figure 3.

Referee #2 – Comment 5: Improve discussion of multimodal data fusion

> *Multimodal integration is a recurring theme, but the review could better explain practical challenges such as missing modalities, temporal alignment, heterogeneous data quality, harmonization across institutions, and uncertainty estimation.*

Response: We revised the multimodal modelling discussion to distinguish early, joint/intermediate, and late/decision fusion. The revised text and Table 1 now address missing and asynchronously acquired modalities, temporal alignment, unequal data quality, cross-centre and cross-scanner heterogeneity, modality dominance, harmonisation, temporal leakage, and uncertainty propagation. We also discuss practical approaches such as modality-specific encoders, imputation or masking, modality dropout, calibration, ablation testing, and uncertainty-aware fallback or abstention.

Changes in the manuscript: Section 3.1 and Table 1 were revised to define the main multimodal fusion strategies and their practical limitations. The revised discussion now covers missingness, asynchronous sampling, temporal

alignment, heterogeneous data quality, cross-centre harmonisation, dominant-modality effects, temporal leakage, and uncertainty-aware model use.

Location: Section 3.1; Table 1; related discussion of multimodal and longitudinal modelling in Sections 3.2–3.4.

Referee #2 – Comment 6: Include a stronger discussion of explainability and trustworthiness

> *While SHAP and Grad-CAM are introduced, it would be useful to emphasize that explainability methods alone do not establish model reliability. Calibration, uncertainty estimation, robustness testing, fairness analyses, and prospective validation should receive similar emphasis.*

Response: We revised Section 3.3 to present trustworthiness as a multidimensional property rather than an extension of interpretability. The revised framework covers calibration, uncertainty estimation, robustness, fairness, and external and prospective validation. SHAP and Grad-CAM are now described as post-hoc inspection tools and are not treated as evidence of model reliability or individualised clinical guidance. The disease-specific tables were also reviewed to ensure that these methods are reported descriptively and alongside relevant validation and performance limitations.

Changes in the manuscript: Section 3.3 now discusses interpretability, calibration, uncertainty, robustness, fairness, and external/prospective validation as separate components of trustworthy AI evaluation. SHAP and Grad-CAM language was moderated throughout the manuscript and tables. Tables 3–6 and Supplementary Tables S2–S5 now report calibration or uncertainty information where available and identify post-hoc attribution methods as descriptive rather than validating evidence.

Location: Section 3.3; Section 3.4; Tables 3–6; Supplementary Tables S2–S5; Glossary; disease-specific sections.

Referee #2 – Comment 7: Provide a clearer roadmap for future research

> *The review summarizes many developments but would benefit from a concluding figure outlining key priorities over the next 5-10 years, including multicenter prospective validation, federated learning, standardized datasets, multimodal foundation models, and clinical implementation studies.*

Response: We added Figure 3 as a staged roadmap for the next 5–10 years of research and translation in CNS autoimmune disease AI. The roadmap is organised into near-term (0–3 years), mid-term (3–5 years), and long-term (5–10 years) priorities. It covers multicentre data infrastructure, trustworthy evaluation, multimodal integration, foundation models, prospective clinical validation, and regulatory and implementation pathways, including federated learning, standardised benchmarking, clinical-impact studies, reimbursement, and post-deployment monitoring.

Changes in the manuscript: Figure 3 and its accompanying text now present a staged 5–10-year roadmap covering data infrastructure, trustworthy evaluation, multimodal and federated approaches, foundation models, prospective clinical-impact studies, and regulatory and implementation requirements. The roadmap distinguishes near-term retrospective and multicentre validation from mid-term prospective workflow evaluation and longer-term interventional studies and clinical deployment.

Location: Figure 3; Sections 7.1 and 7.3–7.7.

Referee #2 – Minor editorial comment 1: Terminology

> *Ensure consistent terminology throughout the manuscript when referring to AI, machine learning, and deep learning.*

Response: We introduced an explicit methodological taxonomy in Section 3.1 and applied it throughout the manuscript. AI is used as an umbrella term covering machine-learning, deep-learning, and generative or foundation-model approaches. Traditional statistical models, causal-inference methods, and standard bioinformatic workflows are described separately as complementary or AI-adjacent methods rather than AI per se. We also removed the non-standard abbreviation "CNS-AID" and harmonised terminology across the text, figures, and tables.

Changes in the manuscript: A methodological taxonomy was added in Section 3.1 and summarised in Box 1. Terminology was standardised throughout the manuscript, and statistical, causal-inference, and bioinformatic methods are now distinguished from AI methods.

Location: Section 3.1; Box 1; throughout the manuscript.

Referee #2 – Minor editorial comment 2: Table density

> *Some tables are very dense and could be simplified to improve readability.*

Response: We simplified Tables 3–6 to retain the most decision-relevant information: study and application, cohort and unit of analysis, design and validation, performance and comparator, and key limitation and interpretation. Detailed study characteristics and extended methodological information are now provided in Supplementary Tables S2–S5.

Changes in the manuscript: The main evidence tables were simplified and detailed extraction was moved to supplementary material.

Location: Tables 3–6; Supplementary Tables S2–S5.

Referee #2 – Minor editorial comment 3: Repetition

> *Consider reducing repetition regarding external validation and reporting guidelines across multiple sections.*

Response: We consolidated the general discussion of external validation, bias, trustworthiness, and reporting standards in Section 3.4. The disease-specific sections now focus on disorder-specific limitations rather than repeating the same generic methodological cautions, and Section 7 focuses on prospective infrastructure and implementation priorities rather than restating the validation gap.

Changes in the manuscript: General discussion of external validation, bias, trustworthiness, and reporting standards was consolidated in Section 3.4. Repetitive methodological cautions were reduced in the disease-specific sections, while Section 7 was refocused on prospective infrastructure, benchmarking, clinical-impact evaluation, and implementation priorities.

Location: Section 3.4; disease-specific Sections 6.1–6.5; Section 7.

Response to Referee #3

General assessment

> *This review summarizes current and emerging applications of AI in CNS autoimmune diseases, highlighting recent clinical advances and the challenges to implementing precision neuroimmunology.*

Referee #3 – Comment 1: Disease-specific framework in the Introduction

> *The Introduction would benefit from a concise disease-specific framework linking biological heterogeneity, available biomarkers, and unresolved clinical decision points to the AI applications discussed in the subsequent sections.*

Response: We added a disease-specific framework at the end of the Introduction that summarises the main clinical uncertainties and unresolved decision points in MS, NMOSD, MOGAD, and AE, and explains why these problems require multimodal and longitudinal data integration. Figure 1 was revised to connect these disease-specific needs with the imaging, biomarker, clinical, and computational approaches discussed later in the Review.

Changes in the manuscript: A disease-specific framework was added to the end of the Introduction, linking the main uncertainties and clinical decision points in MS, NMOSD, MOGAD, and AE to the multimodal AI approaches discussed in the Review. Figure 1 was revised to reflect these links.

Location: Introduction; Figure 1.

Referee #3 – Comment 2: Clarify review methodology

> *The manuscript combines features of a narrative and scoping review. The authors should clarify its intended methodology and, if applicable, briefly report the search strategy, eligibility criteria, study selection, and data-extraction process. Otherwise, claims implying comprehensive evidence coverage should be moderated.*

Response: We clarified that the manuscript is a structured narrative review informed by a documented literature search, rather than a formal systematic review, scoping review, or meta-analysis. A standalone "Review Scope and Evidence Identification" section now reports the databases and search period, the main selection priorities, the inclusion of contextual sources, and the study selection and extraction process. We also clarified that the synthesis is qualitative and does not represent an exhaustive catalogue of the literature.

Changes in the manuscript: A standalone "Review Scope and Evidence Identification" section was added between the Introduction and Section 2. The search strategy, study-selection process, evidence-extraction procedures, and qualitative scope of the review are now specified, and wording implying exhaustive evidence coverage was moderated.

Location: Review Scope and Evidence Identification, between the Introduction and Section 2.

Referee #3 – Comment 3: Mapping rather than maturity ranking

> *Given the limited evidence base in NMOSD, MOGAD, and AE, the manuscript should be framed primarily as a mapping of emerging applications and unmet methodological needs rather than as a definitive assessment of translational maturity.*

Response: We agree that the available evidence, particularly in NMOSD, MOGAD, and AE, does not support a definitive ranking of translational maturity. We therefore reframed the Review as a structured mapping of reported applications, evidence patterns, validation settings, and methodological needs. The revised manuscript uses a qualitative four-stage evidence framework in Table 2 and Figure 2, while Section 6.5 and Table 7 provide descriptive cross-disease mapping. The text now makes clear that these stages do not represent overall evidence quality, clinical readiness, or demonstrated patient benefit.

Changes in the manuscript: Language implying a formal ranking of translational maturity or clinical readiness was replaced with mapping-oriented and evidence-specific wording throughout the manuscript. Table 2 and Figure 2 now use a qualitative evidence-stage framework, while Section 6.5 and Table 7 present descriptive cross-disease evidence mapping rather than formal maturity rankings. The Abstract, Introduction, and Section 7 were also revised to avoid implying established clinical readiness or benefit.

Location: Abstract; Introduction; Table 2; Section 6.5; Table 7; Figure 2; Section 7.

Referee #3 – Comment 4: Clarify the definition of AI

> *The definition of AI is overly broad and includes conventional statistical methods, causal-inference approaches, and standard bioinformatic pipelines. A clearer taxonomy is needed to distinguish these methods and define which components are genuinely AI-based.*

Response: We revised Section 3.1 and Box 1 to define the methodological scope of AI more clearly. In this Review, AI is used as an umbrella term covering machine-learning, deep-learning, and generative or foundation-model approaches. Traditional statistical models, causal-inference methods, and standard bioinformatic workflows are described separately as complementary or AI-adjacent methods rather than AI per se. This distinction has been applied to the terminology used in the clinical, molecular, and disease-specific sections, tables, and figures.

Changes in the manuscript: A methodological taxonomy was added in Section 3.1 and summarised in Box 1. Method labels were standardised throughout the Review to distinguish AI methods from traditional statistical, causal-inference, and bioinformatic approaches, including in the disease-specific sections and evidence tables.

Location: Section 3.1; Box 1; Sections 4–6; Tables 1 and 3–7.

Referee #3 – Comment 5: Improve Table 1 multimodal fusion

> *In Table 1, "multimodal fusion" is too broadly defined and provides limited methodological or clinical information. The authors should specify the main fusion strategies, the types and timing of data being integrated, and representative disease-specific applications.*

Response: We revised Table 1 to distinguish early, joint/intermediate, and late/decision fusion, and added the corresponding computational approaches, clinical roles, and integration limitations. Section 3.1 now also explains how asynchronous data collection, missing modalities, unequal data quality, temporal alignment, and cross-centre heterogeneity affect the choice and evaluation of fusion strategies.

Changes in the manuscript: Table 1 was restructured to present three explicit multimodal fusion strategies instead of a single generic row. The accompanying text in Section 3.1 was expanded to address timing, availability, missingness, and heterogeneity of the integrated data.

Location: Section 3.1; Table 1.

Referee #3 – Comment 6: Practical challenges of multimodal and longitudinal modelling

> *The discussion of multimodal and longitudinal modelling should address practical challenges, including missing modalities, asynchronous measurements, temporal leakage, treatment exposure, model drift, and cross-center transportability.*

Response: We expanded the multimodal and longitudinal modelling discussion to address missing and informative missingness, asynchronous measurements, clinical temporal alignment, temporal leakage, treatment exposure, scanner/assay/site heterogeneity, uncertainty, and cross-centre transportability. The revised text also discusses model drift and lifecycle monitoring, including post-deployment surveillance, recalibration, rollback, and controlled updating.

Changes in the manuscript: The multimodal and longitudinal modelling sections were expanded to cover missing modalities, asynchronous sampling, temporal alignment, temporal leakage, treatment exposure, cross-centre heterogeneity, transportability, and uncertainty. Model drift and lifecycle monitoring were incorporated into the implementation discussion.

Location: Sections 3.1–3.4; Section 4.3; Sections 7.1 and 7.7.

Referee #3 – Comment 7: Apply SHAP/Grad-CAM caution consistently

> *The limitations of SHAP and Grad-CAM are appropriately acknowledged in Section 3.3; however, this caution should be applied consistently throughout the disease-specific sections and tables, where explainability methods are occasionally used as indicators of methodological strength or individualized clinical guidance.*

Response: We reviewed the disease-specific sections and tables and revised the wording so that SHAP and Grad-CAM are presented only as post-hoc inspection methods. They are not treated as evidence of model reliability, validated interpretability, or individualised clinical guidance. The revised text and tables also note that the stability and clinical validity of such attributions require independent evaluation.

Changes in the manuscript: SHAP and Grad-CAM descriptions were standardised throughout the manuscript and tables. Post-hoc attribution is now reported descriptively and is not presented as a methodological strength, proof of clinical validity, or basis for individualised guidance.

Location: Section 3.3; Section 6.3; Table 7; Supplementary Table S4; Glossary.

Referee #3 – Comment 8: Apply methodological standards to individual studies

> *Although Section 3.4 appropriately outlines general methodological limitations and reporting standards, these criteria are not consistently applied to the individual studies summarized in the disease-specific sections and tables. The review would therefore benefit from a more critical appraisal of geographical, demographic, age-related, and healthcare-system biases, as well as negative findings, failed validations, and publication bias, in order to avoid an overly optimistic representation of current AI performance, particularly in rare diseases and pediatric cohorts.*

Response: We agree that methodological standards should be applied at the study level, not only discussed in general terms. We therefore revised Tables 3‑6 to report cohort characteristics, unit of analysis, study design, validation strategy, performance measures, comparators, and key limitations. The revised Section 3.4 also discusses spectrum bias, data leakage, overfitting, annotation variability, limited external validation, and the underreporting of negative or failed validation results. In addition, Section 6.5 now highlights important cross-disease biases, including geographical concentration, paediatric representation, specialist-centre recruitment, and the underrepresentation of seronegative and diagnostically uncertain populations. Detailed study-level information is provided in Supplementary Tables S2–S5.

Changes in the manuscript: Tables 3–6 were standardised to include study-level design, validation, performance, comparator, and limitation information. Section 3.4 was expanded to address major sources of bias and selective reporting. Cross-disease demographic and geographical limitations were added to Section 6.5, and detailed study extraction was provided in Supplementary Tables S2–S5.

Location: Section 3.4; Section 6.5; Tables 3–6; Supplementary Tables S2–S5.

Referee #3 – Comment 9: Distinguish prognosis from treatment-effect estimation

> *The distinction between prognostic risk prediction and treatment-effect estimation is appropriately acknowledged in Section 4.4, but it should be applied consistently throughout the manuscript, particularly in Table 1 and in claims concerning "precision treatment recommendation" or treatment guidance.*

Response: We revised the manuscript to separate prognostic risk prediction from treatment-effect estimation. Prognostic models are now described as tools for risk stratification, monitoring, and care planning under observed treatment, rather than as treatment-selection tools. We also clarified that estimating comparative treatment effects requires a causal estimand, appropriate handling of treatment exposure and confounding, and prospective interventional validation. This distinction has been applied to Table 2 and the disease-specific sections.

Changes in the manuscript: Treatment-related language was revised throughout the manuscript. Table 2 now distinguishes risk-informed care planning from comparative treatment-effect estimation and individualised therapy selection, the latter being identified as an unmet conceptual application. Disease-specific sections were revised to ensure that prognostic and treatment-response models are not presented as evidence of individual treatment benefit.

Location: Sections 4.3–4.4; Section 5.3; Sections 6.1–6.5; Section 7; Tables 1–2; Pending issues.

Referee #3 – Comment 10: Separate agentic/foundation-model perspectives and clarify federated-learning evidence

> *The sections on agentic systems and foundation models are appropriately forward-looking but should remain clearly separated from clinically validated applications. For federated learning, the authors should distinguish demonstrated feasibility in other medical domains from direct disease-specific evidence in CNS autoimmunity.*

Response: We revised the manuscript to distinguish forward-looking systems from clinically validated applications. Agentic systems and foundation models are now explicitly presented as conceptual or early empirical approaches, with no prospective disease-specific validation identified in CNS autoimmunity. Federated learning is discussed separately: studies in adjacent medical fields are described as evidence of technical feasibility, whereas direct CNS autoimmune evidence is currently limited to research-stage studies in MS. No disease-specific federated-learning studies were identified for NMOSD, MOGAD, or AE.

Changes in the manuscript: The evidence status and clinical scope of agentic systems, foundation models, and federated learning were clarified. Table 2 now classifies agentic decision support as conceptual and foundation models as early empirical/forward-looking. Section 3.3 and Section 7.1 distinguish adjacent-domain feasibility from disease-specific evidence, while Sections 7.2–7.3 describe agentic and foundation-model applications as future research directions rather than established clinical tools.

Location: Section 3.3; Sections 7.1–7.3; Table 2; Figure 3; Pending issues.

Referee #3 – Comment 11: Clarify the relationship between molecular discovery and clinical decision support

> *The molecular-discovery sections and clinical decision-support address distinct questions and currently feel only loosely connected: their relationship should be clarified, or the two domains should be more clearly separated. Moreover, the molecular-discovery section should more clearly distinguish statistical association, causal prioritization, mechanistic hypothesis generation, experimental validation, druggability, and demonstrated therapeutic efficacy.*

Response: We revised the manuscript to distinguish patient-level clinical decision support from molecular discovery and translational research. Section 4 now focuses on clinical decision points, whereas Section 5 addresses molecular associations, causal prioritisation, mechanistic hypotheses, target evaluation, and translation. We also added a six-level molecular evidence ladder, ranging from statistical association to demonstrated therapeutic efficacy. Candidate targets are now described according to the evidence available, and computational prioritisation is not treated as evidence of mechanism, druggability, or clinical benefit. The link between the two domains is presented as an incomplete molecular-to-clinical translation loop.

Changes in the manuscript: Sections 4 and 5 were reorganised to separate clinical decision support from molecular discovery while clarifying their translational relationship. Section 5.1 now introduces a six-level evidence ladder, and Sections 5.2–5.3 clarify the requirements for moving from molecular findings to clinical stratification, trial enrichment, and therapeutic evaluation.

Location: Sections 4 and 5, particularly Sections 5.1–5.3.

Referee #3 – Comment 12: Standardise Tables 3–6

> *Tables 3-6 should be standardized to report sample size, unit of analysis, study design, validation strategy, performance metrics with confidence intervals, comparators, and main methodological limitations.*

Response: Tables 3–6 were revised to use a common structure covering cohort size, unit of analysis, study design, validation strategy, performance and comparator information, and key methodological limitations. Confidence intervals are reported when available, and missing information is marked as NR rather than inferred. Detailed study-level extraction, including input data, model type, reference standard, calibration or uncertainty reporting, and extended performance measures, is provided in Supplementary Tables S2–S5.

Changes in the manuscript: Tables 3–6 were standardised to include study design, validation, performance, comparator, and limitation information. Detailed study extraction was added to Supplementary Tables S2–S5.

Location: Tables 3–6; Supplementary Tables S2–S5.

Referee #3 – Comment 13: Clarify Figure 2 evidence criteria

> *The translational-maturity criteria used in Figure 2 would benefit from further clarification. As each domain appears to be classified according to the highest stage reached by a cited study, the figure may not fully reflect the volume, consistency, or quality of the supporting evidence. The authors could therefore link each classification to specific studies and clearer criteria, or present the figure more explicitly as a qualitative overview.*

Response: We revised Figure 2 so that it presents a qualitative evidence-stage map rather than a formal maturity ranking. Its stage definitions are now aligned with Table 2: conceptual, early empirical, validated research, and workflow-evaluated or implemented. The revised caption also clarifies that each cell reflects the highest evidence stage identified for a disease–application pair and does not indicate the volume, consistency, quality, regulatory status, or clinical benefit of the supporting evidence.

Changes in the manuscript: Figure 2 was retitled and its caption was revised to define the four evidence stages and clarify the interpretive limitations of the map. Related text, including Section 6.5, was revised to describe the figure as a qualitative overview rather than a maturity ranking.

Location: Figure 2 and its caption; Table 2; Section 6.5.

Referee #3 – Comment 14: Moderate overstated wording

> *Claims such as "first", "highest", "state-of-the-art", "near-perfect", and "clinical deployment" should be carefully verified and replaced with more cautious wording when direct evidence is lacking.*

Response: We reviewed the manuscript for superlative and insufficiently supported language and replaced such wording with study-specific and evidence-appropriate descriptions. Claims implying that an approach was the first, highest-performing, state-of-the-art, near-perfect, or clinically deployed were removed or moderated unless directly supported by the cited evidence. We also clarified that “highest evidence stage” in Figure 2 refers only to the most advanced evidence category identified for a disease–application pair, not to performance, evidence quality, or clinical maturity.

Changes in the manuscript: Overstated and absolute claims were revised throughout the text, tables, and figure legends. Figure 2 and Table 7 were revised to use qualitative evidence-stage and study-specific descriptions, while statements concerning imaging performance, LLMs, federated learning, and clinical implementation were moderated.

Location: Throughout the manuscript, particularly Sections 3.3–3.4, 6.5 and 7; Tables 3 and 7; Figure 2 and its caption.

Referee #3 – Comment 15: Reduce redundancy

> *The manuscript is often redundant, and several relatively simple concepts could be presented more concisely. Substantial repetition is particularly evident across Sections 2, 4, 6, and 7, which should be condensed and shortened to improve clarity, focus, and ease of consultation.*

Response: We revised the manuscript to give each section a more distinct purpose and removed repeated explanations and summary statements. General discussion of bias, external validation, reporting standards, and trustworthiness is now concentrated in Section 3. Sections 2, 4, 6, and 7 were shortened and refocused on the clinical landscape, decision points, disease-specific evidence, and future research or implementation priorities, respectively.

Changes in the manuscript: Repeated methodological and summary content was removed or consolidated, and the structure of Sections 2–7 was refined to reduce overlap and improve readability.

Location: Sections 2–7, particularly Sections 2, 3, 4, 6, and 7.

Closing statement

We again sincerely thank the Editor and all three Reviewers for their careful, thoughtful, and constructive comments. We fully agree that the requested revisions have improved the manuscript substantially. In particular, the revised Review now provides a clearer methodological taxonomy, a more critical and study-specific appraisal of bias and validation, a more cautious evidence-stage framework, stronger separation between established and forward-looking applications, expanded discussion of multimodal integration and trustworthiness, a clearer molecular evidence hierarchy, and a more practical roadmap for future clinical translation. We hope that the revised manuscript satisfactorily addresses all of the concerns raised and will be suitable for publication in EMBO Molecular Medicine.

9th Sep 2026

Dear Prof. Shi,

We are pleased to inform you that your manuscript is accepted for publication and is now being sent to our publisher to be included in the next available issue of EMBO Molecular Medicine.

Your manuscript will be processed for publication by EMBO Press. It will be copy edited and you will receive page proofs prior to publication. Please note that you will be contacted by Springer Nature Author Services to complete licensing information.

Should you be planning a Press Release on your article, please notify the production team via this form in order to coordinate publication and release dates: https://forms.cloud.microsoft/e/eH4A8JVP6g

If you have any questions, please do not hesitate to contact the Editorial Office. Thank you for your contribution to EMBO Molecular Medicine.

Yours sincerely,
Zeljko Durdevic

Zeljko Durdevic
Senior Editor
EMBO Molecular Medicine
