# Supplementary material for: Artificial intelligence in central nervous system autoimmune diseases: from clinical decision support to mechanistic discovery and translation
Source: EMBO Mol Med. 2026 Oct 13;18(11):4179–99. doi: 10.1038/s44321-026-00527-0 (PMC13748597; doi:10.1038/s44321-026-00527-0)
Supplement: Supplementary file 2 [file 44321_2026_527_MOESM1_ESM.docx]

Table EV1. Reporting guidelines and checklists relevant to medical AI studies.

| Guideline / checklist | Primary study scope | Key reporting focus | Relevance to this Review | Citation |
| --- | --- | --- | --- | --- |
| TRIPOD+AI | Development, validation, or updating of clinical prediction models using regression or machine-learning methods | Participants, predictors, outcomes, model development, validation, performance, fairness, transparency, and reproducibility | Prognostic, relapse-risk, disability, cognitive-outcome, and other patient-level prediction models | Collins et al, 2024 |
| CLAIM | Medical imaging AI studies | Imaging datasets, reference standards, preprocessing, annotation, model development, data partitioning, performance evaluation, and external validation | MRI-, PET-, OCT-, fundus-, and radiomics-based studies | Mongan et al, 2020 |
| STARD-AI | Diagnostic-accuracy studies evaluating AI-based index tests | Dataset practices, AI index-test specification, reference standards, evaluation design, algorithmic bias, fairness, applicability, and generalisability | AI-based differential diagnosis and disease-classification studies | Sounderajah et al, 2025 |
| CONSORT-AI | Randomised clinical trials evaluating AI interventions | AI intervention description, input/output handling, human–AI interaction, errors, and trial-specific implementation | Future interventional evaluation of AI-assisted clinical decision-making | Liu et al, 2020 |
| SPIRIT-AI | Protocols for randomised trials of AI interventions | Prespecification of AI version, input requirements, output use, human interaction, and pretrial evaluation | Protocol development for future AI clinical-impact trials | Cruz Rivera et al, 2020 |
| DECIDE-AI | Early-stage live clinical evaluation of AI-based decision-support systems | Clinical workflow integration, human–AI interaction, usability, safety, failure modes, and prospective real-world evaluation | Prospective workflow studies before definitive comparative trials | Vasey et al, 2022 |
| MI-CLAIM | Minimum reporting framework for clinical AI model development | Data provenance, model development, performance evaluation, reproducibility, and transparency | Complementary checklist for early clinical AI model-development studies | Norgeot et al, 2020 |

**Note.** The appropriate reporting framework depends on study design and intended use. Prediction-model, diagnostic-accuracy, imaging, early live clinical evaluation, and interventional studies require different reporting standards; no single checklist is sufficient for all AI studies. These frameworks address reporting completeness and should not be interpreted as substitutes for formal risk-of-bias assessment or evidence of clinical validity.

**Abbreviations:** AI, artificial intelligence; CLAIM, Checklist for Artificial Intelligence in Medical Imaging; CONSORT-AI, Consolidated Standards of Reporting Trials–Artificial Intelligence; DECIDE-AI, Developmental and Exploratory Clinical Investigations of Decision support systems driven by Artificial Intelligence; MI-CLAIM, Minimum Information about Clinical Artificial Intelligence Modeling; SPIRIT-AI, Standard Protocol Items: Recommendations for Interventional Trials–Artificial Intelligence; STARD-AI, Standards for Reporting Diagnostic Accuracy Studies–Artificial Intelligence; TRIPOD+AI, Transparent Reporting of a multivariable prediction model for Individual Prognosis Or Diagnosis–Artificial Intelligence.
