# Supplementary material for: Artificial intelligence in central nervous system autoimmune diseases: from clinical decision support to mechanistic discovery and translation
Source: EMBO Mol Med. 2026 Oct 13;18(11):4179–99. doi: 10.1038/s44321-026-00527-0 (PMC13748597; doi:10.1038/s44321-026-00527-0)
Supplement: Supplementary file 3 [file 44321_2026_527_MOESM2_ESM.docx]

Table EV2. Detailed characteristics, validation strategies, and reported performance of representative AI studies in multiple sclerosis.

| Study | Study type | Clinical task | Cohort and setting | Unit of analysis | Input data | Model / approach | Development and validation | Reference standard / comparator | Main reported performance | Calibration / uncertainty reporting | Key limitation |
| --- | --- | --- | --- | --- | --- | --- | --- | --- | --- | --- | --- |
| Gabr et al, 2020 | Original study | MS lesion and tissue segmentation | 1,003 baseline MRI datasets from RRMS participants in the multicentre CombiRx phase 3 trial; 68 centres | MRI dataset / lesion level | Multisequence brain MRI | FCNN/U-Net | Random 60%/20%/20% train/validation/test split plus leave-one-centre-out cross-validation; all data from CombiRx | Expert/reference segmentations | Test Dice: WM 0.95, GM 0.96, CSF 0.99, T2 lesions 0.82; leave-one-centre-out T2-lesion Dice 0.85 | 95% CIs reported for major Dice estimates; calibration not applicable | Large multicentre dataset, but validation remained within one trial protocol and may not represent routine heterogeneous MRI |
| Coronado et al, 2021 | Original study | Contrast-enhancing lesion segmentation | 1,006 RRMS patients from CombiRx; 398 with ≥1 gadolinium-enhancing lesion | Patient/MRI dataset and lesion level | Contrast-enhanced MRI | 3D U-Net CNN | Stratified 60%/20%/20% train/validation/test split within CombiRx; no geographically external cohort | Expert-validated lesion segmentations | U5 model DSC/TPR/FPR 0.77/0.90/0.23 overall; for lesions >500 mm³, 0.81/0.97/0.04 | 95% CIs NR; calibration not applicable | Performance declined markedly for very small lesions; validation remained within the CombiRx environment |
| Wiltgen et al, 2024 | Original study | Lesion segmentation with external benchmarking | 491 development cases; external MSSEG-1 dataset | MRI scan / lesion level | Brain MRI | 3D U-Net ensemble (LST-AI) | Development cohort plus external benchmark testing on MSSEG-1 | LST, SAMSEG, nnU-Net and benchmark reference labels | MSSEG-1 Dice 0.65; detection >75% for lesions >10 mm³ | CI/calibration NR | External benchmark strengthens reproducibility evidence, but lesion-size dependence and absence of prospective workflow impact remain limitations |
| Barnett et al, 2023 | Original study | Longitudinal disease-activity monitoring | 397 paired MRI examinations in a multicentre prospective workflow study | Paired longitudinal MRI examination / patient level | Serial clinical brain MRI | Automated volumetric/disease-activity assessment | Prospective multicentre workflow evaluation | Conventional radiology reporting and core-laboratory volumetry | Sensitivity 93.3% versus 58.3% for conventional reporting; volumetry comparable with core-laboratory measurement | CI NR; calibration not applicable to reported detection/volumetry endpoints | Prospective workflow evidence is strong, but effects on treatment decisions, workload and patient outcomes were not established |
| Bouman et al, 2023 | Original study | Synthetic MRI for cortical-lesion detection | 202 patients from seven centres | Patient / lesion / reader assessment | MRI-derived synthetic DIR and PSIR | AI-generated synthetic contrasts | Multicentre observational evaluation with seven blinded expert readers | Conventional imaging and expert lesion assessment | AI-DIR detected 1,154 versus 855 lesions; AI-PSIR 803 versus 814; reader ICC 0.76 for AI-DIR and 0.85 for AI-PSIR | 95% CIs reported for ICC; calibration not applicable | Multicentre recruitment is a strength, but the study assessed lesion readout rather than full diagnostic accuracy or downstream clinical benefit |
| Chen et al, 2024 | Original study | Automated PRL detection | 92 participants; 5,637 white-matter lesions including 148 expert-labelled PRLs | Lesion and subject level | 3-T susceptibility-sensitive MRI | Automated PRL detection model | Multicentre validation against expert-consensus labels | Expert PRL consensus | 115/148 PRLs detected (78%); lesion-level AUC 0.73; subject-level AUC 0.69 | 95% CIs: lesion AUC 0.68–0.78; subject AUC 0.57–0.81 | Moderate discrimination, false positives/missed lesions, specialised acquisition, and expert-label dependence limit routine use |
| Manning et al, 2025 | Original study | Automated CVS assessment | 86 participants evaluated for MS across nine sites; automated processing completed in 79 | Patient / lesion-marker level | Susceptibility-sensitive MRI | Automated CVS assessment | Multicentre diagnostic evaluation | Simplified manual CVS counting and full manual CVS proportion | Automated AUC 0.78; simplified manual 0.80; full manual 0.89 | Automated AUC 95% CI 0.67–0.88; calibration NR | Motion-related processing failure, specialised acquisition and relatively small diagnostically evaluated cohort limit generalisability |
| Storelli et al, 2022 | Original study | Two-year disability and cognitive progression | 373 MS patients; development/optimisation in 325 patients from four centres and independent 48-patient test cohort from another centre/scanner | Patient level | Baseline T1- and T2-weighted 3-T MRI | Deep-learning CNN prognostic model | Multicentre development with independent test dataset acquired on a different scanner | Two-year EDSS/SDMT-related clinical outcomes; expert physicians | Clinical worsening accuracy 83.3%; cognitive worsening 67.7%; joint EDSS+SDMT 85.7%; physician accuracy 70.0% | 95% CIs NR; calibration NR | Independent testing is valuable, but the external cohort was small and treatment exposure and calibration were insufficiently addressed |
| Ferrè et al, 2023 | Original study | Fingolimod treatment-response prediction | 381 treated RRMS patients; 197 EDA and 184 NEDA; train/validation/test sets 152/152/77 from Italian and French cohorts | Patient level | Clinical variables and genetic variants | Random forest | Separate training, feature/signature-selection validation, and independent test sets | NEDA-3 outcome; clinical-only and genetic-only models | Independent test: combined AUC 0.71/AUPRC 0.73; clinical-only AUC 0.69; genetic-only AUC 0.65 | 95% CIs NR; calibration NR | Modest discrimination and small independent test set; observed treatment-response prediction does not establish individual treatment effect or optimal therapy selection |
| Delgado Hernández et al, 2026 | Original study | Voice-based digital biomarker | 300 participants: 200 MS and 100 neurologically healthy controls; independent clinical validation cohort n=100 | Participant level | Sustained-vowel acoustic recordings; 15 features | Elastic-net feature selection plus five supervised classifiers; best model random forest | Development n=200 with random split and cross-validation; independent clinical validation n=100 | Diagnostic group labels; clinical-reader comparator NR | Independent validation AUC 0.85, accuracy 0.85, balanced accuracy 0.80, sensitivity 0.80, specificity 0.80 | AUC 95% CI 0.76–0.93; accuracy CI reported; calibration NR | Healthy-control comparison, language, recording environment and device effects limit differential-diagnostic transportability |

**Note.** Study characteristics, validation strategies, and performance estimates are reported as described in the cited publications. NR indicates not reported. “External validation” refers to evaluation in an independently sampled cohort, site, or benchmark dataset distinct from the model-development data. Calibration is reported only where applicable to probabilistic prediction models. The final column provides a narrative design-based appraisal rather than a formal risk-of-bias assessment.

**Abbreviations:** AUC, area under the receiver-operating characteristic curve; AUPRC, area under the precision–recall curve; CI, confidence interval; CSF, cerebrospinal fluid; CVS, central vein sign; DIR, double inversion recovery; DSC, Dice similarity coefficient; EDA, evidence of disease activity; EDSS, Expanded Disability Status Scale; FCNN, fully convolutional neural network; GM, grey matter; MRI, magnetic resonance imaging; MS, multiple sclerosis; NEDA, no evidence of disease activity; NR, not reported; PRL, paramagnetic rim lesion; PSIR, phase-sensitive inversion recovery; RRMS, relapsing-remitting multiple sclerosis; SDMT, Symbol Digit Modalities Test; TPR, true-positive rate; WM, white matter.
