# Supplementary material for: Artificial intelligence in central nervous system autoimmune diseases: from clinical decision support to mechanistic discovery and translation
Source: EMBO Mol Med. 2026 Oct 13;18(11):4179–99. doi: 10.1038/s44321-026-00527-0 (PMC13748597; doi:10.1038/s44321-026-00527-0)
Supplement: Supplementary file 4 [file 44321_2026_527_MOESM3_ESM.docx]

Table EV3. Detailed characteristics, validation strategies, and reported performance of studies relevant to AI applications in neuromyelitis optica spectrum disorder.

| Study | Study type | Clinical task | Cohort and setting | Unit of analysis | Input data | Model / approach | Development and validation | Reference standard / comparator | Main reported performance | Calibration / uncertainty reporting | Key limitation |
| --- | --- | --- | --- | --- | --- | --- | --- | --- | --- | --- | --- |
| Etemadifar et al, 2024 | Systematic review and meta-analysis | MRI-based NMOSD-versus-MS classification | 15 studies; sample sizes 53–351; total 1,362 MS and 1,118 NMOSD participants; 94.9% AQP4-IgG-positive across nine reporting studies | Study-level pooled evidence | MRI | Eight ML and seven DL studies | PROSPERO-registered systematic review/meta-analysis | Diagnostic labels reported in included studies | Pooled accuracy 82% (95% CI 78–86), sensitivity 83% (79–88), specificity 80% (75–86); MRI-only subgroup accuracy 83% | Pooled 95% CIs reported; calibration not synthesised | Useful field-level benchmark, but substantial heterogeneity in acquisition, model evaluation, validation strategy, and reporting limits inference about clinical transportability |
| Kim et al, 2020 | Original retrospective diagnostic study | NMOSD-versus-MS differentiation | 338 patients: 213 MS and 125 AQP4-IgG-positive NMOSD; single Korean centre | Patient level | FLAIR MRI plus basic clinical information | 3D CNN | Stratified 45%/15%/40% training/validation/test split; no external validation | Two board-certified neurologists | Reader comparator: rater A accuracy 65.9% (95% CI 57.3–73.9), rater B 60.7% (52.0–69.0); Exact model performance metric NR | Model calibration NR | Single-country, single-centre retrospective cohort with exclusively AQP4-IgG-positive NMOSD limits generalisability to seronegative and diagnostically ambiguous populations |
| Hagiwara et al, 2021 | Original retrospective diagnostic study | NMOSD-versus-MS classification using quantitative MRI | 53 patients: 35 RRMS and 18 AQP4-IgG-positive NMOSD | Patient level | Synthetic MRI-derived R1, R2, and proton-density maps | SqueezeNet-based compact CNN | Leave-one-out cross-validation across all 53 cases; no independent external test set | Clinical diagnosis; comparator model without common-feature selection | AUC 0.859; accuracy 81.1%; sensitivity 80.0% for MS and 83.3% for NMOSD; comparator AUC 0.572 | CI/calibration NR | Very small cohort, no external validation, and dependence on specialised quantitative MRI limit robustness and scanner-level transportability |
| He et al, 2022 | Original multicentre diagnostic study | MRI differentiation of NMOSD and MS | 223 patients from four centres for development/testing plus 74 external cases from three centres | Patient level | T2-weighted MRI radiomics | Random forest after radiomic feature selection and harmonisation | Multicentre development with geographically external validation | Clinical diagnosis | AUC 1.000/0.944/0.902 in training/internal test/external validation; external sensitivity 79.7%, specificity 86.0% | Calibration NR; geographically external validation reported | Strong external validation, but retrospective selection, scanner/protocol heterogeneity, treatment-related effects, and absent calibration reporting remain important limitations |
| Huang et al, 2022 | Original retrospective multiclass diagnostic study | Differentiation of MS, NMOSD, and MOGAD | 290 patients: 67 MS, 162 AQP4-IgG-positive NMOSD, 61 MOGAD | Patient level | Brain and/or spinal-cord MRI | MIL-CoaT Transformer | Single-centre 4:1 development/test split; no geographically external validation | Two neuroradiologists | NMOSD AUC 0.942 (95% CI 0.879–0.987); multiclass accuracy 81.4% versus 64.4% and 74.6% for radiologists | 95% CIs reported for disease-specific AUCs; calibration NR | Strong internal discrimination, but single-centre design, variable disease-specific performance, and lack of testing in consecutive diagnostically uncertain patients raise spectrum and transportability concerns |
| Cacciaguerra et al, 2022 | Original diagnostic/subgroup study | Classification of seronegative NMOSD and NMOSD-like presentations | 228 MRI examinations: 85 AQP4-IgG-positive NMOSD, 95 MS, 11 seronegative NMOSD, and 37 seronegative NMOSD-like cases | Patient/MRI level | Brain MRI | Four-layer 3D CNN | Model developed on AQP4-IgG-positive NMOSD versus MS and applied to seronegative and NMOSD-like cases | Clinical diagnostic categories | Accuracy 0.95 for AQP4-IgG-positive NMOSD versus MS; all 11 seronegative NMOSD and 36/37 NMOSD-like cases classified as NMOSD | CI/calibration NR | Clinically important seronegative extension, but very small seronegative/MOG-positive subgroups and uncertain diagnostic labels preclude treating these findings as robust external validation |
| Wang et al, 2022 | Original retrospective multicentre prognostic study | Relapse-risk prediction | 1,135 treatment episodes from 358 Huashan patients for development; 213 episodes from 92 patients across nine centres for validation | Treatment-episode level | Demographic, serological, disease-history, and treatment variables | Cox-PH, RSF, LogisticHazard, DeepHit, DeepSurv | Multicentre development and external-centre validation | Subsequent relapse | Validation C-index: LogisticHazard 0.718, DeepHit 0.704, DeepSurv 0.698, RSF 0.685, Cox-PH 0.651 | Calibration metrics NR in current extraction | Multicentre validation is a strength, but repeated treatment episodes, retrospective treatment exposure, dosing variation, and lack of prospective validation limit clinical use; treatment-related predictor importance does not establish treatment effect |
| El Khoury et al, 2022 | Original proof-of-concept diagnostic study | Serum spectroscopy classification | 235 serum samples: 80 healthy controls, 60 NMOSD, 60 RRMS, and 35 peripheral-neuropathy controls | Serum-sample level | FTIR serum spectra | Random forest | Two-fold cross-validation repeated 10 times plus an isolated 27-sample validation set; no independent external cohort | Diagnostic group labels | NMOSD cross-validation AUC 98.7 ± 0.9%; RRMS AUC 100 ± 0.0%; perfect classification reported in the small held-out four-class set | External calibration NR | Small disease subgroups, case–control sampling, limited serostatus representation, and absence of external spectrometer/site validation make the very high internal performance unsuitable as evidence of clinical readiness |
| Gu et al, 2025 | Original multimodal diagnostic study | NMOSD-related classification from fundus imaging | 1,618 UWF images from 1,055 participants: 285 NMO and 770 non-NMO; single-centre Chinese hospital | Image level with subject-ID grouping | Ultra-widefield fundus images plus clinical reports | Multimodal AI combining imaging and clinical information | Subject-grouped 80%/10%/10% train/validation/test split with five-fold cross-validation; no external cohort | Specialist, attending, and resident comparisons | Multimodal AUC 0.9923, sensitivity 97.0%, specificity 96.9%; UWF-only AUC 0.9751; AI performance compared with clinicians | CI/calibration NR in current extraction | High internal performance, but single-centre sampling, restricted case spectrum, multiple images per participant, lack of external validation, and limited progression/recurrence assessment constrain clinical utility. |

**Note.** Study characteristics, validation strategies, and performance estimates are reported as described in the cited publications. NR indicates not reported. “External validation” refers to evaluation in an independently sampled cohort or site distinct from the model-development data; application of a developed model to a small predefined subgroup is described separately and is not automatically considered external validation. Calibration is reported only where applicable to probabilistic prediction models. The final column provides a narrative design-based appraisal rather than a formal risk-of-bias assessment.

**Abbreviations:** AUC, area under the receiver-operating characteristic curve; AQP4-IgG, aquaporin-4 immunoglobulin G; CI, confidence interval; CNN, convolutional neural network; DL, deep learning; FTIR, Fourier-transform infrared spectroscopy; ML, machine learning; MOGAD, myelin oligodendrocyte glycoprotein antibody-associated disease; MRI, magnetic resonance imaging; MS, multiple sclerosis; NMOSD, neuromyelitis optica spectrum disorder; NR, not reported; RRMS, relapsing-remitting multiple sclerosis; RSF, random survival forest; UWF, ultra-widefield.
