# Supplementary material for: Artificial intelligence in central nervous system autoimmune diseases: from clinical decision support to mechanistic discovery and translation
Source: EMBO Mol Med. 2026 Oct 13;18(11):4179–99. doi: 10.1038/s44321-026-00527-0 (PMC13748597; doi:10.1038/s44321-026-00527-0)
Supplement: Supplementary file 5 [file 44321_2026_527_MOESM4_ESM.docx]

Table EV4. Detailed characteristics, validation strategies, and reported performance of studies relevant to AI and computational modelling in autoimmune encephalitis.

| Study | Study type | Clinical task | Cohort and setting | Unit of analysis | Input data | Model / approach | Development and validation | Reference standard / comparator | Main reported performance | Calibration / uncertainty reporting | Key limitation |
| --- | --- | --- | --- | --- | --- | --- | --- | --- | --- | --- | --- |
| Xiang et al, 2022b | Original retrospective DL diagnostic study | MRI differentiation of AE, HSVE, and healthy controls | Modelling cohort: 160 AE, 177 HSVE, 184 healthy controls; 52 additional patients from another site for external validation | Patient level | T1WI, T2WI, FLAIR, DWI and four-sequence fusion MRI | 3D ResNet18-based DL models | Internal development/test evaluation plus external-centre validation; radiologist reader experiment | Clinical diagnostic categories; radiologists | Internal fusion AUCs: AE 0.828, HSVE 0.884, HC 0.899; external AUCs: AE 0.831, HSVE 0.882, HC 0.892; AE identification accuracy 83% versus 72% mean radiologist accuracy | CI/calibration NR in current extraction | External validation and reader comparison are strengths, but inclusion of healthy controls and diagnostically resolved cases may introduce spectrum bias; performance in consecutive uncertain encephalopathy remains unknown |
| Shen et al, 2024 | Original ML diagnostic study | Detection of MRI-negative anti-NMDAR encephalitis | Training: 109 anti-NMDAR encephalitis, 108 healthy controls, 84 viral encephalitis; validation: 29, 28, and 26, respectively | Patient level | FreeSurfer-derived cortical and subcortical structural MRI features | Linear-kernel SVM | Independent temporal validation; no external-centre validation | Healthy controls and viral encephalitis | Validation AUC 0.963 versus healthy controls and 0.879 versus viral encephalitis; accuracy 93.0% and 78.2%, respectively | CI/calibration NR | Performance was lower against the clinically relevant viral-encephalitis comparator; scanner variability, demographic confounding, and lack of external-centre validation limit transportability |
| Petrosian et al, 2025 | Original retrospective ML diagnostic study | Infectious-versus-autoimmune/paraneoplastic encephalitis | 233 patients from a single centre: 83 autoimmune/paraneoplastic and 150 infectious encephalitis | Patient level | Clinical and laboratory variables | Random forest after feature selection and hyperparameter optimisation | Stratified 70%/30% train/test split plus five-fold cross-validation; no external validation | Six clinician comparators | Test AUC 0.966, accuracy 0.971, sensitivity 0.920, specificity 1.000, F1 0.958; clinician AUCs 0.769–0.896 | CI/calibration NR | Strong internal performance, but single-centre retrospective sampling, modest cohort size, class imbalance, limited seronegative AE representation, and no external validation constrain generalisability |
| Xiang et al, 2022a | Original multimodal prognostic study | Prognosis in anti-NMDAR encephalitis | 139 patients from one centre for development/internal testing plus 87 patients from a second centre for external testing | Patient level | Clinical variables plus multiparametric MRI DL and hippocampal radiomics features | Fusion nomogram integrating clinical, DL and radiomics predictors | Internal test set plus independent external-centre testing | Clinical outcome reference | Fusion-model AUC 0.963 internally and 0.927 externally; external accuracy 0.880, sensitivity 0.667, specificity 0.947 | Calibration assessed graphically; summary calibration statistic NR | External testing is a strength, but geographic concentration, manual hippocampal segmentation, treatment-related variables, and limited quantitative calibration reporting restrict wider transportability |
| Guo J et al, 2025 | Original ML prognostic study | Outcome prediction in antibody-positive AE | 121-patient training set and 67-patient validation set; abstract reports 187 patients despite 121+67 indicating 188 | Patient level | Clinical and laboratory variables | Nine ML algorithms after univariate and LASSO-based feature selection; RF best-performing model | Held-out validation set; discrimination, calibration, and clinical-applicability analyses | Clinical outcome | RF accuracy 0.860 and F1 0.844; XGBoost accuracy 0.826 and F1 0.807 | Calibration reported in validation set; detailed summary statistic NR | Small single-hospital cohort, no external-centre validation, and inconsistent reported sample total reduce confidence in reproducibility; post-hoc SHAP attribution does not establish explanatory validity |
| Guo & Zou, 2026 | Multi-omics ML / hypothesis-generating study | Prognostic biomarker discovery and mechanistic hypothesis generation | Antibody-positive AE patients with immunological indicators; mechanistic analyses supplemented by EAE single-cell/spatial data; PAIA evidence limited to one patient | Patient level plus molecular/cellular analyses | CSF/serum immunological measures, lymphocyte subsets, single-cell and spatial transcriptomic data | Eight ML algorithms with independent-cohort validation plus post-hoc attribution and multi-omics analyses | Independent-cohort validation; molecular evidence used for hypothesis support | Clinical outcome plus molecular associations | Immunological predictors identified; NPM1 nominated as a candidate target | Detailed discrimination and calibration statistics NR | Independent-cohort validation supports prognostic exploration, but PAIA evidence is limited to one patient and NPM1 remains a hypothesis-generating candidate requiring causal, safety, and druggability validation |
| Zhang Y et al, 2025 | Original multicentre ML prognostic study | Long-term cognitive impairment prediction | 341 adult AE patients from three tertiary hospitals; 264 development and 77 external-validation patients | Patient level | Clinical variables including CRQ, CASE, status epilepticus, age, MRI abnormalities, and treatment timing | Six ML algorithms; random forest selected as best model | Multicentre development with external validation | Long-term cognitive outcome | RF AUC 0.90; validation accuracy 0.87, sensitivity 0.79, specificity 0.90 | 95% CIs and detailed calibration statistics NR | Multicentre design is a strength, but retrospective construction, telephone-based cognitive assessment, and relatively small external-validation sample constrain clinical translation |
| Wu et al, 2022 | Statistical/regression modelling; AI-adjacent | Early ICU-admission risk prediction | 234 adult definite/probable AE patients from one centre; 40 admitted to ICU within 14 days | Patient level | Admission clinical and laboratory variables plus CASE or mRS | Logistic-regression risk models | Retrospective single-centre development with 1,000 bootstrap resamples for internal validation | ICU admission within 14 days | CASE-plus AUC 0.897 (95% CI 0.842–0.953); CASE alone 0.850; mRS-plus 0.833; mRS alone 0.695 | Hosmer–Lemeshow P values reported for calibration assessment | Useful clinical risk model but not AI per se under this Review’s taxonomy; retrospective single-centre design, retrospective CASE scoring, and lack of external validation limit generalisability |
| Meng et al, 2025 | Original LLM diagnostic proof-of-concept | LLM-assisted AE diagnosis | 100 antibody-positive AE patients from a single centre | Patient level | De-identified clinical data entered stepwise, including MRI, EEG and CSF descriptions | DeepSeek-V3 | Retrospective diagnostic evaluation; no non-AE control group and no external validation | Confirmed antibody-positive AE diagnosis | AE ranked as most likely diagnosis in 49% and within the leading diagnosis/differential in 65%; adding MRI, EEG and CSF information did not significantly improve overall positivity | CI/calibration NR | Disease-specific LLM proof of concept only; confirmed antibody-positive cases, absence of non-AE controls and seronegative AE, and no prospective or external validation preclude inference about real-world diagnostic accuracy |

**Note.** Study characteristics, validation strategies, and performance estimates are reported as described in the cited publications. NR indicates not reported. Studies based exclusively on conventional regression modelling are labelled as AI-adjacent rather than AI per se, consistent with the methodological taxonomy used in this Review. Post-hoc attribution methods such as SHAP are recorded descriptively and are not treated as evidence of validated interpretability. Calibration is reported only where applicable and available. The final column provides a narrative design-based appraisal rather than a formal risk-of-bias assessment.

**Abbreviations:** AE, autoimmune encephalitis; AUC, area under the receiver-operating characteristic curve; CASE, Clinical Assessment Scale in Autoimmune Encephalitis; CI, confidence interval; CSF, cerebrospinal fluid; DL, deep learning; DWI, diffusion-weighted imaging; EEG, electroencephalography; HC, healthy control; HSVE, herpes simplex virus encephalitis; ICU, intensive care unit; LLM, large language model; ML, machine learning; MRI, magnetic resonance imaging; mRS, modified Rankin Scale; NMDAR, N-methyl-D-aspartate receptor; NR, not reported; RF, random forest; SHAP, Shapley additive explanations; SVM, support vector machine.
