# Supplementary material for: Artificial intelligence in central nervous system autoimmune diseases: from clinical decision support to mechanistic discovery and translation
Source: EMBO Mol Med. 2026 Oct 13;18(11):4179–99. doi: 10.1038/s44321-026-00527-0 (PMC13748597; doi:10.1038/s44321-026-00527-0)
Supplement: Supplementary file 6 [file 44321_2026_527_MOESM5_ESM.docx]

Table EV5. Detailed characteristics, validation strategies, and reported performance of studies relevant to AI and computational modelling in myelin oligodendrocyte glycoprotein antibody-associated disease.

| Study | Study type | Clinical task | Cohort and setting | Unit of analysis | Input data | Model / approach | Development and validation | Reference standard / comparator | Main reported performance | Calibration / uncertainty reporting | Key limitation |
| --- | --- | --- | --- | --- | --- | --- | --- | --- | --- | --- | --- |
| Xu et al, 2024; Yeh WZ et al, 2026 | Clinical prognostic score; AI-adjacent | Relapse-risk stratification | Development: 188 patients and 612 treatment episodes; external validation in a 284-patient UK cohort | Treatment-episode and patient level | Clinical characteristics, attack phenotype, treatment-related variables | MOG-AR clinical relapse-risk score | Prospective Chinese registry with internal validation followed by independent Monash–Oxford external validation | Subsequent relapse | Development HR 3.285 (95% CI 1.473–7.327) for grade 4 versus grade 1; external AUC 0.67 (0.61–0.73) for score and 0.58 (0.52–0.63) for grade | External O/E ratio 0.59 (95% CI 0.51–0.68); calibration-in-the-large −1.15 (−1.40 to −0.91), indicating overprediction | Independent external validation is a major strength, but repeated treatment episodes require appropriate dependence handling; modest discrimination and overprediction limit transportability, and relapse-risk prediction does not identify which maintenance therapy will be beneficial |
| Cheng et al, 2025 | Statistical/regularised regression modelling; AI-adjacent | One-year relapse-risk prediction | 88 MOGAD patients from a single centre; 29 relapsed within 1 year; training n=70, validation n=18 | Patient level | Clinical characteristics including sex, phenotype, antibody titre and maintenance immunosuppression | Univariate analysis, LASSO, multivariable logistic regression and nomogram | Random 80%/20% internal training/validation split; calibration and decision-curve analyses | One-year relapse | AUC 0.866 in training and 0.864 in validation; reported 2-year AUC 0.817 (95% CI 0.706–0.928) | Calibration curve and decision-curve analysis reported | Very small single-centre validation set and short follow-up increase overfitting risk; treatment-related predictors may reflect confounding by indication and should not be interpreted as evidence for treatment selection |
| Huang et al, 2022 | Original DL diagnostic study | Three-disease differentiation | 290 patients: 67 MS, 162 AQP4-IgG-positive NMOSD, and 61 MOGAD | Patient level | Brain and/or spinal-cord MRI | MIL-CoaT Transformer | Retrospective single-centre development with 4:1 development/test split; no geographically external validation | Clinical diagnosis; two neuroradiologists | MOGAD AUC 0.803 (95% CI 0.629–0.949); MS 0.933; NMOSD 0.942; overall multiclass accuracy 81.4% versus 64.4% and 74.6% for radiologists | Disease-specific AUC CIs reported; calibration NR | MOGAD discrimination was lower than for MS or NMOSD; single-centre development and lack of external validation limit transportability to diagnostically uncertain populations |
| Luo et al, 2024 | Original radiomics/ML diagnostic study | MOGAD-versus-NMOSD and three-disease differentiation | 212 brain MRI scans from 63 MS, 87 AQP4-IgG-positive NMOSD, and 45 MOGAD patients; >5,000 lesions analysed | Patient and lesion level | Multi-lesion brain MRI radiomics and spatial-distribution features | Random forest and logistic-regression pipeline | Four-fold patient-level cross-validation; no geographically external validation | Clinical diagnosis; three radiologists | MOGAD-versus-NMOSD AUC 0.880 ± 0.064, accuracy 0.828 ± 0.032; three-disease accuracy 75.0% versus 47.6%, 56.6%, and 66.0% for radiologists | Repeated-fold variability reported; calibration NR | Combining lesion and spatial information improved discrimination, but retrospective single-centre data, lack of external validation, and restriction to brain MRI lesions limit clinical transportability |
| Ding et al, 2024c | Original radiomics/ML diagnostic study | Paediatric MOGAD-versus-non-MOGAD acute demyelinating syndromes | 132 children: 66 MOGAD and 66 non-MOGAD; training n=71, internal test n=30, external test n=31 | Lesion and patient level | T1WI, T2WI, T2-FLAIR, ADC and SIR MRI features | Multi-sequence radiomics with SVM; lesion-level fusion followed by subject-level modelling | Internal and external testing; comparison with clinical models and two radiologists | Clinical diagnosis | Subject-level AUC 0.844 internally and 0.846 externally; external accuracy 80.65%, sensitivity 93.75%, specificity 66.67%; senior radiologist accuracy 58.06% externally | 95% CIs reported for AUCs; calibration NR | External testing strengthens evidence, but small paediatric test sets, strict MRI quality control and restricted comparator spectrum limit broader applicability |
| Ding et al, 2024b | Original exploratory ML imaging study | White-matter microstructure classification | 28 children with MOGAD and 31 age- and sex-matched healthy controls | Patient level | Diffusion-tensor imaging with automated fibre quantification | Feature selection followed by eight supervised classifiers | Internal model evaluation; no external validation | Healthy controls; clinical correlation with EDSS | All classifiers AUC >0.85; logistic regression achieved best AUC 0.952; selected DTI metrics correlated with EDSS | CI/calibration NR | Small healthy-control case–control design is highly vulnerable to spectrum bias and overfitting; discrimination from healthy controls does not establish diagnostic performance against inflammatory mimics |
| Pakeerathan et al, 2026 | Quantitative OCT study; non-AI | MOGAD-versus-AQP4-IgG-positive NMOSD optic-neuritis differentiation | 111 adults with first acute optic neuritis from 14 centres in eight countries: 83 MOGAD and 28 AQP4-IgG-positive NMOSD | Patient/eye level | Acute-stage pRNFL thickness measured within 14 days of optic neuritis onset | ROC-derived pRNFL threshold | International retrospective multicentre observational evaluation | Clinical diagnosis | pRNFL >115 µm in 73.4% of MOGAD-ON versus 11.1% of NMOSD-ON; cutoff 117.5 µm yielded AUC 0.838, sensitivity 71.1%, specificity 92.9%, PPV 96.7%, NPV 52.0% | Threshold-performance metrics reported; calibration not applicable | Strong geographic representation, but this is a quantitative biomarker rather than an AI model; threshold performance requires prospective validation across OCT devices, acquisition timing, age groups, and broader optic-neuritis mimics |
| Rechtman et al, 2025 | Original SVM exploratory study | Triple-negative optic-neuritis subgroup classification | 103 patients with first optic neuritis: MS n=26, MOGAD n=25, NMOSD n=13, triple-negative ON n=39 | Patient level | Clinical/onset variables | SVM | Retrospective modelling; no external validation | Subsequent clinical classification / subgroup phenotype | Most evolving-MS triple-negative cases clustered with MS, whereas idiopathic triple-negative cases aligned more closely with MOGAD; quantitative SVM performance metrics NR | CI/calibration NR | Clinically relevant seronegative subgroup, but small subgroup sizes, potentially unstable labels, lack of quantitative discrimination metrics and external validation preclude treatment or prognostic use |

**Note.** Study characteristics, validation strategies, and performance estimates are reported as described in the cited publications. NR indicates not reported. Conventional clinical risk scores, regression-based nomograms, and quantitative biomarker studies are labelled as AI-adjacent or non-AI where appropriate, consistent with the methodological taxonomy used in this Review. Commentary articles are distinguished from original modelling studies. Calibration is reported where applicable and available. The final column provides a narrative design-based appraisal rather than a formal risk-of-bias assessment.

**Abbreviations:** ADC, apparent diffusion coefficient; ADEM, acute disseminated encephalomyelitis; AUC, area under the receiver-operating characteristic curve; AQP4-IgG, aquaporin-4 immunoglobulin G; CI, confidence interval; DTI, diffusion-tensor imaging; EDSS, Expanded Disability Status Scale; HR, hazard ratio; LASSO, least absolute shrinkage and selection operator; ML, machine learning; MOGAD, myelin oligodendrocyte glycoprotein antibody-associated disease; MRI, magnetic resonance imaging; MS, multiple sclerosis; NMOSD, neuromyelitis optica spectrum disorder; NPV, negative predictive value; NR, not reported; OCT, optical coherence tomography; O/E, observed-to-expected; ON, optic neuritis; pRNFL, peripapillary retinal nerve fibre layer; PPV, positive predictive value; SVM, support vector machine.
